# Sesquiterpenes From *Oplopanax elatus* Stems and Their Anti-Photoaging Effects by Down-Regulating Matrix Metalloproteinase-1 Expression *via* Anti-Inflammation

**DOI:** 10.3389/fchem.2021.766041

**Published:** 2021-11-04

**Authors:** Jiejing Yan, Mimi Hao, Yu Han, Jingya Ruan, Dandan Zheng, Fan Sun, Huina Cao, Jia Hao, Yi Zhang, Tao Wang

**Affiliations:** ^1^ Tianjin Key Laboratory of TCM Chemistry and Analysis, Tianjin University of Traditional Chinese Medicine, Tianjin, China; ^2^ Institute of TCM, Tianjin University of Traditional Chinese Medicine, Tianjin, China

**Keywords:** *Oplopanax elatus* stems, sesquiterpenes, HaCaT cells, anti-photoaging, matrix metalloproteinase-1, mitogen-activated protein kinase, inflammatory cytokines

## Abstract

In the process of continuing to investigate ultraviolet b (UVB) irradiation protective constituents from *Oplopanax elatus* stems, nine new sesquiterpenes, named as eurylosesquiterpenosides A–D (**1–4**), eurylosesquiterpenols E–I (**5–9**), and ten known ones (**10–19**) were gained. Their structures were established by analysis of their NMR spectroscopic data, and electronic circular dichroism calculations were applied to define their absolute configurations. In addition, UVB induced HaCaT cells were used to study their anti-photoaging activities and mechanism. The results consolidated that compounds **7**, **11**, and **14** could improve the survival rate of HaCaT cells in concentration dependent manner at 10, 25, and 50 μM. Furthermore, western blot assay suggested that all of them could inhibit the expression of matrix metalloproteinase-1 (MMP-1), and increase the level of type I collagen markedly. Compounds **11** and **14** could reduce the phosphorylation of extracellular signal-regulated kinase and p38, respectively. Besides, compounds **7**, **11**, and **14** could significantly down-regulate the expression of inflammation related protein, such as tumor necrosis factor-*α* and cyclooxygenase-2, which indicated that they played anti-photoaging activities by reducing MMP-1 expression *via* down-regulating the production of inflammatory mediators and cytokines in UVB-induced HaCaT cells.

## Introduction

Skin aging is categorized into intrinsic and extrinsic aging. Extrinsic aging (photoaging) is considered to be the most direct cause of it and mainly caused by ultraviolet B (UVB) irradiation (Pittayapruek et al., 2016; Cavinato and Jansen-Dürr, 2017). Overexpression of matrix metalloproteinases (MMPs) and degradation of collagens are the characteristics of UVB induced photoaging (Rittié and Fisher, 2002). Type I collagen (COL1A1) is the most abundant of subtype of collagens. MMP-1 plays a crucial role in the process of photoaging in virtue of major collagenase for COL1A1 degradation (Pittayapruek et al., 2016). As one of inflammatory mediators, mitogen-activated protein kinases (MAPKs), comprising extracellular signal-regulated kinase (ERK), c-Jun NH_2_-terminal kinase (JNK), and p38 are chiefly associated with collagen degradation mediated by MMP-1 (Yang et al., 2020). The activation of MAPKs by increasing the phosphorylation of p38, JNK, and ERK (p-p38, p-JNK, p-ERK) can up-regulate inflammatory cytokines such as nuclear factor kappa B (NF-*κ*B), tumor necrosis factor *α* (TNF-*α*), interleukin 6 (IL-6), and cyclooxygenase-2 (COX-2) (Choi et al., 2020). Their overexpression will activate MMP-1 to accelerate the degradation of collagen, thereby promote photoaging (Parrado et al., 2016; Peng et al., 2020). Thus, anti-inflammation, as well as inhibiting collagen degradation are the main strategies for preventing UVB-induced photoaging.

*Oplopanax elatus* Nakai belongs to *Oplopanax* genus (Araliaceae family). It was reviewed to contain various constituents such as volatile oil, phenolic acids, lignans, quinic acid esters, steroids, and aliphatic acids, and the stem of it was reported to exhibit anti-aging effect (Yan et al., 2021). Moreover, our previous study demonstrated that phenolic acids obtained from it had anti-photodamage activity, too (Han et al., 2021). We hypothesize there are other components may exhibit benefits for the skin photodamage. Then, the other constituents in the stems of *O. elatus*, along with their activities and mechanisms against photoaging induced by UVB irradiation in HaCaT cells were continue to be investigated.

## Results and Discussion

### Structural Elucidation

**FIGURE 1:**
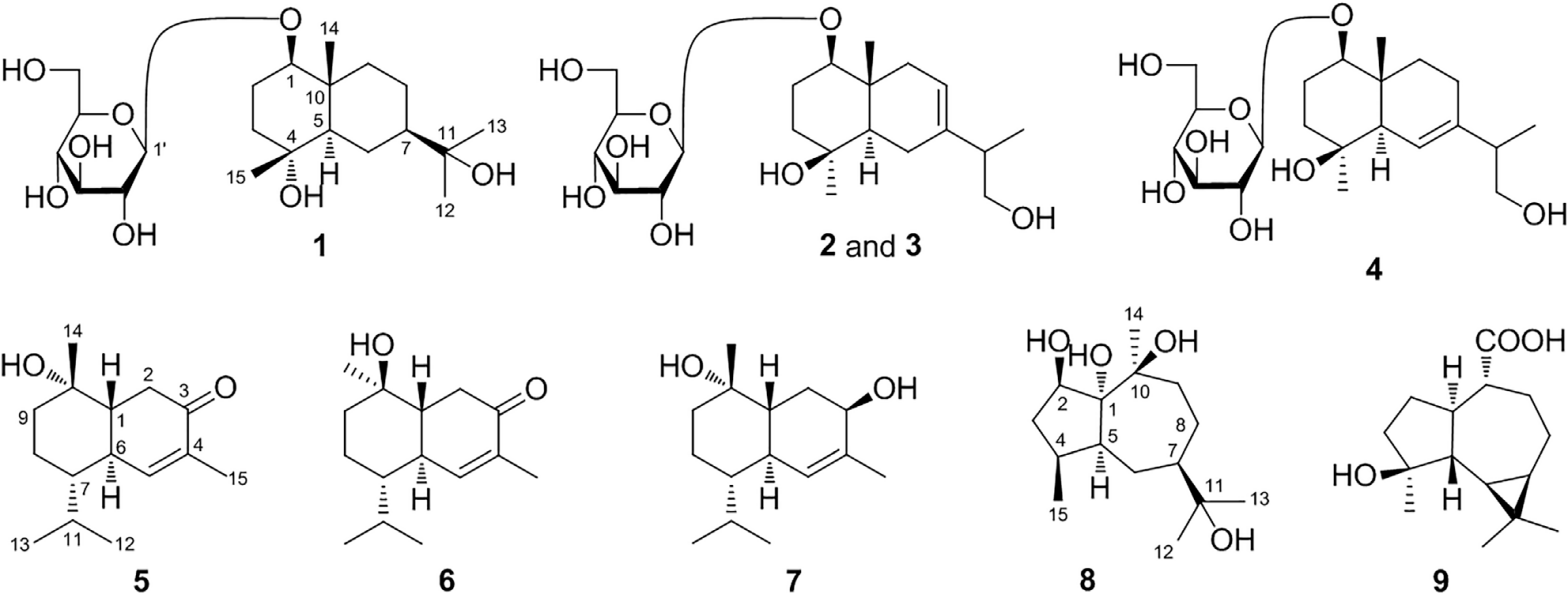
The structures of new sesquiterpenes **1**–**9**.

**FIGURE 2:**
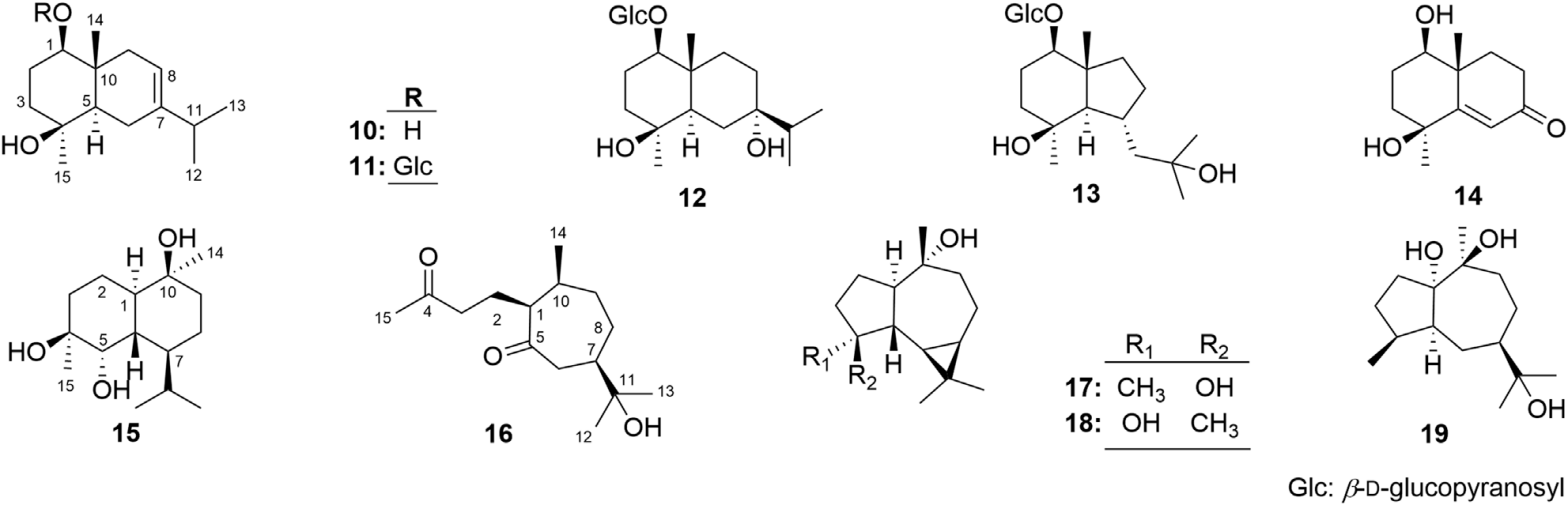
The structures of known compounds **10**–**19**.

19 sesquiterpenes, including nine new ones, named as eurylosesquiterpenosides A–D (**1**–**4**), eurylosesquiterpenols E**–**I (**5**–**9**) (Figure 1), and ten known ones, oplodiol (**10**) (Ono et al., 2008), 1(*R*),4*β*-dihydroxy*-trans*-eudesm-7-ene-1-*O*-β-d-glucopyranoside **(11)** (Lee et al., 2010), massonside B (**12**) (Xiao et al., 2016), massonside A (**13**) (Xiao et al., 2016), (1*R*,4*S*,10*R*)10,11-dimethyl-dicyclohex-5(6)-en-1,4-diol-7-one (**14**) (Elmasri et al., 2016), cadinane-4*β*,5*α*,10*β*-triol (**15**) (Kuo et al., 2003; Fang et al., 2006), 7-*epi*-11-hydroxychabrolidione A (**16**) (Pereira et al., 2012), (—)-4*α*,7*β*-aromaden-dranediol (**17**) (Beechan et al., 1978), aromadendrane-4*α*,10*α*-diol (**18**) (Moreira et al., 2003), stachytriol (**19**) (Soliman et al., 2007) (Figure 2) were isolated from the stems of *O. elatus*. The structures of them were identified by the comprehensive application of UV, IR, NMR, (*α*)_D_, MS, as well as electronic circular dichroism (ECD) spectra. Among them, **11**–**19** were obtained from *Oplopanax* genus for the first time.

**FIGURE 3:**
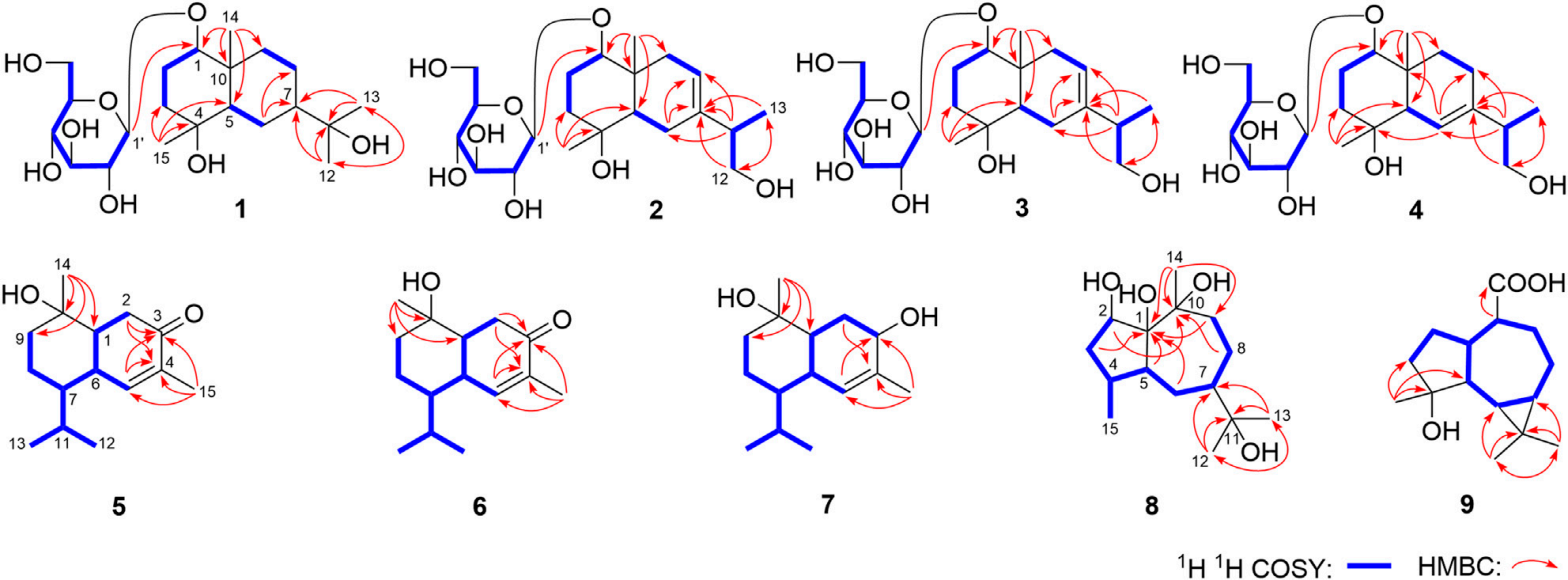
The main ^1^H ^1^H COSY and HMBC correlations of **1–9**.

**FIGURE 4:**
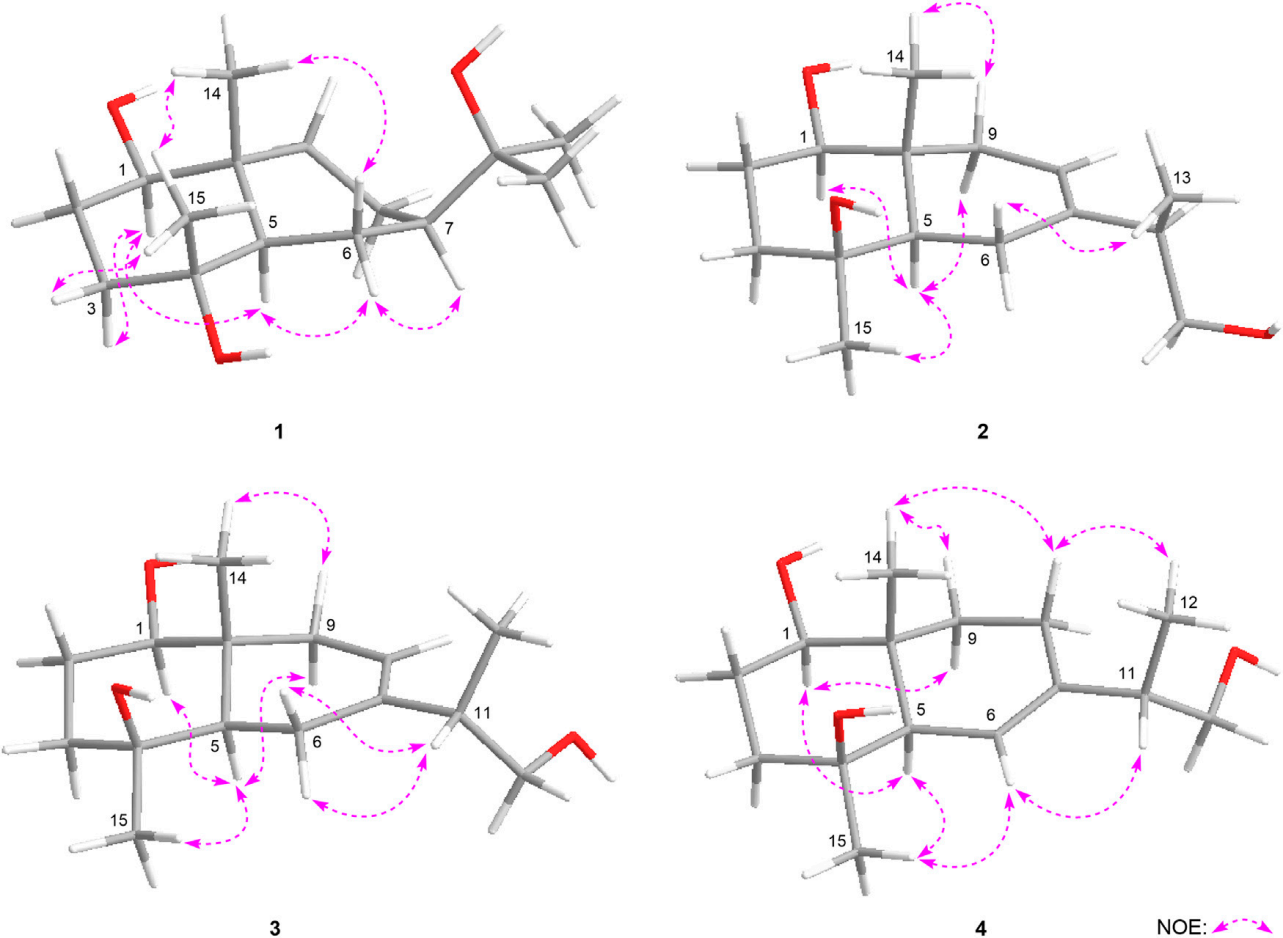
The main NOE correlations of the aglycons of **1–4**.

Eurylosesquiterpenoside A (**1**) was isolated as a white powder and afforded a pseudo-molecular ion peak at *m/z* 463.25449 (M + COOH)^−^ (calcd for C_22_H_39_O_10_, *m/z* 463.25377) in the ESI-Q-Orbitrap MS, which was compatible with a molecular formula of C_21_H_38_O_8_. A combined ^1^H, ^13^C NMR (Table 1) and HSQC spectra analysis revealed the presence of four methyl [*δ*_H_ 0.91, 1.10, 1.16, 1.18 (3H each, all s, H_3_-14, 15, 12, 13)], one oxygenated methine [*δ*_H_ 3.41 (1H, dd, *J* = 4.0, 11.5 Hz, H-1)], two quaternary carbon substituted with oxygen [*δ*_C_ 72.5 (C-4), 73.6 (C-11)], five methene, two methine, and one quaternary carbon. The moieties of “–O–CH–CH_2_–CH_2_–” and “–CH–CH_2_–CH–CH_2_–CH_2_–” were deduced from the proton and proton correlations displayed in its ^1^H ^1^H COSY spectrum (Figure 3). The HMBC cross-peaks from H_3_-12 to C-7, C-11, C-13; H_3_-13 to C-7, C-11, C-12; H_3_-14 to C-1, C-5, C-9, C-10; H_3_-15 to C-3–5 (Figure 3) made the above mentioned funtional gruops and moieties connected together, and suggesting it was an eudesmane type sesquiterpene. Meanwhile, the presence of one anomeric carbon signal at *δ*_C_ 102.0, along with other oxygenated carbon signals in the region of *δ*_C_ 63.0–78.3 in its ^13^C NMR spectrum, indicated there was a monosaccharide moiety. After hydrolyzing **1** with 1 M HCl, the product was analyzed by using HPLC with optical rotation detector (Yoshikawa et al., 2007), and showed positive peak at 10.3 min, which was identical to that of d-glucose standard (10.4 min) (Supplementary Figure S109). Moreover, the monosaccharide was determined to be one β-d-glucopyranose inferring from the large coupling constant of the anomeric proton at *δ* 4.29 (1H, d, *J* = 8.0 Hz, H-1′). Furtherly, the relative configuration of its aglycon was elucidated by the NOE correlations between *δ*_H_ 3.41 (H-1) and *δ*_H_ 1.27 (H-5), 1.47 (H*α*-3); *δ*_H_ 1.76 (H*β*-3) and *δ*_H_ 1.10 (H_3_-15); *δ*_H_ 1.93 (H*α*-6) and *δ*_H_ 1.27 (H-5), 1.31 (H-7); *δ*_H_ 0.91 (H_3_-14) and *δ*_H_ 1.10 (H_3_-15), 1.12 (H*β*-6) (Figure 4). Furthermore, the HMBC correlation from H-1′ to C-1 supported the assumption that the *β*-d-glucopyranosyl attached to C-1. Both the planar structure and relative configuration were the same as those of the known compounds, boarioside (Munoz et al., 1995) and pterodontoside F (Zhao et al., 1997). But there were great differences in their NMR signals. Lots of research results suggested that 10-methyl and 7-isopropyl was *cis* configuration when *δ*_C-5_ and *δ*_C-7_ were 54 ± 2 and 50 ± 1, respectively; while they would be *trans*-conformed to each other when *δ*_C-5_ and *δ*_C-7_ were 49 ± 1 and 42 ± 1, respectively (Kesselmans et al., 1991; Ando et al., 1994; Shimoma et al., 1998; Zhu et al., 2007). Herein, *δ*_C-5_ and *δ*_C-7_ were 54.5 and 50.8 for eurylosesquiterpenoside A (**1**), respectively, suggesting that the relative configuration of 10-methyl and 7-isopropyl was *cis*. It was consistent with the NOE analytical result. The *δ*_C-5_ and *δ*_C-7_ in boarioside were 48.6 and 43.0 (Munoz et al., 1995), while those of pterodontoside F were 48.4 and 42.6, respectively (Zhao et al., 1997). It further indicated that the structure determination of boarioside and pterodontoside F were mistake. The configuration of pterodontoside F’s aglycon (pterodontriol B) had proved by single crystal diffraction, and its 10-methyl and 7-isopropyl should be *trans*-, instead of *cis*-form (Zhu et al., 2007). Therefore, the relative configuration of eurylosesquiterpenoside A (**1**) was firstly clarified as 1*R* ^*^,4*R* ^*^,5*R* ^*^,7*R* ^*^,10*R* ^*^ though its planar structure had been reported.

**TABLE 1:** ^13^CNMR (125 MHz) data for compounds **1–4** in CD_3_OD.

No.	1	2	3	4	No.	1	2	3	4
1	86.5	86.7	86.8	85.5	12	27.0	67.1	67.1	66.6
2	25.6	23.8	23.8	24.1	13	27.4	16.9	16.2	16.6
3	41.7	40.2	40.2	39.8	14	14.5	13.1	13.1	13.1
4	72.5	71.4	71.4	71.5	15	22.6	29.9	29.9	29.5
5	54.5	48.3	48.2	51.9	1′	102.0	101.9	101.9	102.1
6	22.6	24.5	24.2	121.3	2′	75.2	75.1	75.1	75.2
7	50.8	139.1	138.1	141.4	3′	78.3	78.3	78.3	78.3
8	23.2	120.8	121.3	23.4	4′	71.9	72.0	71.9	71.9
9	41.9	41.9	42.0	36.2	5′	77.8	77.8	77.8	77.8
10	39.6	38.3	38.2	38.8	6′	63.0	63.1	63.0	63.0
11	73.6	44.6	45.0	45.0	—	—	—	—	—

Eurylosesquiterpenoside B (**2**) was obtained as white powder with negative optical rotation [(*α*)_D_^25^–34.9, MeOH]. It had a molecular formula of C_21_H_36_O_8_ assigned basing on the carboxyl adduct (M + COOH)^−^ at *m/z* 461.23981 in the ESI-Q-Orbitrap MS. d-glucose was detected from its acid hydrolysate by using the similar method as compound **1** (Yoshikawa et al., 2007). Its ^1^H and ^13^C NMR (Table 1) spectra indicated the existence of one *β*-d-glucopyranosyl [*δ*_H_ 4.32 (1H, d, *J* = 7.5 Hz, H-1′); *δ*_C_ 63.1, 72.0, 75.1, 77.8, 78.3, 101.9]. There were twenty-one carbon signals in its ^13^C NMR spectrum. Apart from the above six carbon signals of *β*-d-glucopyranosyl, fifteen carbon signals were remaining. Most of them were located in the high resonance region, suggesting that compound **2** was a sesquiterpenoid glycoside. Its ^1^H, ^13^C NMR spectra showed the signals related to the protons of three methyl [*δ*_H_ 1.01, 1.14 (3H each, both s, H_3_-14, 15), 1.04 (3H, d, *J* = 7.0 Hz, H_3_-13)], one methene bonded to oxygen [*δ*_H_ 3.38 (1H, dd, *J* = 5.5, 10.5 Hz), 3.57 (1H, dd, *J* = 6.0, 10.5 Hz), H_2_-12], one oxygenated methine [*δ*_H_ 3.42 (1H, dd, *J* = 4.0, 11.5 Hz, H-1)], as well as one olefinic proton signal [*δ*_H_ 5.37 (1H, d, *J* = 4.5 Hz, H-8)] in its aglycon. Five fragments showed by the bold line in Figure 3 were denoted according to the proton and proton correlations. Moreover, the HMBC cross-peaks were observed from H_2_-6 to C-7, C-8; H-11 to C-6–8; H_2_-12 to C-7, C-13; H_3_-13 to C-7, C-12; H_3_-14 to C-1, C-5, C-9, C-10; H_3_-15 to C-3–5; H-1′ to C-1 (Figure 3). Then, the planar structure of two was determined, which was the same as those chrysantiloboside (Shin et al., 2012) and iwayoside C (Ding et al., 2011). The NOE cross-peaks between *δ*_H_ 1.30 (H-5) and *δ*_H_ 1.14 (H_3_-15), 1.90 (H*α*-9), 3.42 (H-1); *δ*_H_ 2.14 (H*β*-9) and *δ*_H_ 1.01 (H_3_-14) (Figure 4) consolidated that 1-OH, 4-OH, H-5, and 14-CH_3_ was in *β*, *β*, *α*, and *β* orientation, respectively. The resonance signals for protons and carbons in C-1–5, 9, 10 were consistent with those of 1(*R*),4*β*-dihydroxy-*trans*-eudesm-7-ene-1-*O*-*β*-d-glucopyranoside (11) (Lee et al., 2010)**,** which implied the absolute configurations of C**-**1, 4, 5, 10 of eurylosesquiterpenoside B (**2**) were *R*, *S*, *R*, *R*, respectively. .

Eurylosesquiterpenoside C (**3**), white powder with (*α*)_D_^25^ –29.3 (in MeOH). The same molecular formula, C_21_H_36_O_8_ [*m/z* 461.23886 (M + COOH)^−^; calcd for C_22_H_37_O_10_, 461.23812] as compound **2** was revealed by the ESI-Q-Orbitrap MS analysis. The ^1^H, ^13^C NMR (Table 1), and ^1^H ^1^H COSY, HSQC, as well as HMBC spectra suggested their planar structure were also same. The ^13^C NMR data of C-1–5, 10, 14, 15, and 1′–6′, as well as the NOE correlations for H-1, 5, 9, 14, 15 in **3** were very consistent with those of **2** (Figure 4), indicating the absolute configurations of **C-**1, 4, 5, 10 were identical to **2** (1*R*,4*S*,5*R*,10*R*). However, the retention times of them were 24.4 min for **2** and 19.5 min [column: Cosmosil 5C18-MS-II (4.6 mm i. d. × 250 mm, 5 µm); mobile phase: CH_3_CN-1% HAc (16:84, v/v); flow rate: 0.7 ml/min, Supplementary Figure S25] for **2** and **3**, respectively. Moreover, the *Δδ*_C_ of **3** and **2** were −0.3, −1.0, +0.5, +0.4, −0.7 at C-6, 7, 8, 11, 13, respectively, which indicated the difference between them might be caused by the configuration difference of C-11.

**FIGURE 5:**
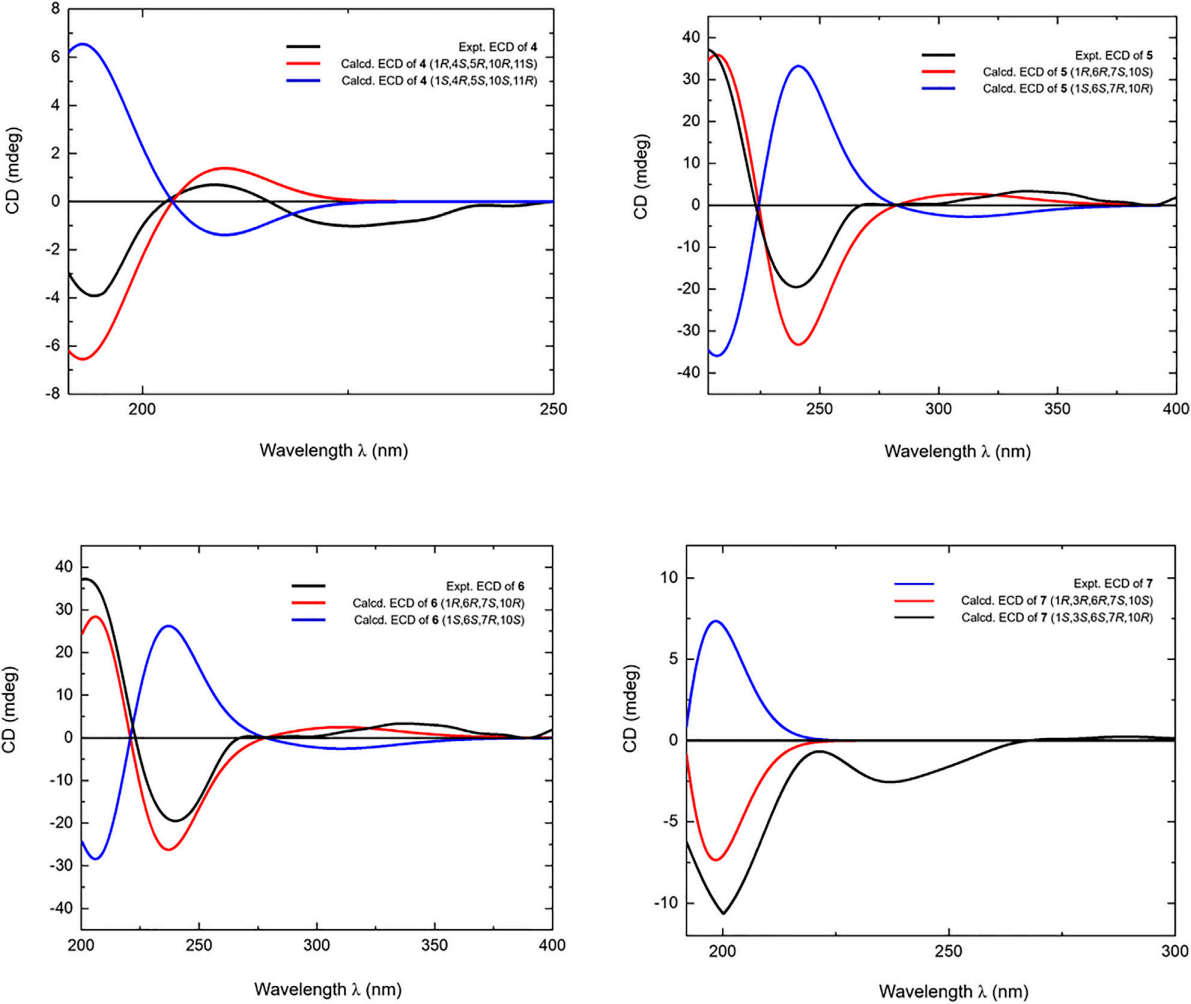
Calculated and experimental ECD spectra of **4–7**.

It was worth pointing out that the structures of reported compounds iwayoside C (Ding et al., 2011) and chrysantiloboside (Shin et al., 2012) were identical since the consistency of their nuclear magnetic resonance. Moreover, though the planar structures of **2** and **3** were the same as them, there were no relevant reports on the determination of absolute configuration had been found in the literature. Eurylosesquiterpenoside D (**4**) had the same molecular formula, C_21_H_36_O_8_, as compounds **2** and **3**. While, comparing with the ^1^H and ^13^C NMR (Table 1) spectra of **2** and **3**, it was found that the NMR resonance of C-7 increased significantly. Meanwhile, their coupling and splitting information were also different [**2**: *δ*_H_ 5.37 (1H, d, *J* = 4.5 Hz), **3**: *δ*_H_ 5.38 (1H, d, *J* = 5.5 Hz), **4**: *δ*_H_ 5.56 (1H, br. s)]. The correlations between *δ*_H_ 1.87 (H-5) and *δ*_H_ 5.56 (H-6); *δ*_H_ 1.93, 1.99 (H_2_-8) and *δ*_H_ 1.26, 2.05 (H_2_-9) and correlations from *δ*_H_ 5.56 (H-6) to *δ*_C_ 71.5 (C-4) (Figure 3) suggested the olefinic bond replaced between C-6 and C-7, rather than between C-7 and C-8. According to the NOE correlations between *δ*_H_ 1.87 (H-5) and *δ*_H_ 1.20 (H_3_-15), 3.44 (H-1); *δ*_H_ 3.44 (H-1) and *δ*_H_ 1.24 (H*α*-9); *δ*_H_ 2.05 (H*β*-9) and *δ*_H_ 1.01 (H_3_-14) (Figure 4), the relative configurations of H-1, H-5, H-9, 14-CH_3_, and 15-CH_3_ were revealed. The chemical shifts of protons in C-1–5, 9, and 10 were almost identical to the known compound, 1(*R*),4*β*-dihydroxy-*trans*-eudesm-6-ene-1-*O*-*β*-d-glucopyranoside (Lee et al., 2010). Then, the absolute configurations at C-1, 4, 5, and 10 were determined as *R*, *S*, *R*, and *R*, respectively. Furtherly, the calculated ECD spectrum was identical to that of experimental one (Figure 5) (Nugroho and Morita, 2014; Frisch et al., 2019; Takanawa, 2019). Then, the absolute configuration of **4** was elucidated as 1*R*,4*S*,5*R*,10*S*.

**FIGURE 6:**
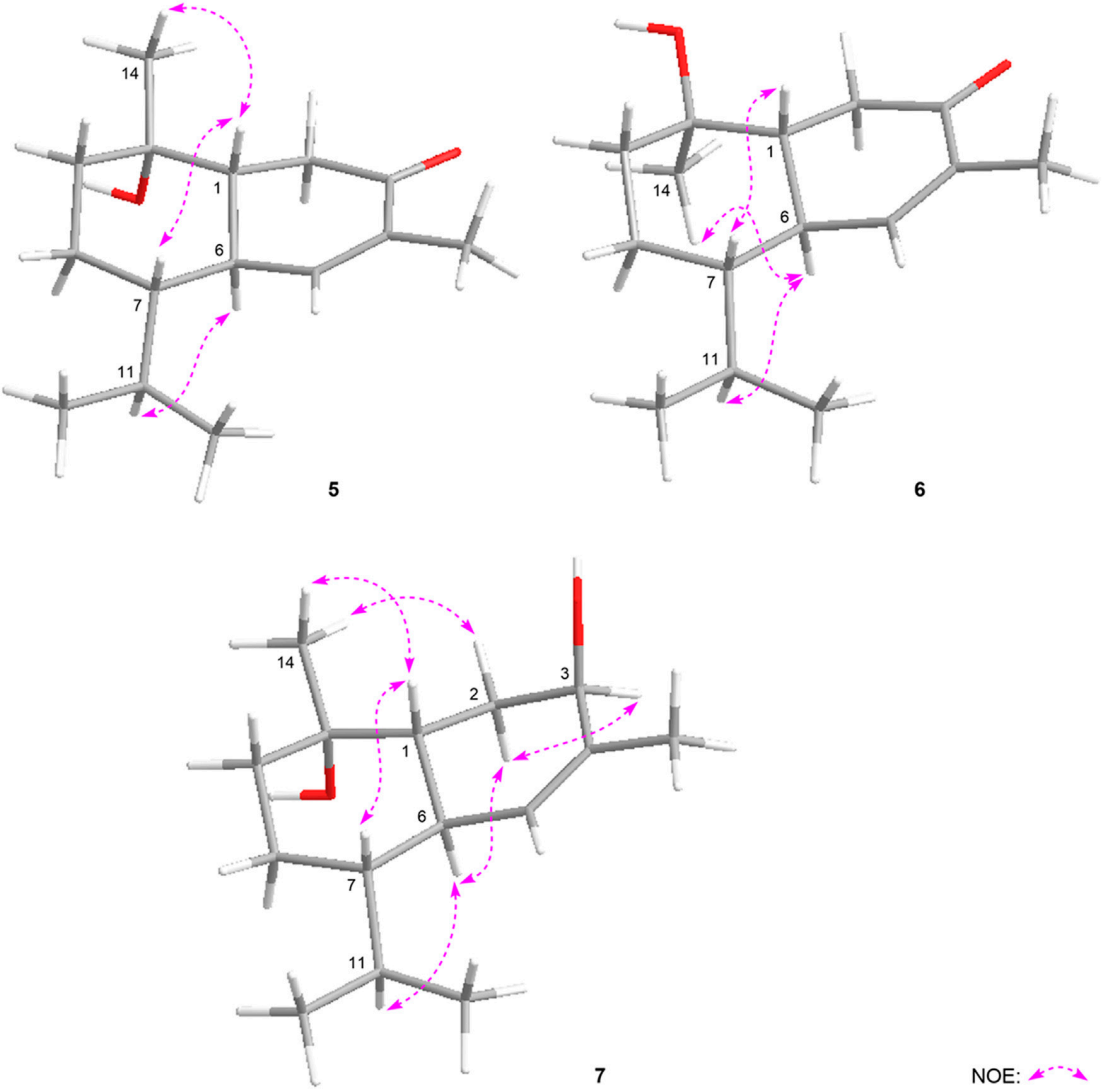
The main NOE correlations of **5–7**.

Eurylosesquiterpenol E (**5**) was isolated as white powder with negative optical rotation [(*α*)_D_^25^ –71.8, MeOH]. It afforded a pseudo-molecular ion peak at *m/z* 281.17557 (M + COOH)^−^ (calcd for C_16_H_25_O_4_, *m/z* 281.17474) in the ESI-Q-Orbitrap MS, which was compatible with a molecular formula of C_15_H_24_O_2_. The ^1^H, ^13^C NMR (Table 2) spectra implied the presence of four methyl signals at *δ*_H_ 0.85, 0.98 (3H each, both d, *J* = 7.0 Hz, H_3_-12, 13), 1.19 (3H, s, H_3_-14), 1.78 (3H, br. s, H_3_-15) and one *α*,*β*-unsaturated ketone at *δ*_H_ 6.89 (1H, br. s, H-5) and *δ*_C_ 134.9 (C-4), 147.4 (C-5), 200.7 (C-3). Fifteen carbon signals were displayed in its ^13^C NMR spectrum, which suggested it was also a sesquiterpene. The proton and proton cross-peaks between H-1 and H_2_-2; H-6 and H-1, H-5, H-7; H_2_-8 and H-7, H_2_-9; H-11 and H-7, H_3_-12, H_3_-13 proved the existence of moieties showed with the bold lines in Figure 3. And its planar structure was consolidated by the long-range correlations from H_2_-2, H-5 to C-3, C-4; H_3_-14 to C-1, C-9, C-10; H_3_-15 to C-3–5, which was a cadinane type sesquiterpene. Moreover, the relative configuration was revealed by the NOE correlations between *δ*_H_ 1.72 (H-1) and *δ*_H_ 1.16 (H-7), 1.19 (H_3_-14); *δ*_H_ 2.25 (H-11) and *δ*_H_ 2.40 (H-6) (Figure 6), and it was identical to that of 3-oxo-*α*-cadinol (Wu et al., 2005). Furthermore, the ECD experiment was developped to clarify its configuration. As result, its experimental ECD was consistent with that of 1*R*,6*R*,7*S*, 10*S* calculated (Figure 5) (Nugroho and Morita, 2014; Frisch et al., 2019; Takanawa, 2019). Then, the absolute configuration of eurylosesquiterpenol E (**5**) was elucidated here firstly.

**TABLE 2:** ^13^C NMR (125 MHz) data for compounds **5**–**9**.

No.	5^*a*^	5^*b*^	6^*a*^	6^*b*^	7^*a*^	7^*b*^	8^*a*^	8^*b*^	9^*a*^	9^*b*^
1	49.9	50.7	51.2	52.0	42.2	43.3	87.1	87.2	48.2	49.2
2	38.5	39.3	38.4	39.1	31.9	33.5	71.3	70.4	28.6	29.5
3	200.7	200.2	200.3	200.2	68.4	67.9	40.9	41.7	41.4	42.3
4	134.9	134.9	135.5	135.9	134.7	136.4	33.2	33.6	80.0	79.7
5	147.4	147.4	146.2	146.5	127.4	126.6	47.0	47.5	53.0	53.3
6	38.7	39.0	41.0	41.1	38.4	38.7	24.4	24.8	28.4	29.4
7	45.0	45.4	45.2	45.5	46.1	46.8	36.5	36.7	27.5	27.9
8	19.4	19.8	21.6	21.8	19.9	20.4	19.3	19.8	24.1	24.5
9	39.9	40.5	41.7	42.2	40.5	41.5	26.9	27.4	31.7	32.6
10	69.7	68.7	71.3	70.1	70.6	69.5	76.1	76.5	53.9	55.6
11	26.4	26.6	26.3	26.3	26.3	26.6	73.9	73.5	21.6	21.5
12	15.3	15.4	15.3	15.3	15.3	15.6	29.2	29.4	28.9	29.0
13	21.3	21.5	21.5	21.6	21.4	21.7	29.3	29.5	16.5	16.7
14	28.0	28.2	21.1	21.4	28.5	29.0	26.6	27.0	180.0	178.8
15	15.9	16.2	16.0	16.2	21.2	22.0	14.9	15.2	25.4	25.7

Determined in ^*a*^CDCl_3_ and ^*b*^C_5_D_5_N.

Eurylosesquiterpenol F (**6**), a white powder with negative optical rotation [(*α*)_D_^25^ –36.0, MeOH]. The MS [*m/z* 281.17548 (M + COOH)^−^; calcd for C_16_H_25_O_4_, *m/z* 281.17474] and NMR (Table 2; Figure 3) spectra implied the planar structure of **6** was consistent with **5**. However, their *Δδ*_C_ of C-1, 8, 9, 10, 14 were +1.3, +2.2, +1.8, +1.6, and −6.9, respectively, which might be caused by the different configuration of 14-CH_3_. The cross-peaks between *δ*_H_ 1.84 (H-1) and *δ*_H_ 1.21 (H-7); *δ*_H_ 2.08 (H-6) and *δ*_H_ 1.17 (H_3_-14), 2.23 (H-11) displaying in its NOSEY spectrum (Figure 6) suggested 14-CH_3_ was in *α* orientation. And the relative configuration of **6** was clarified. Finally, the configuration of 1*R*,6*R*,7*S*,10*R* was determined by the uniformity of its expeimental and calculated ECD spectra (Figure 5).

The molecular formula, C_15_H_26_O_2_, of eurylosesquiterpenol G (**7**) was assigned basing on the carboxyl adduct (M + COOH)^–^ at *m/z* 283.19107 (calcd for C_16_H_27_O_4_, *m/z* 283.19039) in the ESI-Q-Orbitrap MS. Comparing with **5**, **6**, its molecular weight went up by 2Da. Meanwhile, the ^13^C NMR (Table 2) spectrum suggested the disappearence of one carbon signal of *α*,*β*-unsaturated ketone, and the appearence of one oxygenated methine [*δ*_H_ 4.34 (1H, br. s, H-3)]. Then, **7** was speculated to be formed by the reduction of 3-carbonyl. The correlations displayed in its ^1^H ^1^H COSY and HMBC spectra (Figure 3) fully proved the correctness of the speculation. The chemical shift of C-14 (*δ*_C_ 29.0) beling closer to compound **5** (**5**: *δ*_C_ 28.2; **6**: *δ*_C_ 21.4) indicated the configuration of C-10 was *S*. Moreover, according to the NOE correlations between *δ*_H_ 1.83 (H-1) and *δ*_H_ 1.10 (H-7), 1.45 (H_3_-14); *δ*_H_ 1.45 (H_3_-14) and *δ*_H_ 2.49 (H*β*-2); *δ*_H_ 2.06 (H*α*-2) and *δ*_H_ 2.46 (H-6), 4.34 (H-3) (Figure 6), the configuration of 1*R*,3*R*,6*R*,7*S*,10*S* was clarified. It was confirmed by the consistency of expermental and calculated ECD results (Figure 5) (Nugroho and Morita, 2014; Frisch et al., 2019; Takanawa, 2019).

**FIGURE 7:**
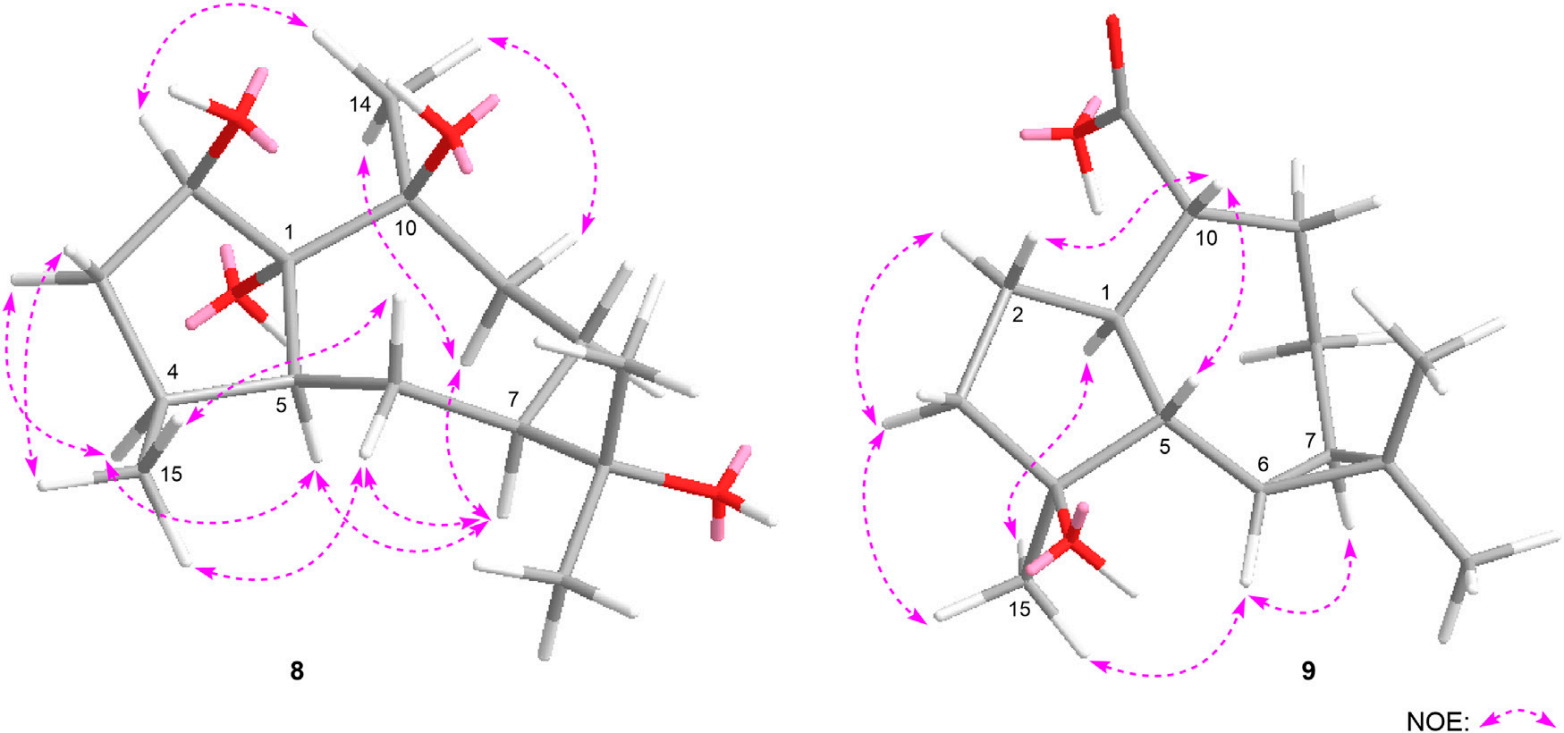
The main NOE correlations of **8**, **9**.

The molecular formula of eurylosesquiterpenol H (**8**) was determined as C_15_H_28_O_4_ by ESI-Q-Orbitrap MS spectrometry. The ^1^H, ^13^C NMR (Table 2) spectra suggested the presence of four methyl [*δ*_H_ 1.10, 1.17, 1.30 (3H each, all s, H_3_-14, 12, 13), 0.89 (3H, d, *J* = 7.0 Hz, H_3_-15)] and one oxygnated methine [*δ*_H_ 4.70 (1H, br. d, *ca*. *J* = 9 Hz, H-2)]. Combining the proton and proton correlations and the long-range cross-peaks from H_2_-3, H-4, H_2_-6, H_2_-9, C-1; H-5 to C-1, C-2, C-10; H_2_-8 to C-10; H_3_-12 to C-7, C-11, C-13; H_3_-13 to C-7, C-11, C-12; H_3_-14 to C-1, C-9, C-10 (Figure 3), the planar structure of compound **8** was clarified, which was a guaiane type sesquiterpene. The chemical shifts of C-6–10 (Table 2) were consistent with those of stachytriol (**19**) (Soliman et al., 2007), which indicated that the configurations at C-1, 5, 7, 10 of them were identical. The main difference between **8** and **19** was that C-2 in the former was substituted by the hydroxyl. The NOE correlation between *δ*_H_ 4.70 (H-2) and *δ*_H_ 1.10 (H_3_-14) implied 2-OH was in *β* orientation. Meanwhile, the cross-peaks between other protons (Figure 7) were basically consistent with those of stachytriol (**19**). Consequently, the configuration of eurylosesquiterpenol H (**8**) was denoted as 1R*,2R*,4S*,5S*,7R*,10S*.

Eurylosesquiterpenol I (**9**) was isolated as white powder. Its molecular formula was determined as C_15_H_24_O_3_ with *m/z* 251.16508 (M—H)^−^ (calcd for C_15_H_23_O_3_, *m/z* 251.16417). ^1^H and ^13^C NMR (Table 2) spectra showed three methyl proton signals at *δ*_H_ 1.08, 1.25, 1.45 (3H each, all s, H_3_-12, 13, 15) and one carboxyl carbon signal at *δ*_C_ 178.8 (C-14). The moieties displayed with the bold lines in Figure 3 were determined by the observation of proton and proton correlations in the ^1^H ^1^H COSY spectrum. Moreover, its planar structure was determined by the cross-peaks found from H_3_-12 to C-6, C-7, C-11, C-13; H_3_-13 to C-6, C-7, C-11, C-12; H_3_-15 to C-3–5; H-1 to C-14 (Figure 3). Finally, the NOE correlations between *δ*_H_ 0.56 (H-6) and *δ*_H_ 0.67 (H-7), 1.45 (H_3_-15); *δ*_H_ 1.45 (H_3_-15) and *δ*_H_ 1.70 (H*α*-3), 2.29 (H-1); *δ*_H_ 1.70 (H*α*-3) and *δ*_H_ 2.10 (H*α*-2); *δ*_H_ 1.93 (H*β*-2) and *δ*_H_ 2.53 (H-10); *δ*_H_ 2.53 (H-10) and *δ*_H_ 1.83 (H-5) were observed in its NOESY spectrum (Figure 7). Then, the configuration of eurylosesquiterpenol I (**9**) was elucidated to be 1R*,4S*,5R*,6R*,7R*,10R*.

The structures of known compounds **10**–**19** were identified by comparing their ^1^H, ^13^C NMR spectroscopic data with those reported in literatures.

### Biological Activity

**FIGURE 8:**
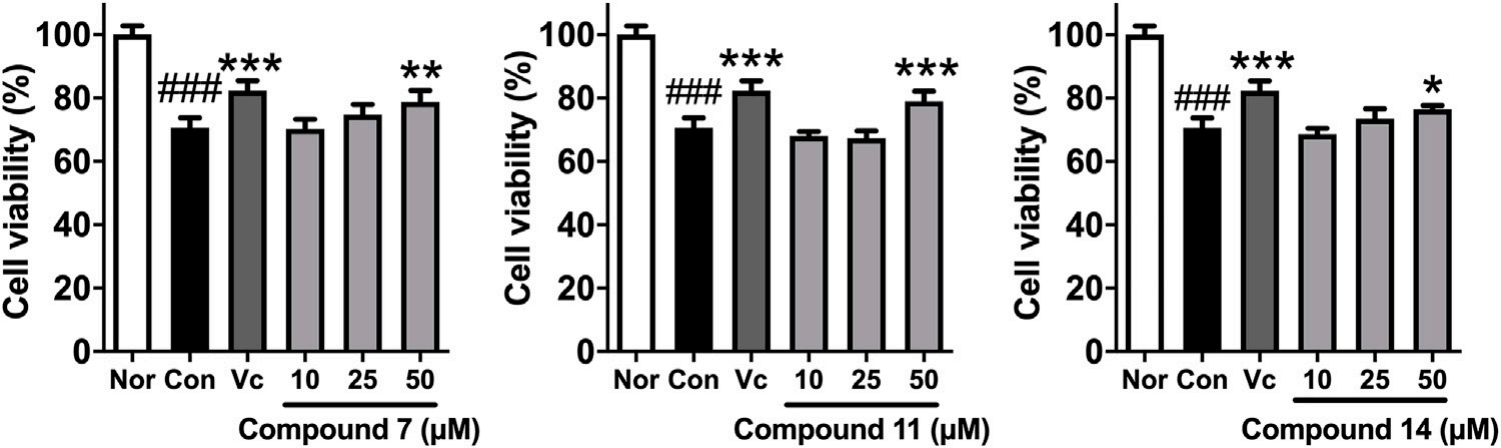
The effects of compounds **7**, **11**, and **14** at concentration of 10, 25, 50 μM on the cell viability of HaCaT cells induced by UVB. Nor: normal group; Con: UVB-induced group; Vitamin C (Vc). Values represent the mean ± SEM of six determinations (^###^ *p* < 0.001 *vs.* Nor; ***p < 0.001, **p < 0.01, and *p < 0.05 *vs.* Con).

In addition, *in vitro* anti-photoaging activities of the obtained sesquiterpenes were evaluated. MTT assay results showed that most of compounds (**2**, **3**, **6**, **7**, **10**, **11**, **13**–**18**) were non-cytotoxic at 50 μM on HaCaT cells (Supplementary Figure S110). Among them, HaCaT cells were pretreated with test samples (50 μM) for 24 h before UVB irradiation individually. Comparing with UVB-induced group (Con), **7**, **11**, and **14** could significantly improve the survival rate of HaCaT cells after UVB irradiation (Table 3). Moreover, the activities of compounds **7**, **11**, and **14** against UVB injury were found to be in concentration dependent manner at 10, 25, and 50 μM (Figure 8).

**TABLE 3:** Cell viabilities of UVB induced HaCaT cells pretreated with **2**, **3**, **6**, **7**, **10**, **11**, **13**–**18**.

No.	Cell viability (%)	No.	Cell viability (%)	No.	Cell viability (%)
Nor	100 ± 2.8	**6**	69.9 ± 2.3	**14**	76.5 ± 1.2*
Con	70.6 ± 3.2^###^	**7**	78.8 ± 3.6**	**15**	66.9 ± 2.5
Vc	80.4 ± 3.9***	**10**	68.2 ± 1.6	**16**	73.4 ± 1.8
**2**	70.9 ± 0.6	**11**	79.0 ± 3.2***	**17**	70.0 ± 1.1
**3**	67.2 ± 0.6	**13**	69.3 ± 0.7	**18**	71.4 ± 2.1

Nor: normal group; Con: UVB-induced group; Vitamin C (Vc). Cell viability: percentage of normal group (set as 100%). Final concentration was 50 μM for Vc and assayed compounds. Values represent the mean ± SEM of six determinations (^###^ *p* < 0.001 *vs.* Nor; ***p < 0.001, **p < 0.01, and *p < 0.05 *vs.* Con).

As we introduced previously, the level of MMP-1 will be increased after UVB irradiation, and the degradation of COL1A1 will be caused at the same time in HaCaT cells. The process is related to the up-regulation of inflammatory mediator, MAPKs, and inflammatory cytokines such as TNF-*α* and COX-2. Therefore, the expressions of above proteins were evaluated by using western blot assay to study the anti-photoaging mechanism of compounds **7**, **11**, and **14**.

**FIGURE 9:**
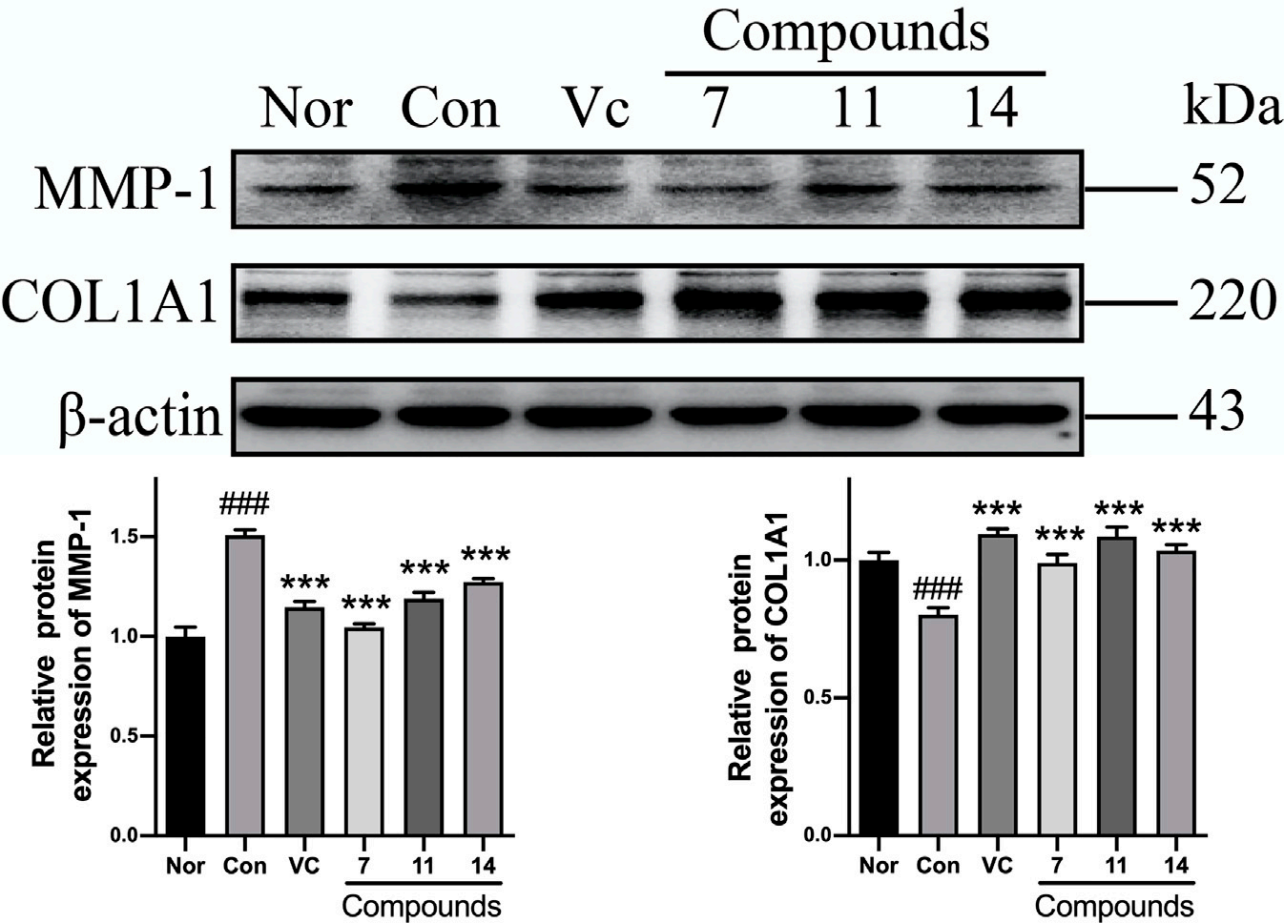
The influence of compounds **7**, **11**, and **14** at concentration of 50 *μ*M on the protein of MMP-1 and COL1A1 in HaCaT cells. Nor: normal group; Con:UVB-induced group; Vitamin C (Vc). Values represent the mean ± SEM of three determinations (^###^ *p* < 0.001 *vs.* Nor; ***p < 0.001 *vs.* Con).

Comparing with normal group (Nor), the level of MMP-1 was increased and COL1A1 was decreased significantly in Con after UVB irradiation. While, the expression of MMP-1 was significantly decreased by 30, 21, and 16%, and the level of COL1A1 was up-regulated by 24, 36, and 29% in pretreatment of compounds **7**, **11**, and **14**, respectively (Figure 9).

**FIGURE10:**
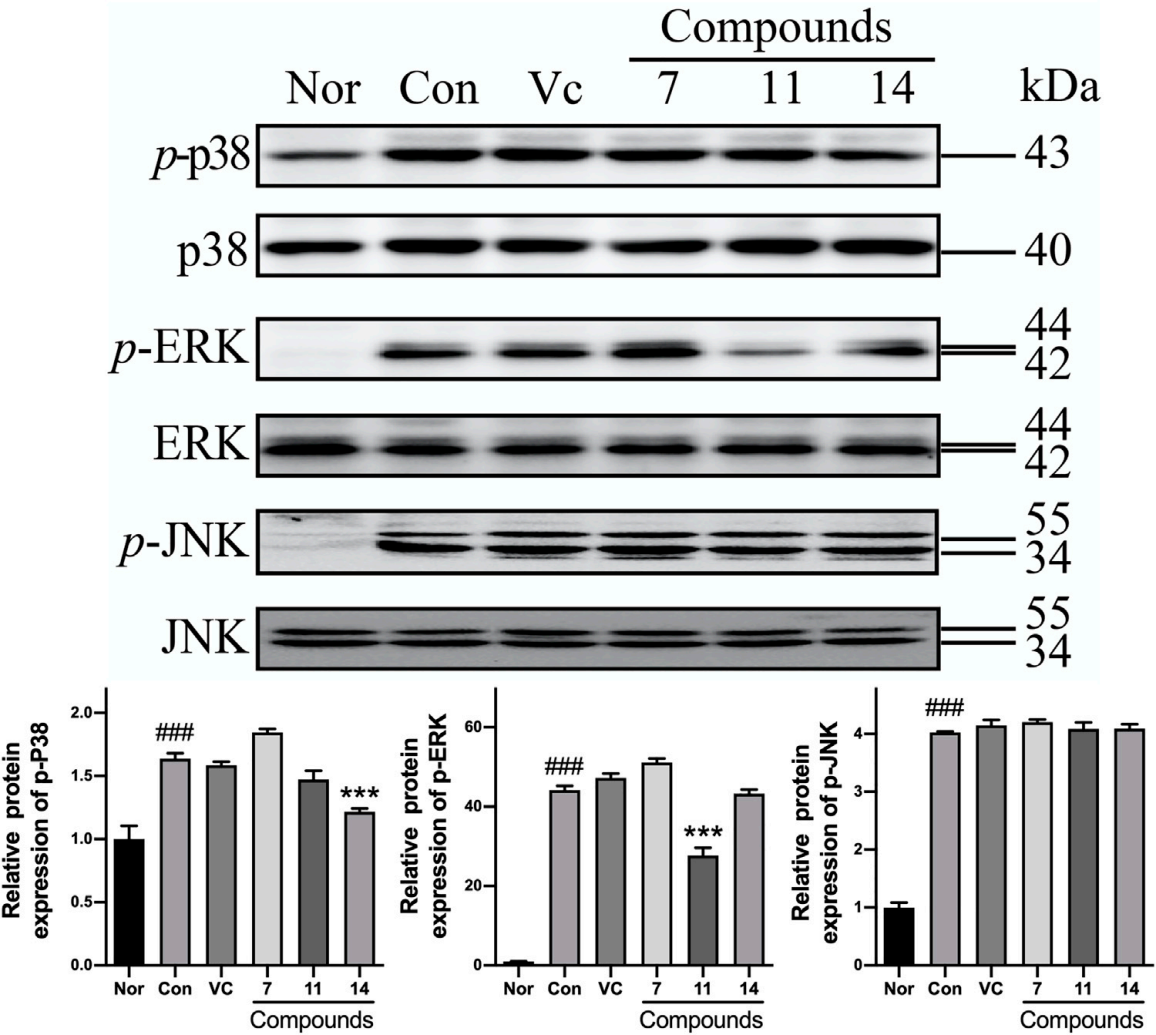
The influence of compounds **7**, **11**, and **14** at concentration of 50 μM on the protein of MAPKs in HaCaT cells. Nor: normal group; Con: UVB-induced group; Vitamin C (Vc). Values represent the mean ± SEM of three determinations (^###^ *p* < 0.001 *vs.* Nor; ***p < 0.001 *vs.* Con).

Meanwhile, the phosphorylations of MAPKs were up-regulated in varying levels in Con comparing with Nor. However, the p-ERK was markedly reduced to 0.62-fold by compound **11**; and the p-p38 could be inhibited to 0.74-fold by **14**. Nevertheless, none of active compounds could prevent up-regulation of p-JNK (Figure 10).

**FIGURE 11:**
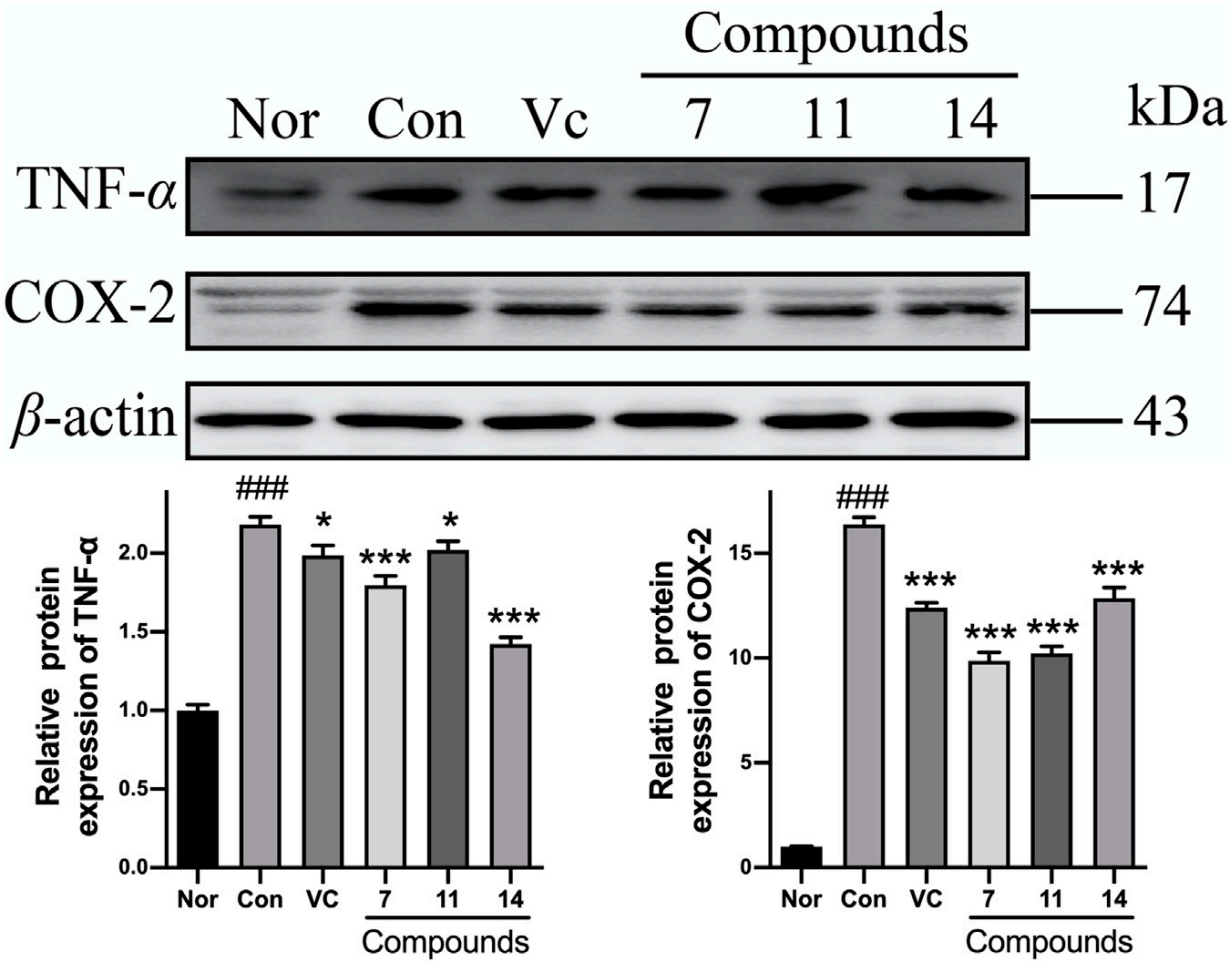
The influence of compounds **7**, **11**, and **14** at concentration of 50 μM on the protein of inflammatory cytokines (TNF-*α* and COX-2) in HaCaT cells. Nor: normal group; Con: UVB-induced group; Vitamin C (Vc). Values represent the mean ± SEM of three determinations (^###^ *p* < 0.001 *vs.* Nor; ***p < 0.001 and *p < 0.05 *vs.* Con).

After pretreating with compounds **7**, **11**, and **14**, the TNF-*α* expression could be significantly reduced by 18, 17, 35%, and the level of COX-2 was decreased by 40, 38, 21% comparing with Con, respectively (Figure 11).

Basing on the above results, the anti-photoaging mechanism of compounds **7**, **11**, and **14** might be related to inhibiting collagen degradation *via* anti-inflammation.

## Materials and Methods

### Experimental Procedures for Phytochemistry Study

#### General Experimental Procedures

NMR spectra were performed on Bruker ascend 600 MHz and/or Bruker ascend 500 MHz NMR spectrometer (Bruker BioSpin AG Industriestrasse 26 CH-8117) with tetramethylsilane as an internal standard. Negative-ion mode ESI-Q-Orbitrap MS were determined on a Thermo ESI-Q-Orbitrap MS mass spectrometer connected to the UltiMate 3000 UHPLC instrument *via* ESI interface (Thermo Scientific). Optical rotations, UV, IR, and ECD spectra were run on a Rudolph Autopol^®^ IV automatic polarimeter (l = 50 mm) (Rudolph Research Analytical, Hackettstown), Varian Cary 50 UV-Vis (Varian, Inc.), Varian 640-IR FT-IR spectrophotometer (Varian Australia Pty Ltd.), and Circular dichroism spectrum (J-815, JASCO company), respectively.

Column chromatorgtaphies (CC) were accomplished on macroporous resin D101 (Haiguang Chemical Co., Ltd.), silica gel (48–75 μm, Qingdao Haiyang Chemical Co., Ltd.), ODS (50 μm, YMC Co., Ltd.), MCI gel CHP 20P (Mitsubishi Chemical Corporation, CHP20/P120), and Sephadex LH-20 (Ge Healthcare Bio-Sciences). HPLC column: Cosmosil 5C18-MS-II (4.6 mm i. d. × 250 and 20 mm i. d. × 250 mm) (5 μm, Nakalai Tesque, Inc.) were used to analysis and separate the constituents, respectively.

Dichloromethane (CH_2_Cl_2_), methanol (MeOH), acetonitrile (CH_3_CN), acetic acid (HAc), and other reagents (chromatographically pure or analytical pure) were purchased from Tianjin Concord Technology Co., Ltd.

#### Plant Material

The stems of *Oplopanax elatus* Nakai were collected from Tonghua city, Jilin province, China, identified by Professor Junyi Zhu (Tonghua Normal University). The voucher specimen (2018121001) was deposited at the Academy of Traditional Chinese Medicine of Tianjin University of TCM.

#### Extraction and Isolation

The 95% EtOH eluate (70.0 g) obtained previously (Han et al., 2021) was fractionated over silica gel using a gradient elution of CH_2_Cl_2_-MeOH (100:0 → 100:1 → 100:3 → 100:7 → 10:1 → 20:3 → 5:1 → 3:1 → 1:1 → 0:100, v/v) to give Fr. 1–Fr. 12. Fraction 5 (289.2 mg) was prepared by pHPLC [MeOH-1% HAc (80:20, v/v)] to yield (1*R*,4*S*,10*R*)10,11-dimethyl-dicyclohex-5(6)-en-1,4-diol-7-one (**14**, 8.4 mg). Fraction 9 (4.5 g) was separated by pHPLC [CH_3_CN-1% HAc (16:84, v/v)], and eurylosesquiterpenoside B (**2**, 42.5 mg), eurylosesquiterpenoside C (**3**, 20.3 mg), eurylosesquiterpenoside D (**4**, 7.0 mg), massonside B (**12**, 31.6 mg), massonside A (**13**, 15.8 mg) were obtained. Fraction 10 (11.0 g) was subjected to ODS CC [MeOH-H_2_O (10:90 → 20:80 → 30:70 → 40:60 → 50:50 → 60:40 → 100:0, v/v)] to gain Fr. 10-1–Fr. 10-9. Fraction 10-6 (581.1 mg) was purified by pHPLC [MeOH-1% HAc (25:75, v/v)] and [CH_3_CN-1% HAc (11:89, v/v)], successively, and eurylosesquiterpenoside A (**1**, 11.6 mg) was produced.

EtOAc layer extract (150.0 g) was separated by silica gel CC [PE-CH_2_Cl_2_ (8:1 → 3:1 → 0:100, v/v) → CH_2_Cl_2_-MeOH (100:1 → 100:3 → 100:7 → 10:1 → 0:100, v/v)] to give Fr. E-1–Fr. E-15. Fraction E-8 (9.8 g) was purified by Sephadex LH-20 CC (MeOH) to yield Fr. E-8-1–Fr. E-8-4. Fraction E-8-3 (5.3 g) was fractionated over ODS CC [MeOH-H_2_O (50:50 →60:40 → 70:30 → 80:20 → 90:10 → 100:0, v/v)], and Fr. E-8-3-1-Fr. E-8-3-16 were given. Fraction E-8-3-12 (286.7 mg) was separated by pHPLC [CH_3_CN-1% HAc (50:50, v/v)] to produce eurylosesquiterpenol E (**5**, 66.1 mg) and stachytriol (**19**, 58.0 mg). Fraction E-9 (15.5 g) was subjected to Sephadex LH-20 CC (MeOH) to yield Fr. E-9-1–Fr. E-9-4. Fraction E-9-3 (3.3 g) was further prepared by ODS CC [MeOH-H_2_O (50:50 →60:40 → 70:30 → 80:20 → 90:10 → 100:0, v/v)], then, Fr. E-9-3-1–Fr. E-9-3-12 were provided. Fraction E-9-3-4 (359.0 mg) was purified by pHPLC [CH_3_CN-1% HAc (30:70, v/v)] to gain 7-*epi*-11-hydroxychabrolidione A (**16**, 46.7 mg). Fraction E-9-3-8 (349.4 mg) was separated by pHPLC [CH_3_CN-1% HAc (55:45, v/v)] to yield oplodiol (**10**, 69.0 mg), eurylosesquiterpenol H (**8**, 15.1 mg), along with eurylosesquiterpenol F (**6**, 8.5 mg). Fraction E-10 (7.8 g) was subjected to Sephadex LH-20 CC (MeOH), and Fr. E-10-1–Fr. E-10-3 were given. Fraction E-10-3 (3.9 g) was fractionated over ODS CC [MeOH-H_2_O (50:50 →60:40 → 70:30 → 80:20 → 90:10 → 100:0, v/v)], and Fr. E-10-3-1–Fr. E-10-3-14 were furnished. Fraction E-10-3-10 (369.1 mg) was purified by pHPLC [CH_3_CN-1% HAc (40:60, v/v)] to produce (–)-4*α*,7*β*-aromaden-dranediol (**17**, 52.2 mg). Fraction E-10-3-12 (135.1 mg) was separated by pHPLC [CH_3_CN-1% HAc (45:55, v/v)] to give eurylosesquiterpenol I (**9**, 14.4 mg) and eurylosesquiterpenol G (**7**, 11.3 mg). Fraction E-11 (18.7 g) was chromatographed on Sephadex LH-20 CC eluted with MeOH to furnish Fr. E-11-1–Fr. E-11-3. Fraction E-11-2 (4.6 g) was subjected to ODS CC [MeOH-H_2_O (40:60 → 50:50 → 60:40 → 70:30 → 80:20 → 90:10 → 100:0, v/v)], and Fr. E-11-2-1–Fr. E-11-2-14 were yielded. Fraction E-11-2-6 (189.5 mg) was separated by pHPLC [CH_3_CN-1% HAc (27:73, v/v)] to give cadinane-4*β*,5*α*,10*β*-triol (**15**, 150.4 mg). Fraction E-11-2-12 (279.5 mg) was prepared with pHPLC [CH_3_CN-1% HAc (43:57, v/v)] to produce aromadendrane-4*α*,10*α*-diol (**18**, 13.2 mg). Fraction E-14 (4.3 g) was fractionated over Sephadex LH-20 CC (MeOH) to gain Fr. E-14–1-Fr. E-14–3. Fraction E-14–3 (123.4 mg) was purified by pHPLC [CH_3_CN-1% HAc (40:60, v/v)] to furnish 1(*R*),4*β*-dihydroxy*-trans*-eudesm-7-ene-1-*O*-*β*-d-glucopyranoside (**11**, 10.2 mg).

*Eurylosesquiterpenoside A* (**1**): White powder; (*α*)_D_^25^ –72.0 (*conc* 0.15, MeOH); IR *ν*_max_ (KBr) cm^−1^: 3,360, 2,936, 2,869, 1,575, 1,384, 1,073, 1,024, 912; ^1^H NMR (CD_3_OD, 500 MHz) *δ*_H_: 3.41 (1H, dd, *J* = 4.0, 11.5 Hz, H-1), 1.84 (1H, m, H*α*-2), 1.60 (1H, dt, *J* = 3.0, 14.0 Hz, H*β*-2), 1.47 (1H, dt, *J* = 3.5, 14.0 Hz, H*α*-3), 1.76 (1H, dt, *J* = 3.0, 14.0 Hz, H*β*-3), 1.27 (1H, dd, *J* = 2.0, 12.5 Hz, H-5), 1.93 (1H, br. d, *ca*. *J* = 15 Hz, H*α*-6), 1.12 (1H, m, overlapped, H*β*-6), 1.31 (1H, m, H-7), 1.63 (1H, dt, *J* = 3.0, 11.0 Hz, H*α*-8), 1.24 (1H, dt, *J* = 3.0, 13.0 Hz, H*β*-8), 1.13 (1H, m, overlapped, H*α*-9), 2.04 (1H, dt, *J* = 3.0, 13.0 Hz, H*β*-9), 1.16, 1.18, 0.91, 1.10 (3H each, all s, H_3_-12, 13, 14, 15), 4.29 (1H, d, *J* = 8.0 Hz, H-1′), 3.14 (1H, dd, *J* = 8.0, 8.5 Hz, H-2′), 3.35 (1H, dd, *J* = 8.5, 9.0 Hz, H-3′), 3.28 (1H, dd, *J* = 9.0, 9.5 Hz, H-4′), 3.23 (1H, ddd, *J* = 2.0, 5.5, 9.5 Hz, H-5′), [3.66 (1H, dd, *J* = 5.5, 11.5 Hz), 3.85 (1H, dd, *J* = 2.0, 11.5 Hz), H_2_-6′]; ^13^C NMR (CD_3_OD, 125 MHz) *δ*_C_: see Table 1; ESI-Q-Orbitrap MS *m/z* 463.25449 (M + COOH)^−^ (calcd for C_22_H_39_O_10_, 463.25377).

*Eurylosesquiterpenoside B* (**2**): White powder; (*α*)_D_^25^ –34.9 (*conc* 1.5, MeOH); IR *ν*_max_ (KBr) cm^−1^: 3,370, 2,958, 2,927, 2,870, 1,065, 1,024; ^1^H NMR (CD_3_OD, 500 MHz) *δ*_H_: 3.42 (1H, dd, *J* = 4.0, 11.5 Hz, H-1), 1.70 (1H, m, H*α*-2), 1.87 (1H, m, H*β*-2), 1.48 (1H, dt, *J* = 3.5, 13.5 Hz, H*α*-3), 1.75 (1H, dt, *J* = 2.5, 13.5 Hz, H*β*-3), 1.30 (1H, dd, *J* = 5.5, 12.0 Hz, H-5), 2.02 (1H, m, H*α*-6), 2.09 (1H, m, H*β*-6), 5.37 (1H, d, *J* = 5.0 Hz, H-8), 1.90 (1H, m, H*α*-9), 2.14 (1H, dd, *J* = 5.0, 17.5 Hz, H*β*-9), 2.22 (1H, sex like, *ca*. *J* = 7 Hz, H-11), [3.38 (1H, dd, *J* = 5.5, 10.5 Hz), 3.57 (1H, dd, *J* = 6.0, 10.5 Hz), H_2_-12], 1.04 (3H, d, *J* = 7.0 Hz, H_3_-13), 1.01, 1.14 (3H each, both s, H_3_-14, 15), 4.32 (1H, d, *J* = 7.5 Hz, H-1′), 3.16 (1H, dd, *J* = 7.5, 9.0 Hz, H-2′), 3.35 (1H, dd, *J* = 9.0, 9.0 Hz, H-3′), 3.27 (1H, dd, *J* = 9.0, 9.0 Hz, H-4′), 3.22 (1H, ddd, *J* = 2.0, 5.5, 9.0 Hz, H-5′), [3.66 (1H, dd, *J* = 5.5, 11.5 Hz), 3.85 (1H, dd, *J* = 2.0, 11.5 Hz), H_2_-6']; ^13^C NMR (CD_3_OD, 125 MHz) *δ*_C_: see Table 1; ESI-Q-Orbitrap MS *m/z* 461.23981 (M + COOH)^−^ (calcd for C_22_H_37_O_10_, 461.23812).

*Eurylosesquiterpenoside C* (**3**): White powder; (*α*)_D_^25^ –29.3 (*conc* 0.9, MeOH); IR *ν*_max_ (KBr) cm^−1^: 3,368, 2,958, 2,925, 2,877, 1,072, 1,021; ^1^H NMR (CD_3_OD, 500 MHz) *δ*_H_: 3.41 (1H, dd, *J* = 4.0, 12.0 Hz, H-1), 1.69 (1H, m, H*α*-2), 1.87 (1H, m, H*β*-2), 1.48 (1H, dt, *J* = 3.5, 13.5 Hz, H*α*-3), 1.75 (1H, dt, *J* = 3.0, 13.5 Hz, H*β*-3), 1.32 (1H, dd, *J* = 5.0, 12.0 Hz, H-5), 1.96 (1H, m, H*α*-6), 2.16 (1H, m, H*β*-6), 5.38 (1H, d, *J* = 5.5 Hz, H-8), 1.92 (1H, m, H*α*-9), 2.11 (1H, dd, *J* = 5.5, 17.0 Hz, H*β*-9), 2.23 (1H, sex, *J* = 7.0 Hz, H-11), 3.33, 3.57 (1H each, both dd, *J* = 7.0, 10.5 Hz, H_2_-12), 1.04 (3H, d, *J* = 7.0 Hz, H_3_-13), 1.00, 1.14 (3H each, both s, H_3_-14, 15), 4.32 (1H, d, *J* = 8.0 Hz, H-1′), 3.16 (1H, dd, *J* = 8.0, 9.0 Hz, H-2′), 3.35 (1H, dd, *J* = 8.5, 9.0 Hz, H-3′), 3.27 (1H, dd, *J* = 8.5, 9.0 Hz, H-4′), 3.22 (1H, ddd, *J* = 2.0, 6.0, 9.0 Hz, H-5′), [3.66 (1H, dd, *J* = 6.0, 12.0 Hz), 3.85 (1H, dd, *J* = 2.0, 12.0 Hz), H_2_-6′]; ^13^C NMR (CD_3_OD, 125 MHz) *δ*_C_: see Table 1; ESI-Q-Orbitrap MS *m/z* 461.23886 (M + COOH)^−^ (calcd for C_22_H_37_O_10_, 461.23812).

*Eurylosesquiterpenoside D* (**4**): White powder; (*α*)_D_^25^ –12.4 (*conc* 0.55, MeOH); CD (*conc* 0.002 M, MeOH) mdeg (*λ*_nm_): −1.04 (227), +0.71 (208), −4.69 (194); IR *ν*_max_ (KBr) cm^−1^: 3,356, 2,926, 2,877, 1,072, 1,024; ^1^H NMR (CD_3_OD, 500 MHz) *δ*_H_: 3.44 (1H, dd, *J* = 4.0, 11.5 Hz, H-1), 1.75 (1H, m, overlapped, H*α*-2), 1.95 (1H, m, H*β*-2), 1.48 (1H, dt, *J* = 4.0, 13.5 Hz, H*α*-3), 1.74 (1H, m, overlapped, H*β*-3), 1.87 (1H, m, H-5), 5.56 (1H, br. s, H-6), 1.93 (1H, m, H*α*-8), 1.99 (1H, m, H*β*-8), 1.26 (1H, m, H*α*-9), 2.05 (1H, m, H*β*-9), 2.28 (1H, sex like, *ca*. *J* = 7 Hz, H-11), [3.42 (1H, dd, *J* = 6.5, 10.0 Hz), 3.55 (1H, dd, *J* = 7.5, 10.0 Hz), H_2_-12], 1.03 (3H, d, *J* = 7.0 Hz, H_3_-13), 1.01, 1.20 (3H each, both s, H_3_-14, 15), 4.31 (1H, d, *J* = 7.5 Hz, H-1′), 3.17 (1H, dd, *J* = 7.5, 9.0 Hz, H-2′), 3.35 (1H, dd, *J* = 8.5, 9.0 Hz, H-3′), 3.28 (1H, dd, *J* = 8.5, 8.5 Hz, H-4′), 3.22 (1H, ddd, *J* = 2.0, 5.5, 8.5 Hz, H-5′), [3.65 (1H, dd, *J* = 5.5, 11.5 Hz), 3.85 (1H, dd, *J* = 2.0, 11.5 Hz), H_2_-6′]; ^13^C NMR (CD_3_OD, 125 MHz) *δ*_C_: see Table 1; ESI-Q-Orbitrap MS *m/z* 461.23914 (M + COOH)^−^ (calcd for C_22_H_37_O_10_, 461.23812).

*Eurylosesquiterpenol E* (**5**): White powder; (*α*)_D_^25^ –71.8 (*conc* 1.0, MeOH); UV *λ*_max_ (MeOH) nm (log *ε*): 241 (3.93); CD (*conc* 0.001 M, CH_3_CN) mdeg (*λ*_nm_): +3.26 (339), −31.10 (240), +52.31 (206); IR *ν*_max_ (KBr) cm^−1^: 3,473, 2,959, 2,935, 2,873, 1,664, 1,452, 1,389, 1,000, 879; ^1^H NMR (CDCl_3_, 500 MHz) *δ*_H_: 1.72 (1H, ddd, *J* = 3.0, 10.5, 14.0 Hz, H-1), 2.41 (1H, dd, *J* = 14.0, 16.5 Hz, H*α*-2), 2.60 (1H, dd, *J* = 3.0, 16.5 Hz, H*β*-2), 6.89 (1H, br. s, H-5), 2.40 (1H, m, H-6), 1.16 (1H, m, H-7), 1.52 (2H, m, H_2_-8), 1.77 (1H, m, H*α*-9), 1.45 (1H, m, H*β*-9), 2.25 (1H, m, H-11), 0.85, 0.98 (3H each, both d, *J* = 7.0 Hz, H_3_-12, 13), 1.19 (3H, s, H_3_-14), 1.78 (3H, br. s, H_3_-15); ^1^H NMR (C_5_D_5_N, 500 MHz) *δ*_H_: 1.70 (1H, ddd, *J* = 4.0, 10.5, 14.0 Hz, H-1), 2.79 (1H, dd, *J* = 14.0, 16.5 Hz, H*α*-2), 2.84 (1H, dd, *J* = 4.0, 16.5 Hz, H*β*-2), 6.93 (1H, br. s, H-5), 2.68 (1H, m, H-6), 1.13 (1H, tt, *J* = 3.5, 12.0 Hz, H-7), 1.81 (1H, dq, *J* = 3.5, 13.5 Hz, H*α*-8), 1.47 (1H, dq, *J* = 3.5, 13.5 Hz, H*β*-8), 1.90 (1H, dt, *J* = 3.5, 13.5 Hz, H*α*-9), 1.41 (1H, dt, *J* = 3.5, 13.5 Hz, H*β*-9), 2.20 (1H, m, H-11), 0.82, 0.95 (3H each, both d, *J* = 7.0 Hz, H_3_-12, 13), 1.27 (3H, s, H_3_-14), 1.93 (3H, br. s, H_3_-15); ^13^C NMR (CDCl_3_ and C_5_D_5_N, 125 MHz) *δ*_C_: see Table 2; ESI-Q-Orbitrap MS *m/z* 281.17557 (M + COOH)^−^ (calcd for C_16_H_25_O_4_, 281.17474).

*Eurylosesquiterpenol F* (**6**): White powder; (*α*)_D_^25^ –36.0 (*conc* 0.35, MeOH); CD (*conc* 0.001 M, CH_3_CN) mdeg (*λ*_nm_): +3.26 (339), −31.10 (240), +52.31 (206); UV *λ*_max_ (MeOH) nm (log *ε*): 241 (3.93); IR *ν*_max_ (KBr) cm^−1^: 3,428, 2,954, 2,931, 2,870, 1,628, 1,454, 1,374, 1,118, 879; ^1^H NMR (CDCl_3_, 500 MHz) *δ*_H_: 1.84 (1H, m, H-1), 2.12 (1H, dd, *J* = 14.0, 16.0 Hz, H*α*-2), 2.77 (1H, dd, *J* = 3.0, 16.0 Hz, H*β*-2), 6.80 (1H, br. s, H-5), 2.08 (1H, m, H-6), 1.21 (1H, m, H-7), 1.69 (1H, m, H*α*-8), 1.22 (1H, m, H*β*-8), 1.86 (1H, m, H*α*-9), 1.46 (1H, dt, *J* = 3.5, 13.0 Hz, H*β*-9), 2.23 (1H, m, H-11), 0.83, 0.99 (3H each, both d, *J* = 7.0 Hz, H_3_-12, 13), 1.17 (3H, s, H_3_-14), 1.79 (3H, br. s, H_3_-15); ^1^H NMR (C_5_D_5_N, 500 MHz) *δ*_H_: 2.09 (1H, m, H-1), 2.30 (1H, dd, *J* = 16.0, 16.0 Hz, H*α*-2), 2.28 (1H, dd, *J* = 2.0, 16.0 Hz, H*β*-2), 6.81 (1H, br. s, H-5), 2.11 (1H, m, H-6), 1.13 (1H, m, H-7), 1.55 (1H, dq, *J* = 3.5, 13.0 Hz, H*α*-8), 1.19 (1H, dq, *J* = 3.5, 13.0 Hz, H*β*-8), 2.00 (1H, dt, *J* = 3.5, 13.0 Hz, H*α*-9), 1.72 (1H, dt, *J* = 3.5, 13.0 Hz, H*β*-9), 2.14 (1H, m, H-11), 0.81, 0.92 (3H each, both d, *J* = 7.0 Hz, H_3_-12, 13), 1.29 (3H, s, H_3_-14), 1.92 (3H, br. s, H_3_-15); ^13^C NMR (CDCl_3_ and C_5_D_5_N, 125 MHz) *δ*_C_: see Table 2; ESI-Q-Orbitrap MS *m/z* 281.17548 (M + COOH)^−^ (calcd for C_16_H_25_O_4_, 281.17474).

*Eurylosesquiterpenol G.* (**7**): White powder; (*α*)_D_^25^ + 1.7 (*conc* 0.35, MeOH); CD (*conc* 0.001 M, CH_3_CN) mdeg (*λ*_nm_): −3.07 (240), −4.05 (198); IR *ν*_max_ (KBr) cm^−1^: 3,396, 2,958, 2,933, 2,870, 1715, 1,560, 1,454, 1,373, 1,024, 899; ^1^H NMR (CDCl_3_, 500 MHz) *δ*_H_: 1.31 (1H, m, H-1), 2.01 (1H, dt, *J* = 4.0, 13.0 Hz, H*α*-2), 1.55 (1H, dt, *J* = 4.0, 13.0 Hz, H*β*-2), 4.01 (1H, br. s, H-3), 5.74 (1H, br. s, H-5), 1.92 (1H, t like, *ca*. *J* = 11 Hz, H-6), 1.06 (1H, tt, *J* = 4.0, 12.0 Hz, H-7), 1.49 (1H, m, H*α*-8), 1.37 (1H, dq, *J* = 4.0, 12.0 Hz, H*β*-8), 1.75 (1H, m, H*α*-9), 1.45 (1H, dt, *J* = 4.0, 12.0 Hz, H*β*-9), 2.19 (1H, m, H-11), 0.80, 0.93 (3H each, both d, *J* = 7.0 Hz, H_3_-12, 13), 1.24 (3H, s, H_3_-14), 1.81 (3H, br. s, H_3_-15); ^1^H NMR (C_5_D_5_N, 500 MHz) *δ*_H_: 1.83 (1H, ddd, *J* = 3.0, 10.5, 13.0 Hz, H-1), 2.06 (1H, dt, *J* = 3.0, 13.0 Hz, H*α*-2), 2.49 (1H, dt, *J* = 3.0, 13.0 Hz, H*β*-2), 4.34 (1H, br. s, H-3), 5.92 (1H, br. s, H-5), 2.46 (1H, dd like, *ca*. *J* = 11, 11 Hz, H-6), 1.10 (1H, m, H-7), 1.48 (2H, m, H_2_-8), 1.99 (2H, m, H_2_-9), 2.25 (1H, m, H-11), 0.86, 0.92 (3H each, both d, *J* = 7.0 Hz, H_3_-12, 13), 1.45 (3H, s, H_3_-14), 2.08 (3H, br. s, H_3_-15); ^13^C NMR (CDCl_3_ and C_5_D_5_N, 125 MHz) *δ*_C_: Table 2; ESI-Q-Orbitrap MS *m/z* 283.19107 (M + COOH)^−^ (calcd for C_16_H_27_O_4_, 283.19039).

*Eurylosesquiterpenol H* (**8**): White powder; (*α*)_D_^25^ −4.7 (*conc* 0.65, MeOH); IR *ν*_max_ (KBr) cm^−1^: 3,393, 2,954, 2,927, 2,870, 1,454, 1,375, 1,094, 1,024; ^1^H NMR (CDCl_3_, 500 MHz) *δ*_H_: 4.70 (1H, br. d, *ca*. *J* = 9 Hz, H-2), 1.60 (1H, m, overlapped, H*α*-3), 1.83 (1H, m, H*β*-3), 2.69 (1H, m, H-4), 2.03 (1H, m, H-5), 1.43 (1H, m, H*α*-6), 1.47 (1H, dd like, *ca*. *J* = 15, 15 Hz, H*β*-6), 1.68 (1H, m, overlapped, H-7), 1.91 (1H, m, H*α*-8), 1.68 (1H, m, overlapped, H*β*-8), 2.14 (1H, t like, *ca*. *J* = 13 Hz, H*α*-9), 1.61 (1H, m, overlapped, H*β*-9), 1.17, 1.30, 1.10 (3H each, all s, H_3_-12, 13, 14), 0.89 (3H, d, *J* = 7.0 Hz, H_3_-15); ^1^H NMR (C_5_D_5_N, 500 MHz) *δ*_H_: 5.05 (1H, d, *J* = 6.0 Hz, H-2), 1.95 (2H, m, H_2_-3), 2.99 (1H, m, H-4), 2.25 (1H, m, H-5), 1.38 (1H, dd like, *ca*. *J* = 8, 14 Hz, H*α*-6), 1.54 (1H, dd like, *ca*. *J* = 14, 14 Hz, H*β*-6), 1.61 (1H, m, H-7), 1.88 (1H, m, H*α*-8), 1.70 (1H, m, H*β*-8), 2.43 (1H, ddd, *J* = 5.0, 13.0, 18.0 Hz, H*α*-9), 1.67 (1H, m, H*β*-9), 1.20, 1.31, 1.44 (3H each, all s, H_3_-12, 13, 14), 0.91 (3H, d, *J* = 7.5 Hz, H_3_-15); ^13^C NMR (CDCl_3_ and C_5_D_5_N, 125 MHz) *δ*_C_: see Table 2; ESI-Q-Orbitrap MS *m/z* 317.19626 (M + COOH)^−^ (calcd for C_16_H_29_O_6_, 317.19587).

*Eurylosesquiterpenol I* (**9**): White powder; (*α*)_D_^25^ −3.2 (*conc* 0.25, MeOH); IR*ν*_max_ (KBr) cm^−1^: 3,395, 2,926, 2,861, 1704, 1,455, 1,377; ^1^H NMR (CDCl_3_, 500 MHz) *δ*_H_: 1.92 (1H, m, H-1), 1.76 (1H, m, overlapped, H*α*-2), 1.43 (1H, m, overlapped, H*β*-2), 1.59 (1H, m, overlapped, H*α*-3), 1.74 (1H, m, overlapped, H*β*-3), 1.43 (1H, m, overlapped, H-5), 0.47 (1H, dd, *J* = 10.0, 10.5 Hz, H-6), 0.69 (1H, ddd, *J* = 5.5, 10.0, 15.0 Hz, H-7), 1.99 (1H, m, overlapped, H*α*-8), 1.12 (1H, m, overlapped, H*β*-8), 1.97 (1H, m, overlapped, H*α*-9), 1.58 (1H, m, overlapped, H*β*-9), 2.20 (1H, dt, *J* = 3.0, 11.0 Hz, H-10), 1.06, 1.09, 1.26 (3H each, all s, H_3_-12, 13, 15); ^1^H NMR (C_5_D_5_N, 500 MHz) *δ*_H_: 2.29 (1H, m, H-1), 2.10 (1H, m, H*α*-2), 1.93 (1H, m, H*β*-2), 1.70 (1H, ddd, *J* = 6.0, 8.0, 15.5 Hz, H*α*-3), 2.05 (1H, ddd, *J* = 4.5, 8.0, 15.5 Hz, H*β*-3), 1.83 (1H, dd, *J* = 10.0, 10.5 Hz, H-5), 0.56 (1H, dd, *J* = 10.0, 10.5 Hz, H-6), 0.67 (1H, ddd, *J* = 6.5, 10.0, 15.5 Hz, H-7), 1.96 (1H, m, H*α*-8), 1.23 (1H, m, H*β*-8), 2.22 (1H, m, H*α*-9), 1.91 (1H, m, H*β*-9), 2.53 (1H, dt, *J* = 3.0, 10.5 Hz, H-10), 1.08, 1.25, 1.45 (3H each, all s, H_3_-12, 13, 15); ^13^C NMR (CDCl_3_ and C_5_D_5_N, 125 MHz) *δ*_C_: see Table 2; ESI-Q-Orbitrap MS *m/z* 251.16508 (M—H)^−^ (calcd for C_15_H_23_O_3_, 251.16417).

#### Acid Hydrolysis of 1–4

A solution of compounds **1**–**4** (1.0 mg each) in 1 M HCl (1 ml) was heated under reflux for 3 h, the reaction product was extract with EtOAc. The aqueous layer was analyzed by using HPLC [column: Kaseisorb LC NH_2_-60-5, 4.6 mm i. d. × 250 mm (Tokyo Kasei Co., Ltd, Tokyo, Japan); mobile phase: CH_3_CN-H_2_O (80:20, v/v); flow rate: 0.8 ml/min]. As results, d-glucose was identified from **1**–**4** by comparing their retention time and optical rotation with that of authentic d-glucose (*t*_R_: 10.4 min, positive optical rotation).

#### Computations

Relative configurations of compounds **4**–**7** were deduced by analyses of their 1D and 2D NMR data assisted by Chem3D modeling. Conformation search was then firstly accomplished under the MMFF94 force field by using CONFLEX 8 software (Takanawa, 2019), and the low energy conformers, which meet the requirements of NOESY analysis, were selected out for further computations. To verify the stabilities of the selected conformers, geometry optimizations and the frequencies pre-calculations were finished by DFT method at the APFD/6-311+G(2d,p) basis set level in methanol (for **4**) or acetonitrile (for **5**–**7**), using Gaussian 16 package (Revision C.01) (Frisch et al., 2019). By TD-SCF/DFT method, energies of one hundred excitation states of the optimized conformers were then calculated at the APFD/6-311+G(2d,p) level with a IEFPCM solvent model in MeOH or acetonitrile. With a half bandwidth of ∼0.2 eV, the calculation results were Boltzmann averaged to simulate the ECD spectra after UV correction, which were finally extracted by GaussView 6.0 and Origin Pro 2016 software before comparing with those experimental data.

### Experimental Procedures for Bioassay

#### Materials

Cell viability was measured on BioTek Cytation five-cell imaging multi-mode reader (Winooski, VT, United States); Light damage model and radiation dose were tested on UVB radiation machine (SH4B, Sigma, Shanghai, China) and UVB radiometer (ST90-UVB, 297 nm, Beijing, China), respectively. Dry thermostat (Hangzhou Allsheng Instrusment Co., Itd. Hangzhou, China), western blot electrophoresis and membrane transfer instrument (Bio Rad, United States), and Amersham imager 600 gel imaging system (GE Healthcare, United States) were used to western blot assay.

HaCaT cell lines were gained from Procell Life Science & Technology Co., Ltd. (Wuhan, China); Fetal bovine serum (FBS) was obtained from Biological Industries (Beit-Haemek, Israel); Minimum essential medium (MEM) was ordered from Corning (Shanghai, China); Penicillin and streptomycin were purchased from Thermo Fisher Scientific (Waltham, MA, United States); MTT and dimethyl sulfoxide (DMSO) were gained from Sigma-Aldrich (St. Louis, MO, United States); Vitamin C (Vc) were purchased from Shanghai Yuanye Bio-Technology Co., Ltd. (Shanghai, China); BCA protein quantification kit was ordered from Thermo Fisher Scientific (Waltham, United States); PVDF membrane was purchased from Merch/Millipore (Schwalbach, Germany); Immobilon western chemilumescent HRP substrate was gained from Millipore (Massachusetts, United States); TNF-*α* (ab6671), COX-2 (ab52237), *β*-actin (ab8227) JNK (ab208035), and p-JNK (ab4821) were ordered from abcam (Cambs, United Kingdom); p38 (8690S), p-p38 (4511S), ERK (4695S), p-ERK (4370S), and COL1A1 (72026S) were purchased from CST (Massachusetts, United States); MMP-1 (SC-137044) was obtained from Santa Cruz Biotech.INC. (United States).

#### Cell Culture

HaCaT cells were maintained in MEM medium with 10% FBS, streptomycin (100 μg/ml), penicillin (100 U/mL), and incubated at 5% CO_2_, 37°C. When the cells grew to 80% confluence, they were seeded in 96-well plates at 1 × 10^4^ cells/well, and then processed the treatment.

#### Cell Viability Assay

MTT assay was applied to test cell viability. HaCaT cells were incubated at 96-well plates and treated with or without test samples for 24 h, respectively. The culture condition was similar to “*Cell Culture*.” The medium was removed, then 1% MTT (5 mg/ml) were added into wells to format formazan. After incubating 4 h, the supernatant was removed, then 100 μL dimethyl sulfoxide (DMSO) was added in each well to dissolve the formazan. The absorbance was measured with a microplate reader at 490 nm.

#### Selection of Ultraviolet B Radiation Dose

After being cultured with MEM medium containing 10% FBS, streptomycin (100 μg/ml), penicillin (100 U/ml) in 96-well plates until grown to 70% confluence, the HaCaT cells were covered with fresh medium for 24 h. Then, the fresh medium was replaced with 100 μL/well PBS, and the cells were exposed to 50, 75, 100, 125, and 150 mJ/cm^2^ of UVB, respectively. After irradiation, 100 μL/well PBS was removed, and the cells were cultured with 100 μL/well fresh medium for 24 h again. The cell viability was tested in line with “*Cell Viability Assay*.”

#### Cell Viabilities of Ultraviolet B Induced HaCaT Cells Pretreated With Compounds

HaCaT cells were seeded in 96-well culture plates with complete medium until grown to 70% confluence, and then treated with fresh medium containing various concentrations of samples (10, 25, and 50 μM) for 24 h. Then, the cells were irradiated with UVB at 125 mJ/cm^2^ (UVB-irradiated with 0.46 mW cm^−2^ s^−1^ for approximately 272 s) in 100 μL PBS. After irradiation, the PBS was immediately replaced by 100 μL fresh medium and incubated for 24 h. Finally, the cell viability was measured by using the same method as that described in the part of “*Cell Viability Assay*.”

#### Western Blot Assay

Protein isolation and western blot assay were performed as previously described (Han et al., 2021). Briefly, protein was subjected to SDS-PAGE with 10% or 15% resolving gel, then the proteins on gels were separated, and electrotransferred onto PVDF membranes. Which were incubated at 4°C overnight with primary antibodies against rabbit polyclonal to TNF-*α* (1:500), COX-2 (1:1,000), *β*-actin (1:1,000), p38 (1:1,000), p-p38 (1:1,000), ERK (1:1,000), p-ERK (1:1,000), JNK (1:1,000), p-JNK (1:1,000), COL1A1 (1:1,000); and mouse polyclonal to MMP-1 (1:500). After washing with PBST, the PVDF membranes were incubated with a horseradish peroxidase-labeled secondary goat anti-rabbit (1:10,000) antibody (Abcam) or horseradish peroxidase-labeled secondary goat anti-mouse (1:10,000) antibody (Zhongshan Goldbridge Biotechnolog) for 1 h at room temperature, and washed again. Eventually, PVDF membranes were incubated with immobilon western chemilumescent HRP substrate and then scanned with ChemiDoc MP Imaging System to form images. The protein bands were analyzed with the ImageJ software (Version 1.0, National Institutes of Health, Bethesda, MD, United States). The treatment groups were normalized to Nor. Three independent assays were necessary.

#### Statistical Analysis

All experimental results were presented as the means ± standard error of mean (SEM). SPSS 26.0 was used to conduct the statistics of all data. Unpaired Student’s t-test (when two groups were analyzed) and one-way analysis of variance (ANOVA) (for > 3 groups) were used to analyze results. *p* < 0.05 was considered to indicate a statistically significant difference.

## Conclusion

In summary, in the process of investigating photoprotective constituents from natural products, nine new sesquiterpenes, named as eurylosesquiterpenosides A–D (**1**–**4**), eurylosesquiterpenols E**–**I (**5**–**9**), together with ten known ones were obtained and identified from the 70% EtOH extract of *O. elatus* stems. Though the diverse ingredients such as volatile oil, phenolic acids, lignans, quinic acid esters, anthraquinones, steroids, and aliphatic compounds had been reported from the medicine (Yan et al., 2021), the sesquiterpenes were rarely found in it, which enriched its material base.

Furthermore, our study suggested that the underlying mechanism of active-sesquiterpenes might be relevance with down-regulating MMP-1 expression *via* the decreasing production of inflammatory mediators and cytokines in UVB-irradiated HaCaT cells.

## Data Availability

The original contributions presented in the study are included in the article/Supplementary Material, further inquiries can be directed to the corresponding authors.
