# Supplementary material for: Sesquiterpenes From Oplopanax elatus Stems and Their Anti-Photoaging Effects by Down-Regulating Matrix Metalloproteinase-1 Expression via Anti-Inflammation
Source: Front Chem. 2021 Nov 4;9:766041. doi: 10.3389/fchem.2021.766041 (PMC8600134; doi:10.3389/fchem.2021.766041)
Supplement: Supplementary file 5 [file Table1.DOC]

Supplementary Material

**Figure S1.** ^1^H NMR (500 MHz, CD_3_OD) spectrum of **1**.............................................................................................6

**Figure S2.** ^13^C NMR (125 MHz, CD_3_OD) spectrum of **1**............................................................................................6

**Figure S3.** ^1^H ^1^H COSY (CD_3_OD) spectrum of **1**........................................................................................................7

**Figure S4.** HSQC (CD_3_OD) spectrum of **1**..................................................................................................................7

**Figure S5.** HMBC (CD_3_OD) spectrum of **1**.................................................................................................................8

**Figure S6.** NOESY (CD_3_OD) spectrum of **1**................................................................................................................8

**Figure S7.** ESI-Q-Orbitrap-MS spectrum of **1**.............................................................................................................9

**Figure S8.** IR spectrum of **1**.........................................................................................................................................9

**Figure S9.** ^1^H NMR (500 MHz, CD_3_OD) spectrum of **2**...........................................................................................10

**Figure S10.** ^13^C NMR (125 MHz, CD_3_OD) spectrum of **2**........................................................................................10

**Figure S11.**^1^H ^1^H COSY (CD_3_OD) spectrum of **2**....................................................................................................11

**Figure S12.** HSQC (CD_3_OD) spectrum of **2**..............................................................................................................11

**Figure S13.** HMBC (CD_3_OD) spectrum of **2**.............................................................................................................12

**Figure S14.** NOESY (CD_3_OD) spectrum of **2**............................................................................................................12

**Figure S15.** ESI-Q-Orbitrap-MS spectrum of **2**.........................................................................................................13

**Figure S16.** IR spectrum of **2**.....................................................................................................................................13

**Figure S17.** ^1^H NMR (500 MHz, CD_3_OD) spectrum of **3**.........................................................................................14

**Figure S18.**^13^C NMR (125 MHz, CD_3_OD) spectrum of **3**........................................................................................14

**Figure S19.** ^1^H ^1^H COSY (CD_3_OD) spectrum of **3**....................................................................................................15

**Figure S20.** HSQC (CD_3_OD) spectrum of **3**..............................................................................................................15

**Figure S21.** HMBC (CD_3_OD) spectrum of **3**.............................................................................................................16

**Figure S22.** NOESY (CD_3_OD) spectrum of **3**............................................................................................................16

**Figure S23.** ESI-Q-Orbitrap-MS spectrum of **3**.........................................................................................................17

**Figure S24.** IR spectrum of **3**.....................................................................................................................................17

**Figure S25.** The HPLC analysis spectra of **2** and **3**................................................................................17

**Figure S26.** ^1^H NMR (500 MHz, CD_3_OD) spectrum of **4**.........................................................................................18

**Figure S27.** ^13^C NMR (125 MHz, CD_3_OD) spectrum of **4**........................................................................................18

**Figure S28.** ^1^H ^1^H COSY (CD_3_OD) spectrum of **4**....................................................................................................19

**Figure S29.** HSQC (CD_3_OD) spectrum of **4**..............................................................................................................19

**Figure S30.** HMBC (CD_3_OD) spectrum of **4**.............................................................................................................20

**Figure S31.** NOESY (CD_3_OD) spectrum of **4**............................................................................................................20

**Figure S32.** ESI-Q-Orbitrap-MS spectrum of **4**.........................................................................................................21

**Figure S33.** IR spectrum of **4**.....................................................................................................................................21

**Figure S34.** CD spectrum of **4**.....................................................................................................................................21

**Figure S35.** The conformational analysis and optimized geometries of predominant conformers of **4**.....................22

**Figure S36.** ^1^H NMR (500 MHz, C_5_D_5_N) spectrum of **5**...........................................................................................23

**Figure S37.** ^13^C NMR (125 MHz, C_5_D_5_N) spectrum of **5**..........................................................................................23

**Figure S38.** ^1^H ^1^H COSY (C_5_D_5_N) spectrum of **5**.....................................................................................................24

**Figure S39.** HSQC (C_5_D_5_N) spectrum of **5**................................................................................................................24

**Figure S40.** HMBC (C_5_D_5_N) spectrum of **5**...............................................................................................................25

**Figure S41.** ^1^H NMR (500 MHz, CDCl_3_) spectrum of **5**...........................................................................................25

**Figure S42.** ^13^C NMR (125 MHz, CDCl_3_) spectrum of **5**..........................................................................................26

**Figure S43.** ^1^H ^1^H COSY (CDCl_3_) spectrum of **5**......................................................................................................26

**Figure S44.** HSQC (CDCl_3_) spectrum of **5**................................................................................................................27

**Figure S45.** HMBC (CDCl_3_) spectrum of **5**...............................................................................................................27

**Figure S46.** NOESY (CDCl_3_) spectrum of **5**..............................................................................................................28

**Figure S47.** ESI-Q-Orbitrap-MS spectrum of **5**.........................................................................................................28

**Figure S48.** UV spectrum of **5**...................................................................................................................................29

**Figure S49.** IR spectrum of **5**.....................................................................................................................................29

**Figure S50.** CD spectrum of **5**.....................................................................................................................................29

**Figure S51.** The conformational analysis and optimized geometries of predominant conformers of **5**...................30

**Figure S52.** ^1^H NMR (500 MHz, C_5_D_5_N) spectrum of **6**...........................................................................................31

**Figure S53.** ^13^C NMR (125 MHz, C_5_D_5_N) spectrum of **6**..........................................................................................31

**Figure S54.** ^1^H ^1^H COSY (C_5_D_5_N) spectrum of **6**.....................................................................................................32

**Figure S55.** HSQC (C_5_D_5_N) spectrum of **6**................................................................................................................32

**Figure S56.** HMBC (C_5_D_5_N) spectrum of **6**...............................................................................................................33

**Figure S57.** ^1^H NMR (500 MHz, CDCl_3_) spectrum of **6**...........................................................................................33

**Figure S58.** ^13^C NMR (125 MHz, CDCl_3_) spectrum of **6**..........................................................................................34

**Figure S59.** ^1^H ^1^H COSY (CDCl_3_) spectrum of **6**......................................................................................................34

**Figure S60.** HSQC (CDCl_3_) spectrum of **6**................................................................................................................35

**Figure S61.** HMBC (CDCl_3_) spectrum of **6**...............................................................................................................35

**Figure S62.** NOESY (CDCl_3_) spectrum of **6**..............................................................................................................36

**Figure S63.** ESI-Q-Orbitrap-MS spectrum of **6**.........................................................................................................36

**Figure S64.** UV spectrum of **6**..................................................................................................................................37

**Figure S65.** IR spectrum of **6**..................................................................................................................................37

**Figure S66.** CD spectrum of **6**.....................................................................................................................................37

**Figure S67.** The conformational analysis and optimized geometries of predominant conformers of **6**.....................38

**Figure S68.** ^1^H NMR (500 MHz, C_5_D_5_N) spectrum of **7**...........................................................................................39

**Figure S69.** ^13^C NMR (125 MHz, C_5_D_5_N) spectrum of **7**..........................................................................................39

**Figure S70.** ^1^H ^1^H COSY (C_5_D_5_N) spectrum of **7**.....................................................................................................40

**Figure S71.** HSQC (C_5_D_5_N) spectrum of **7**................................................................................................................40

**Figure S72.** HMBC (C_5_D_5_N) spectrum of **7**...............................................................................................................41

**Figure S73.** NOESY (C_5_D_5_N) spectrum of **7**.............................................................................................................41

**Figure S74.** ^1^H NMR (500 MHz, CDCl_3_) spectrum of **7**...........................................................................................42

**Figure S75.** ^13^C NMR (125 MHz, CDCl_3_) spectrum of **7**..........................................................................................42

**Figure S76.** ^1^H ^1^H COSY (CDCl_3_) spectrum of **7**......................................................................................................43

**Figure S77.** HSQC (CDCl_3_) spectrum of **7**................................................................................................................43

**Figure S78.** HMBC (CDCl_3_) spectrum of **7**...............................................................................................................44

**Figure S79.** ESI-Q-Orbitrap-MS spectrum of **7**.........................................................................................................44

**Figure S80.** CD spectrum of **7**.....................................................................................................................................45

**Figure S81.** IR spectrum of **7**....................................................................................................................................45

**Figure S82.** The conformational analysis and optimized geometries of predominant conformers of **7**....................45

**Figure S83.** ^1^H NMR (500 MHz, C_5_D_5_N) spectrum of **8**...........................................................................................46

**Figure S84.** ^13^C NMR (125 MHz, C_5_D_5_N) spectrum of **8**........................................................................................46

**Figure S85.** ^1^H ^1^H COSY (C_5_D_5_N) spectrum of **8**.....................................................................................................47

**Figure S86.** HSQC (C_5_D_5_N) spectrum of **8**................................................................................................................47

**Figure S87.** HMBC (C_5_D_5_N) spectrum of **8**...............................................................................................................48

**Figure S88.** ^1^H NMR (500 MHz, CDCl_3_) spectrum of **8**...........................................................................................48

**Figure S89.** ^13^C NMR (125 MHz, CDCl_3_) spectrum of **8**..........................................................................................49

**Figure S90.** ^1^H ^1^H COSY (CDCl_3_) spectrum of **8**......................................................................................................49

**Figure S91.** HSQC (CDCl_3_) spectrum of **8**................................................................................................................50

**Figure S92.** HMBC (CDCl_3_) spectrum of **8**...............................................................................................................50

**Figure S93.** NOESY (CDCl_3_) spectrum of **8**..............................................................................................................51

**Figure S94.** ESI-Q-Orbitrap-MS spectrum of **8**.........................................................................................................51

**Figure S95.** IR spectrum of **8**....................................................................................................................................52

**Figure S96.** ^1^H NMR (500 MHz, C_5_D_5_N) spectrum of **9**...........................................................................................53

**Figure S97.** ^13^C NMR (125 MHz, C_5_D_5_N) spectrum of **9**..........................................................................................53

**Figure S98.** ^1^H ^1^H COSY (C_5_D_5_N) spectrum of **9**.....................................................................................................54

**Figure S99.** HSQC (C_5_D_5_N) spectrum of **9**...............................................................................................................54

**Figure S100.** HMBC (C_5_D_5_N) spectrum of **9**..............................................................................................................55

**Figure S101.** NOESY (C_5_D_5_N) spectrum of **9**.........................................................................................................55

**Figure S102.** ^1^H NMR (500 MHz, CDCl_3_) spectrum of **9**........................................................................................56

**Figure S103.** ^13^C NMR (125 MHz, CDCl_3_) spectrum of **9**.......................................................................................56

**Figure S104.** ^1^H ^1^H COSY (CDCl_3_) spectrum of **9**..................................................................................................57

**Figure S105.** HSQC (CDCl_3_) spectrum of **9**.............................................................................................................57

**Figure S106.** HMBC (CDCl_3_) spectrum of **9**...........................................................................................................58

**Figure S107.** ESI-Q-Orbitrap-MS spectrum of **9**........................................................................................................58

**Figure S108.** IR spectrum of **9**................................................................................................................................59

**Figure S109.** The HPLC spectra of the acid hydrolysates of **1**–**4**...............................................................60

**Figure S110.** The toxicity of compounds **1**–**19** at the concentration of 50 *μ*M on HaCaT cells...............................61

**Figure S111.** The raw data of Figure 9 ....................................................................................................................62

**Figure S112.** The raw data of Figure 10 ...................................................................................................................63

**Figure S113.** The raw data of Figure 11 ...................................................................................................................64


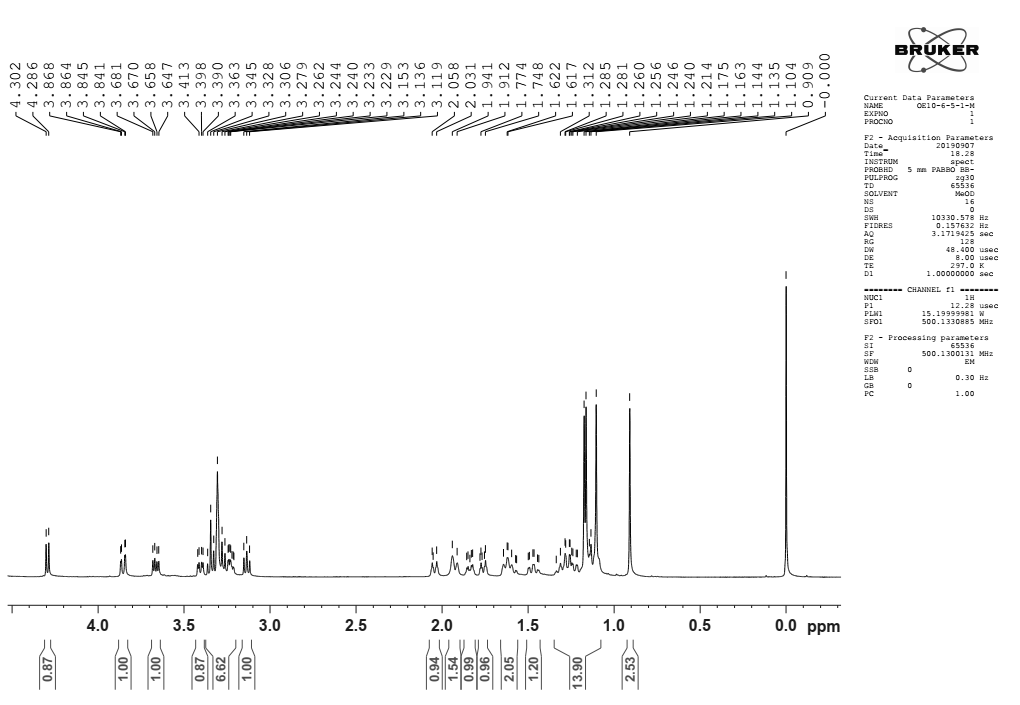


**Figure S1.** ^1^H NMR (500 MHz, CD_3_OD) spectrum of **1**


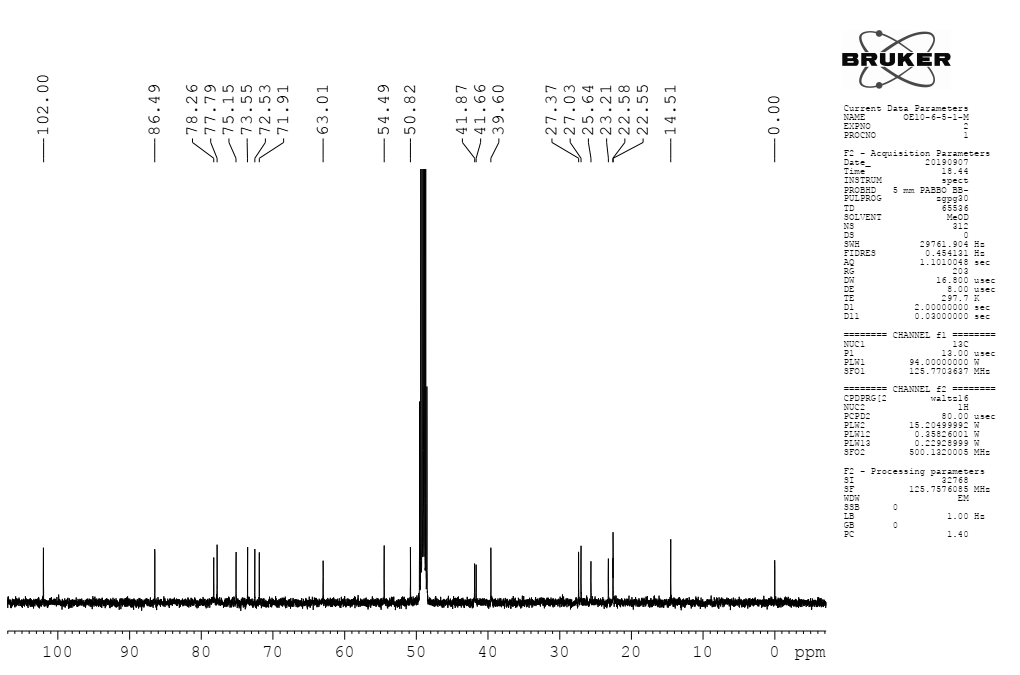


**Figure S2.** ^13^C NMR (125 MHz, CD_3_OD) spectrum of **1**


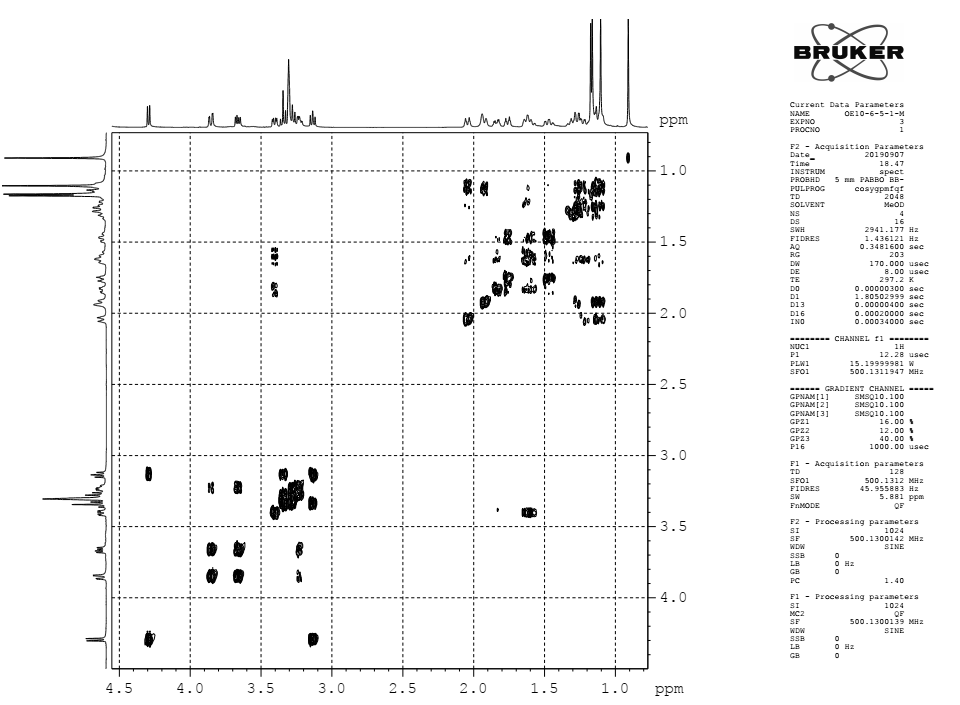


**Figure S3.** ^1^H ^1^H COSY (CD_3_OD) spectrum of **1**


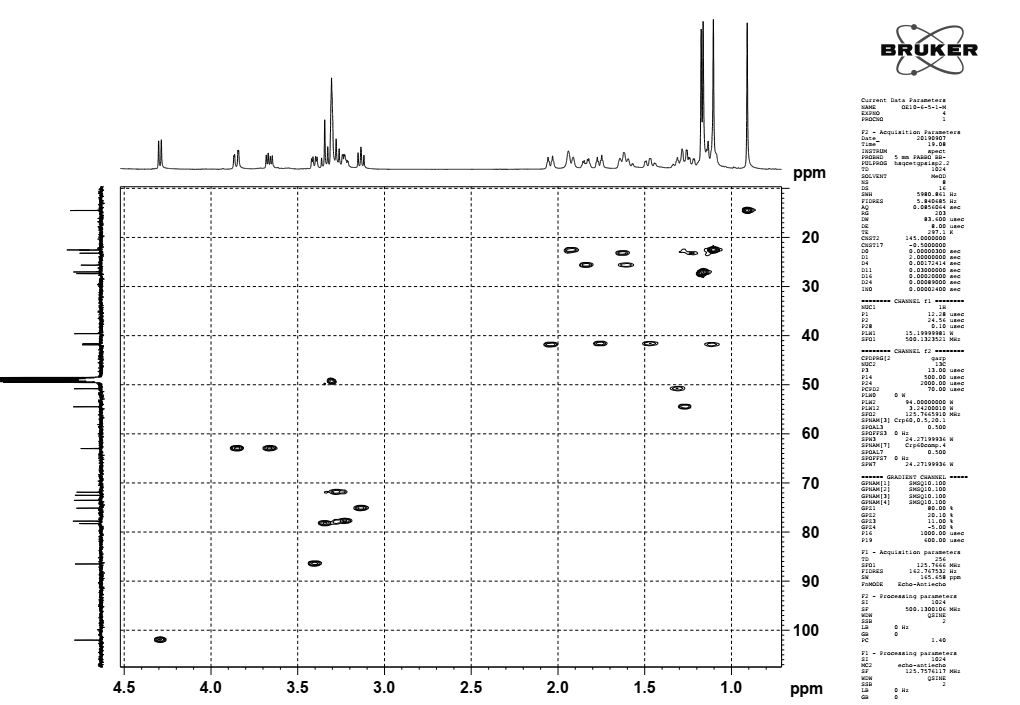


**Figure S4.** HSQC (CD_3_OD) spectrum of **1**


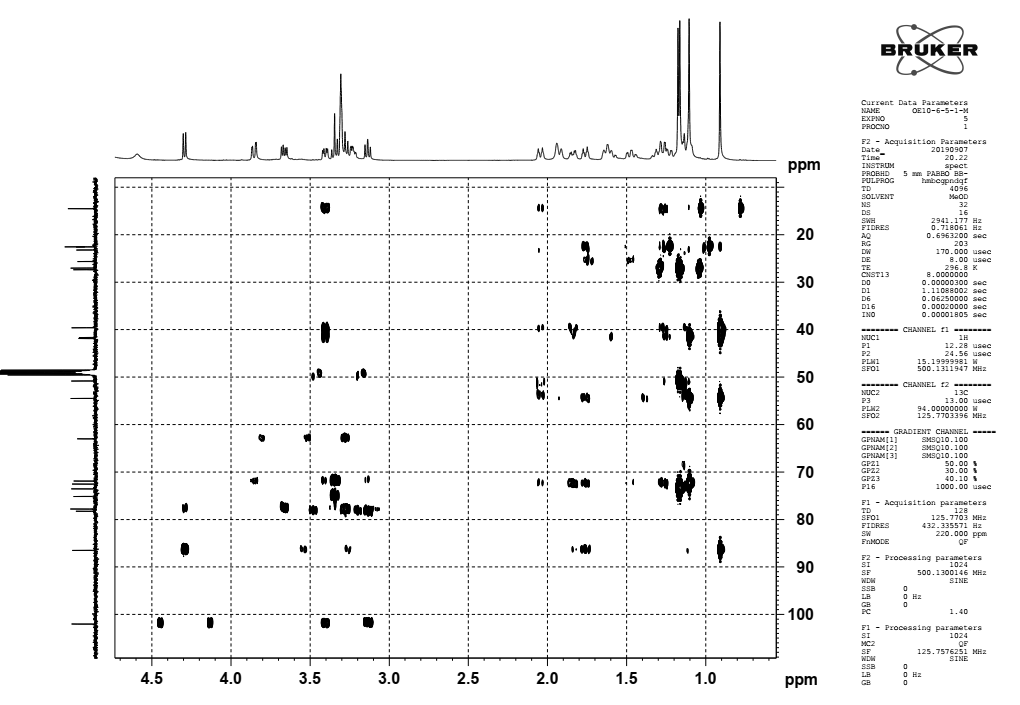


**Figure S5.** HMBC (CD_3_OD) spectrum of **1**


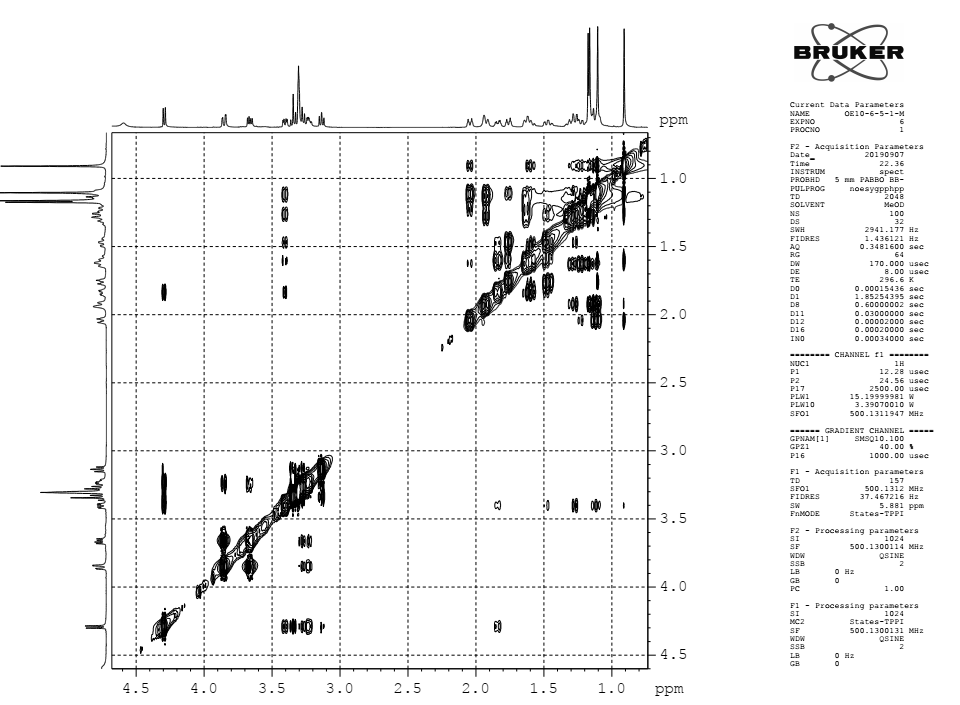


**Figure S6.** NOESY (CD_3_OD) spectrum of **1**


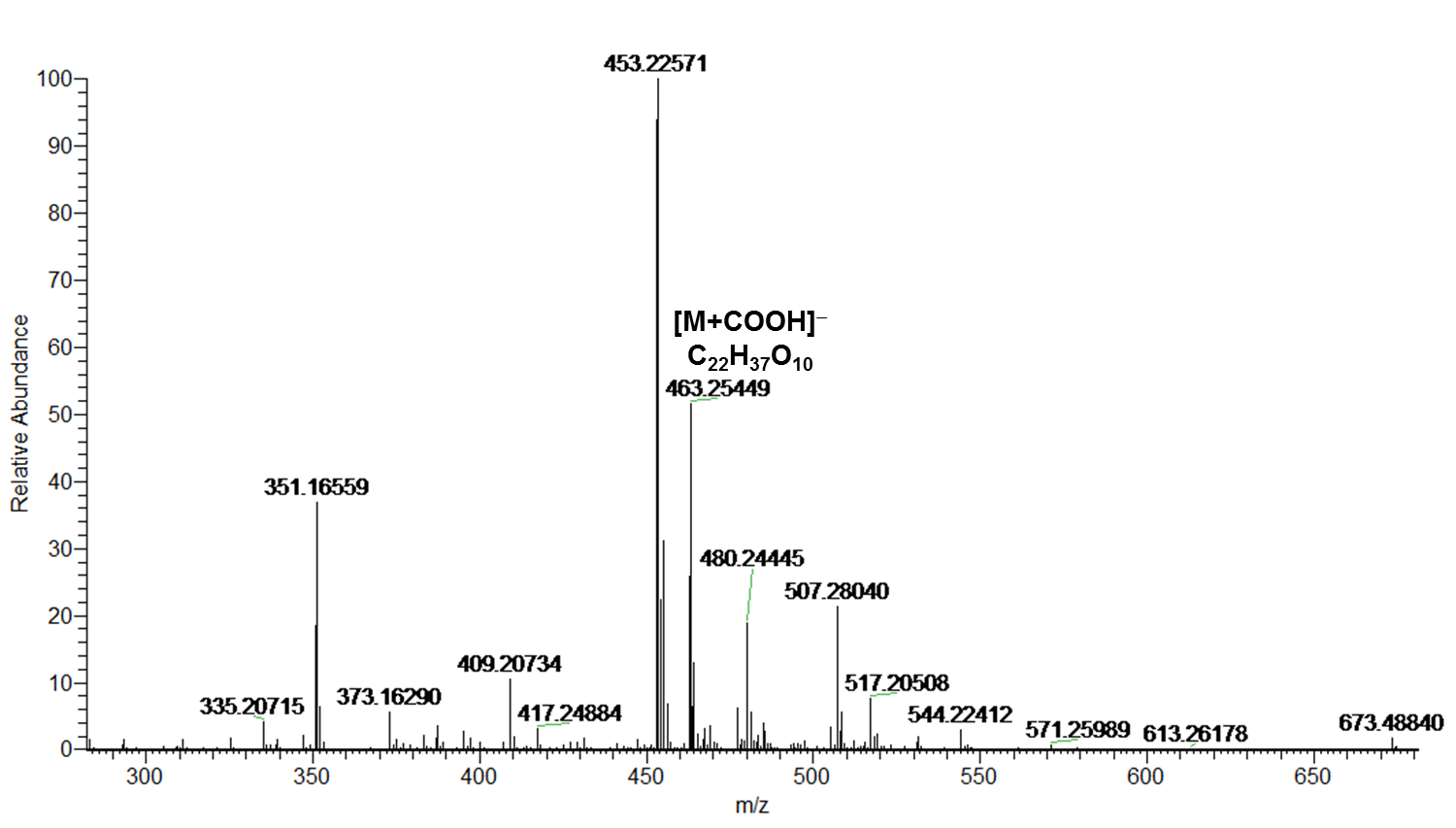


**Figure S7.** ESI-Q-Orbitrap-MS spectrum of **1**

**Figure S8.** IR spectrum of **1**


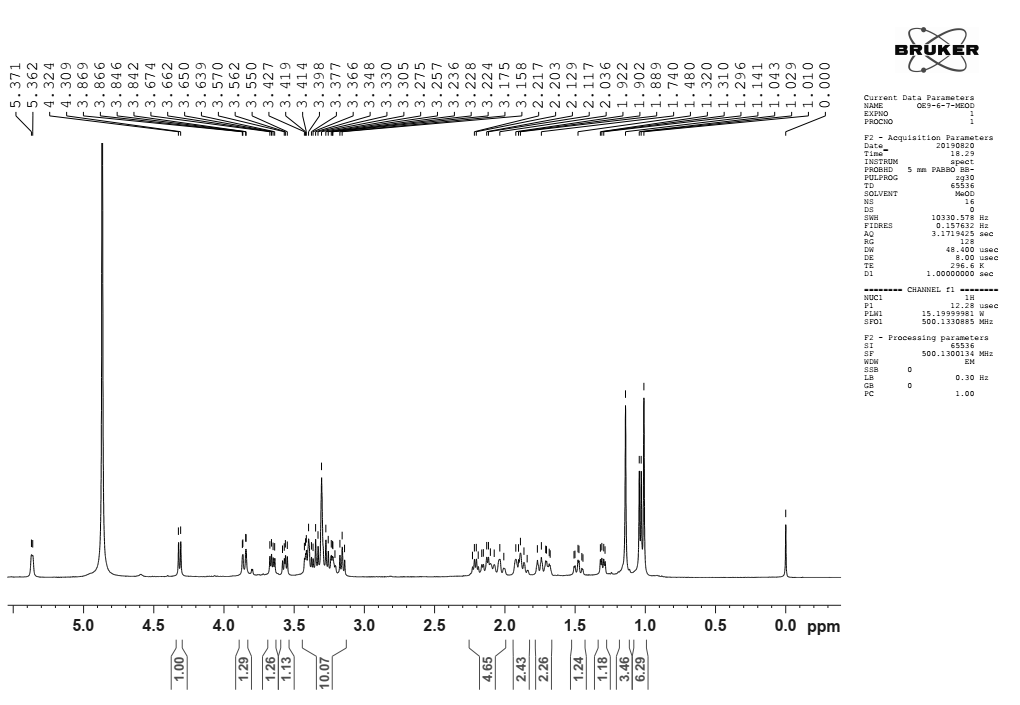


**Figure S9.** ^1^H NMR (500 MHz, CD_3_OD) spectrum of **2**


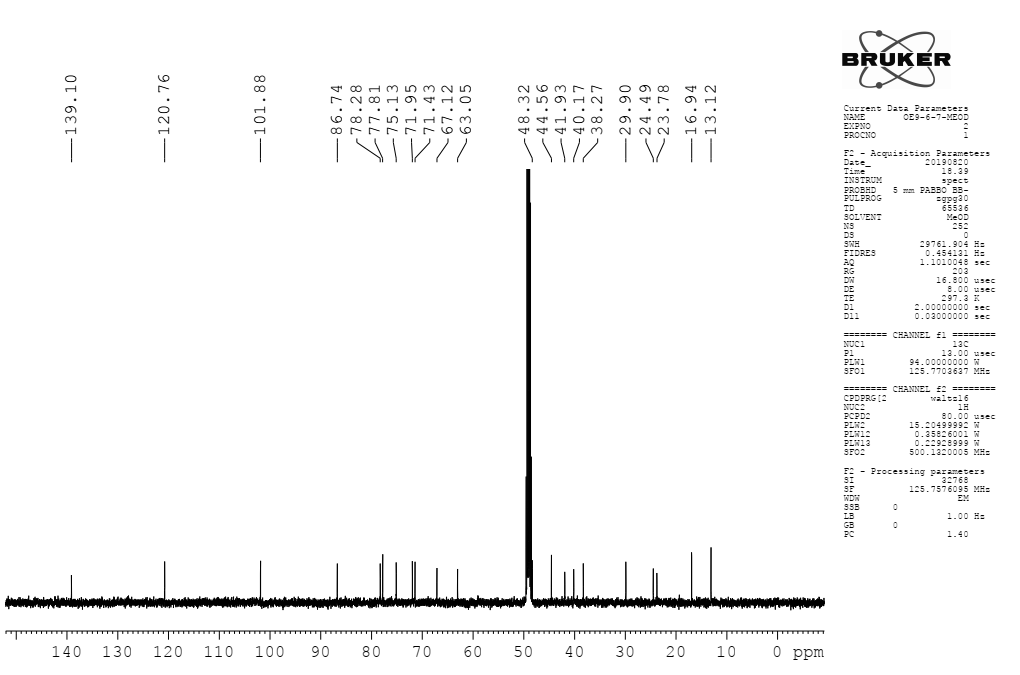


**Figure S10.** ^13^C NMR (125 MHz, CD_3_OD) spectrum of **2**


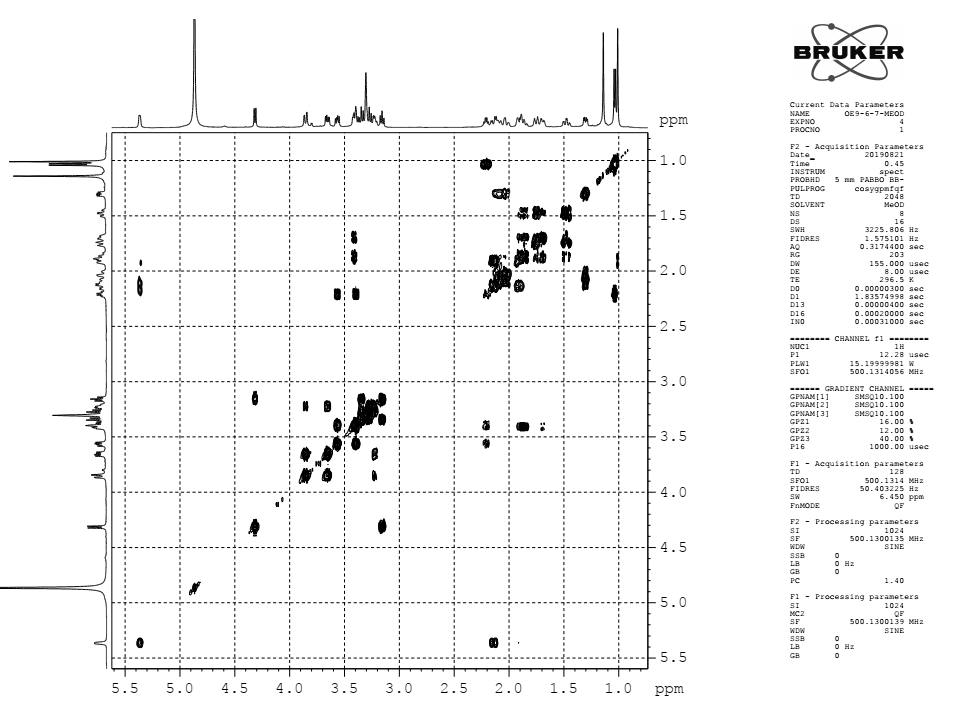


**Figure S11.** ^1^H COSY (CD_3_OD) spectrum of **2**


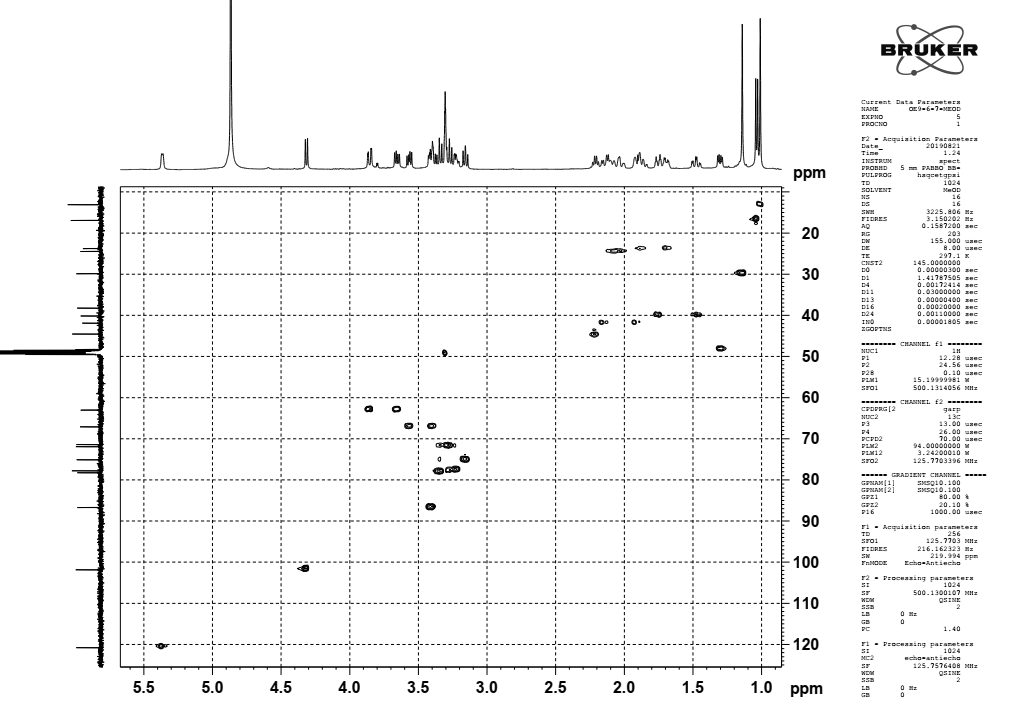


**Figure S12.** HSQC (CD_3_OD) spectrum of **2**


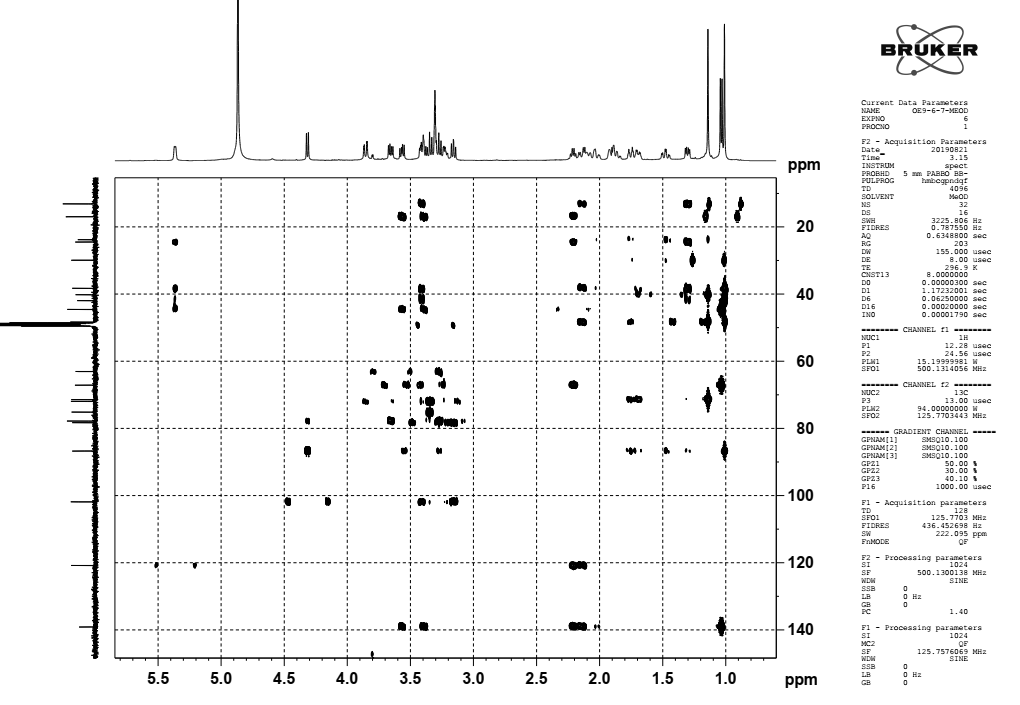


**Figure S13.** HMBC (CD_3_OD) spectrum of **2**


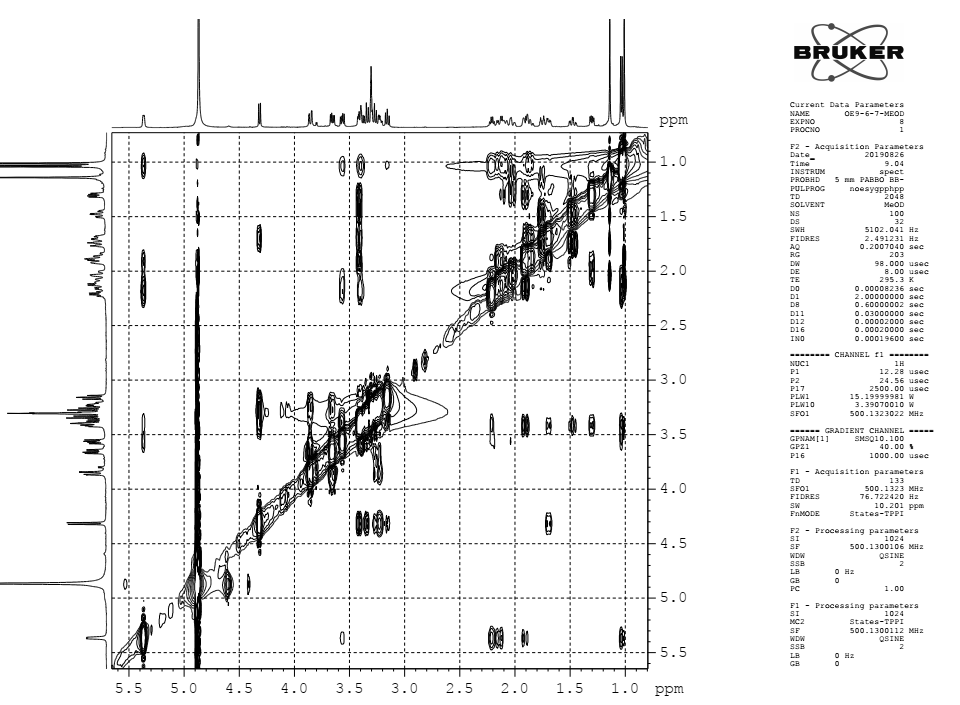


**Figure S14.** NOESY (CD_3_OD) spectrum of **2**


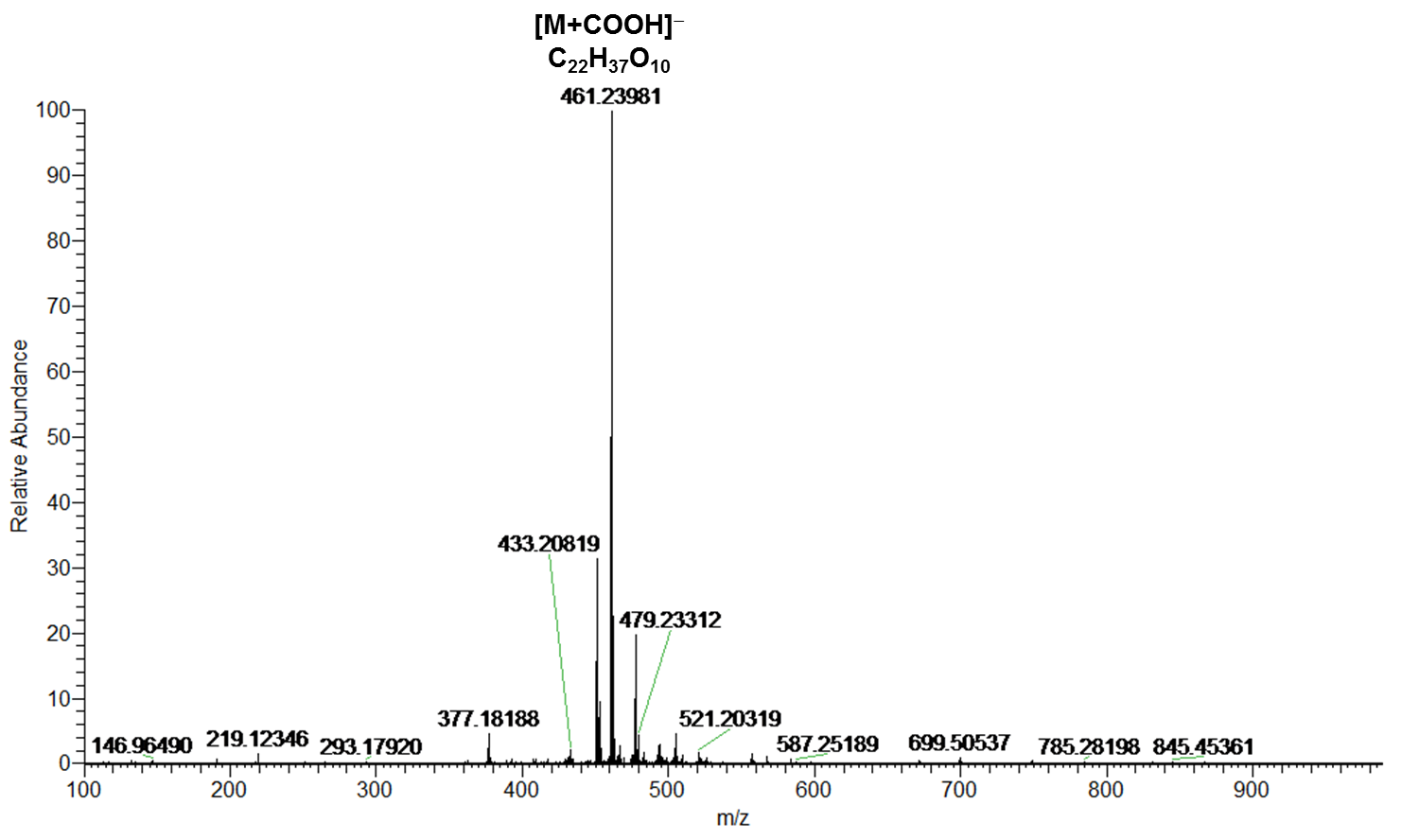


**Figure S15.** ESI-Q-Orbitrap-MS spectrum of **2**

**Figure S16.** IR spectrum of **2**


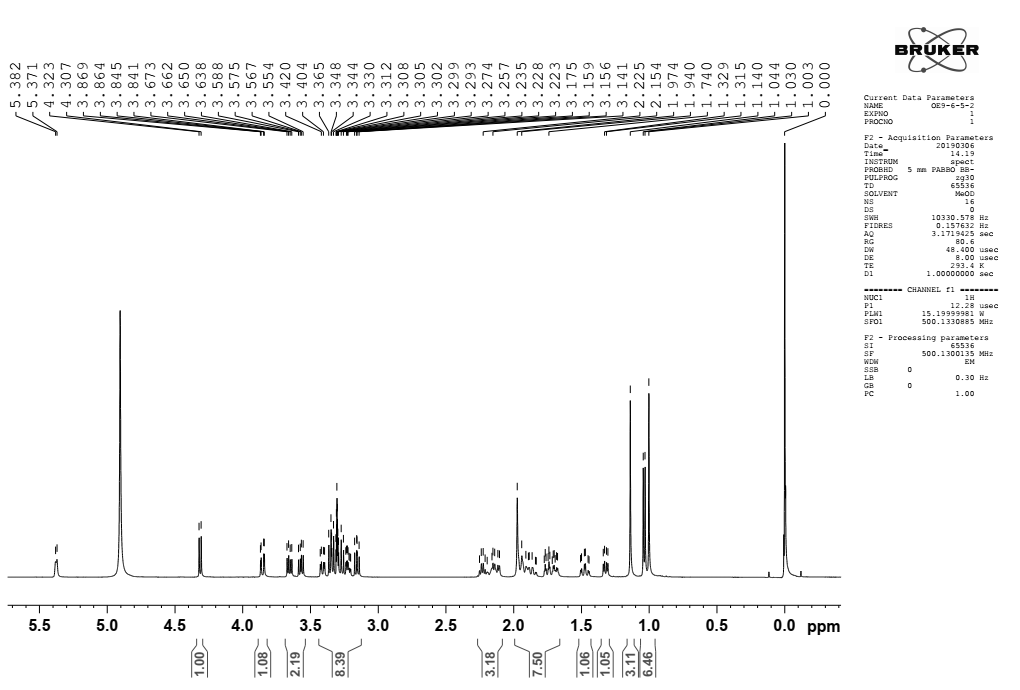


**Figure S17.** ^1^H NMR (500 MHz, CD_3_OD) spectrum of **3**


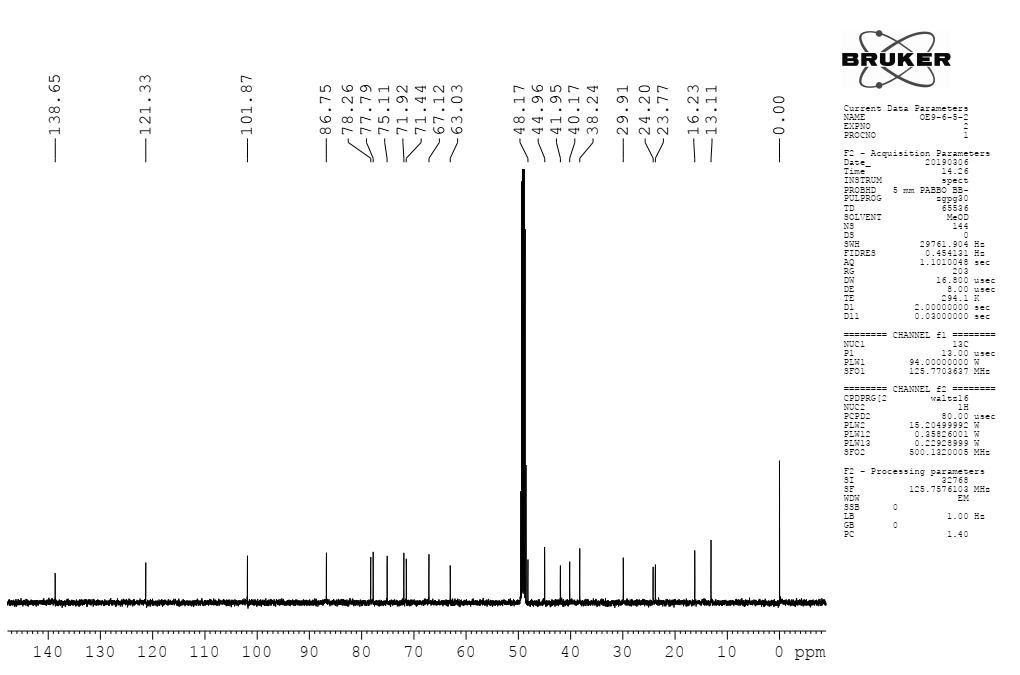


**Figure S18.** ^13^C NMR (125 MHz, CD_3_OD) spectrum of **3**


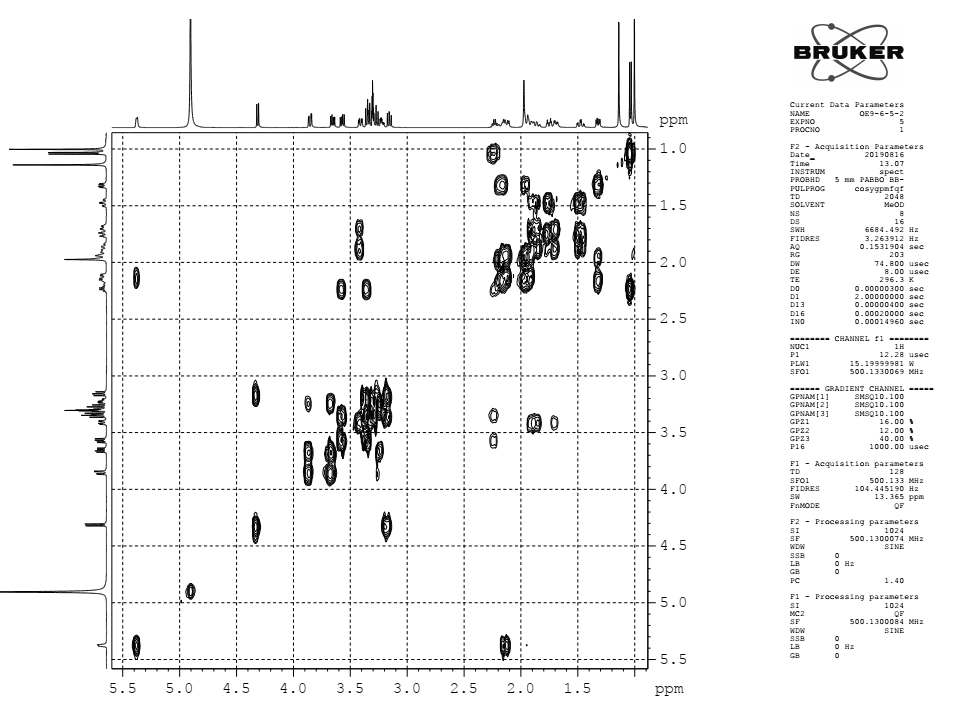


**Figure S19.** ^1^H ^1^H COSY (CD_3_OD) spectrum of **3**


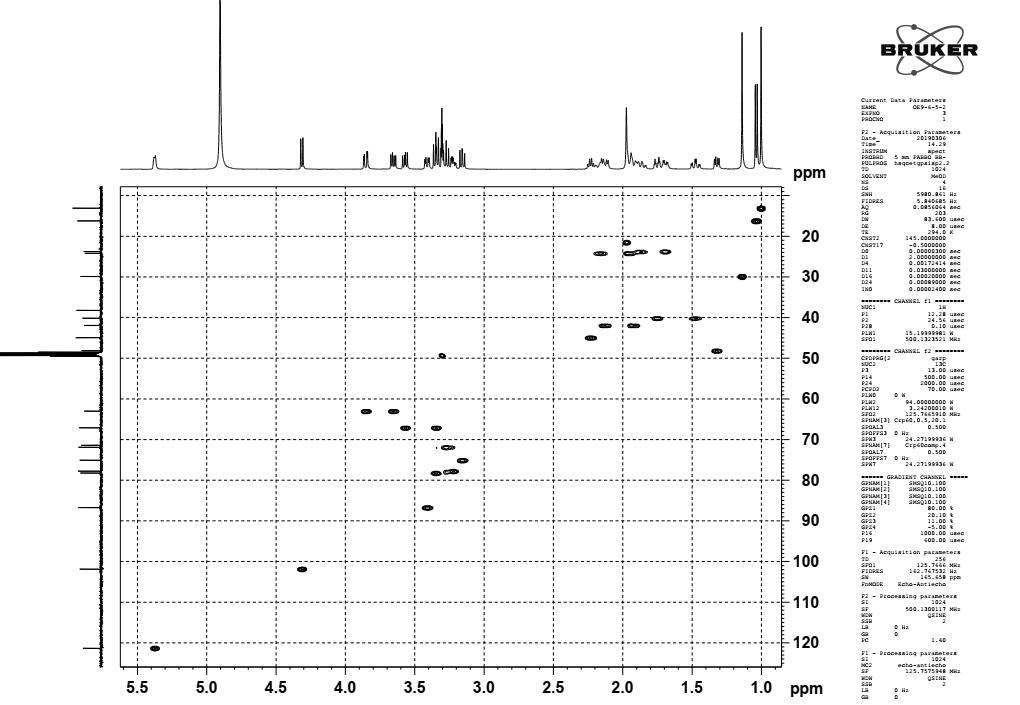


**Figure S20.** HSQC (CD_3_OD) spectrum of **3**


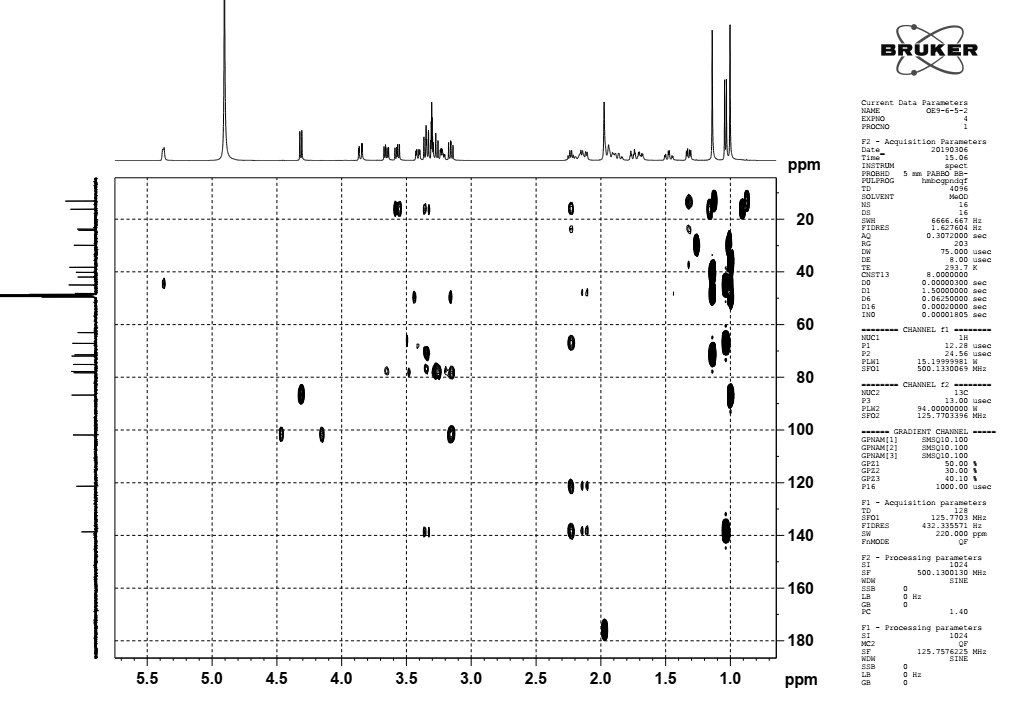


**Figure S21.** HMBC (CD_3_OD) spectrum of **3**


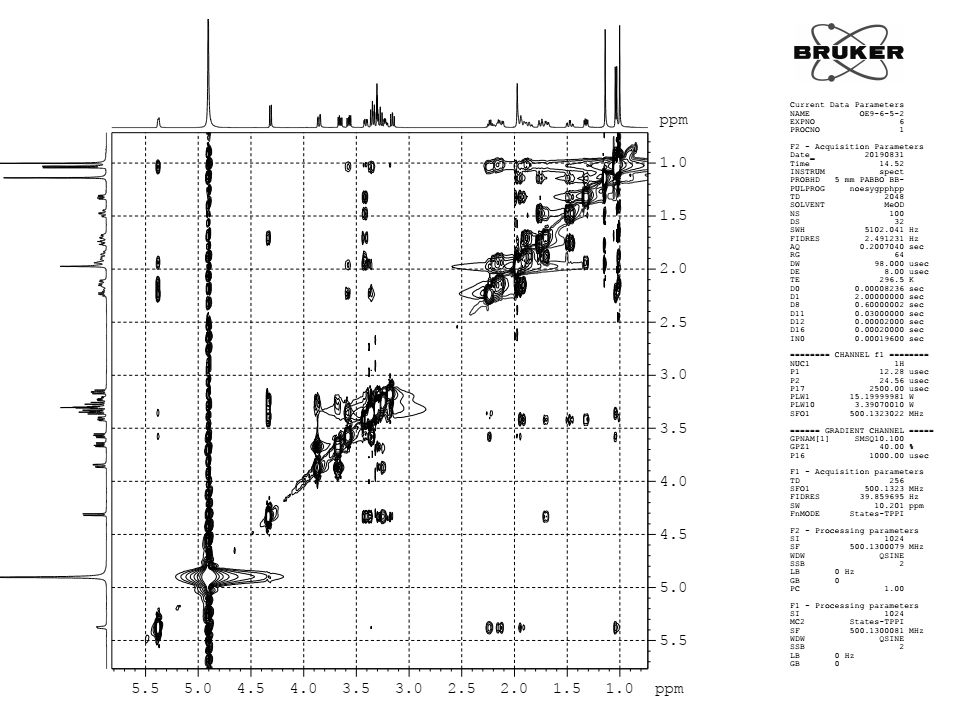


**Figure S22.** NOESY (CD_3_OD) spectrum of **3**


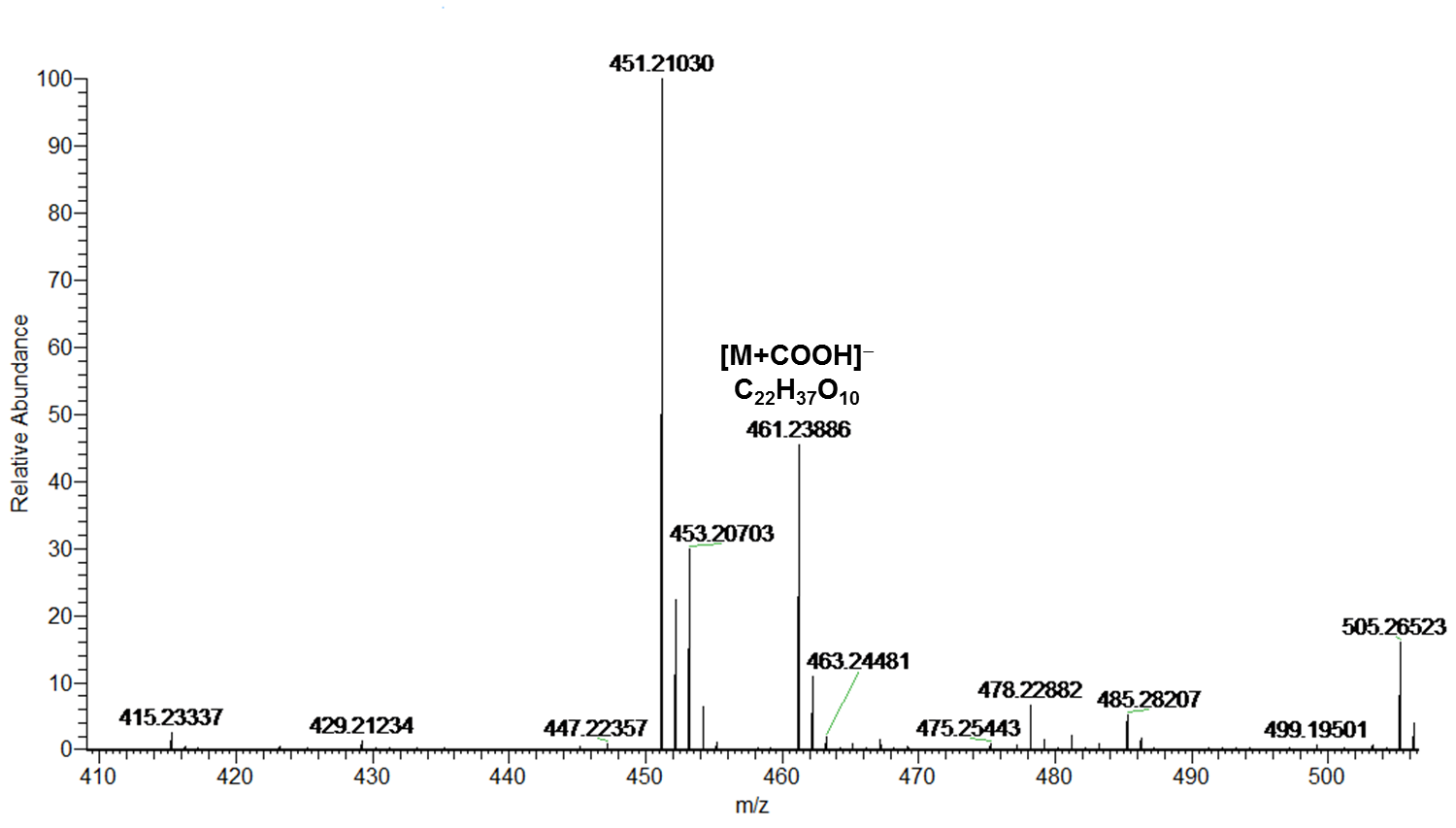


**Figure S23.** ESI-Q-Orbitrap-MS spectrum of **3**

**Figure S24.** IR spectrum of **3**


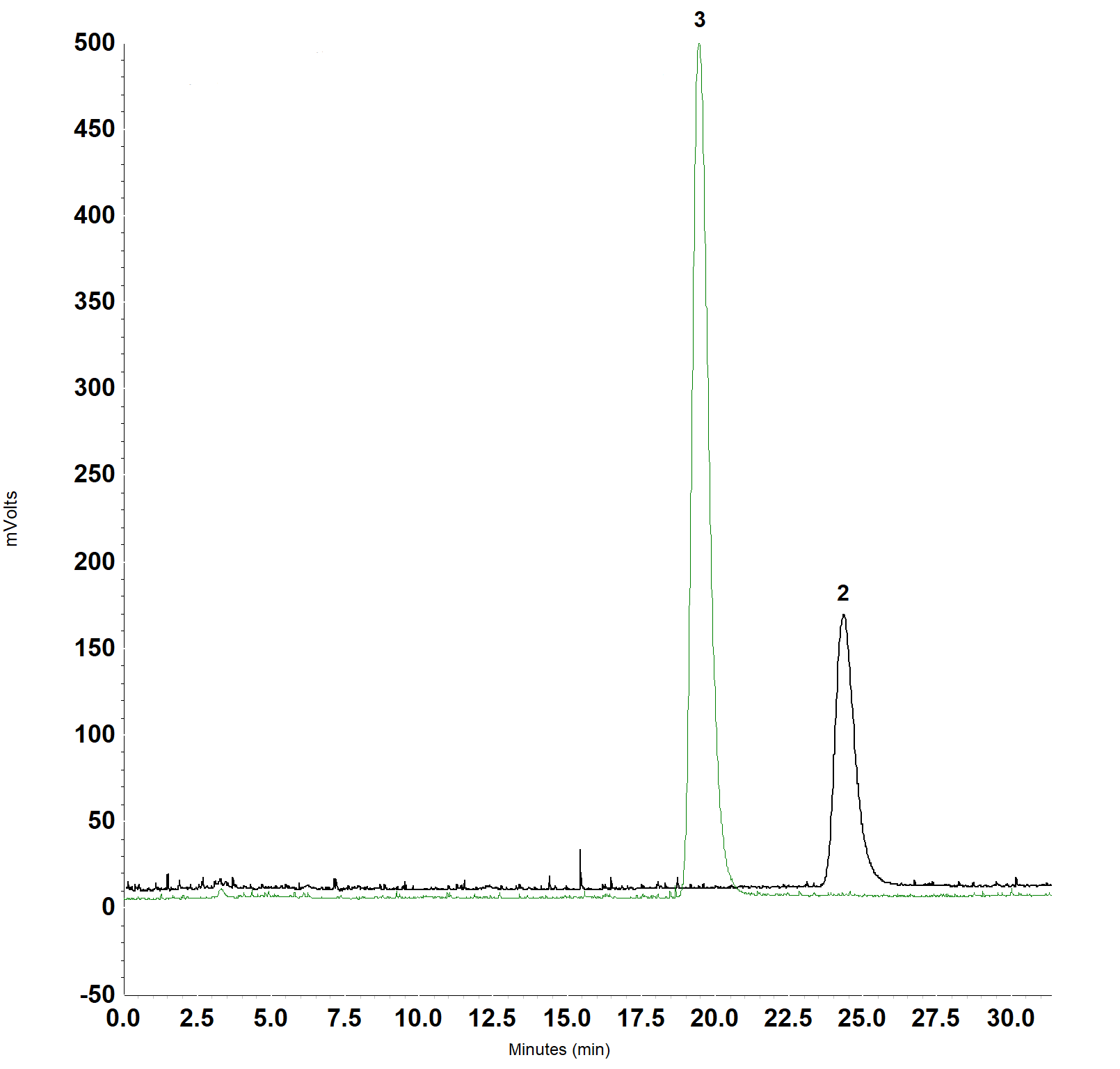


**Figure S25.** The HPLC analysis spectra of **2** and **3**

Column: Cosmosil 5C18-MS-II (4.6 mm i.d. × 250 mm, 5*µ*m); mobile phase: CH_3_CN-H_2_O = 16:84 (v/v); column temperature: 30°C; flow rate: 0.7 mL/min; injection volume: 10*µ*L.


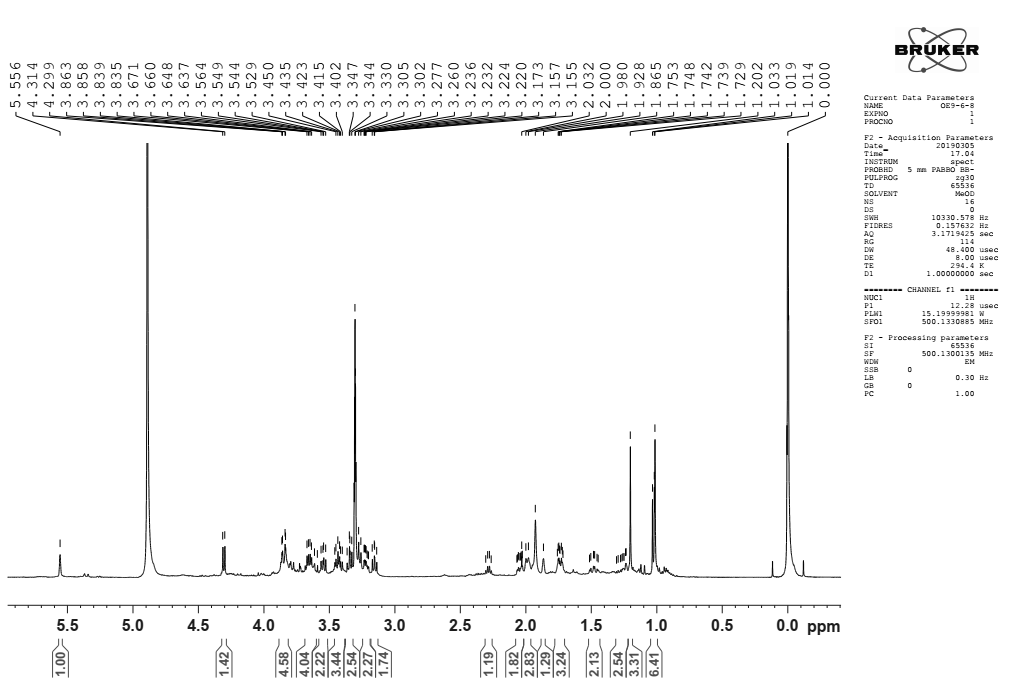


**Figure S26.** ^1^H NMR (500 MHz, CD_3_OD) spectrum of **4**


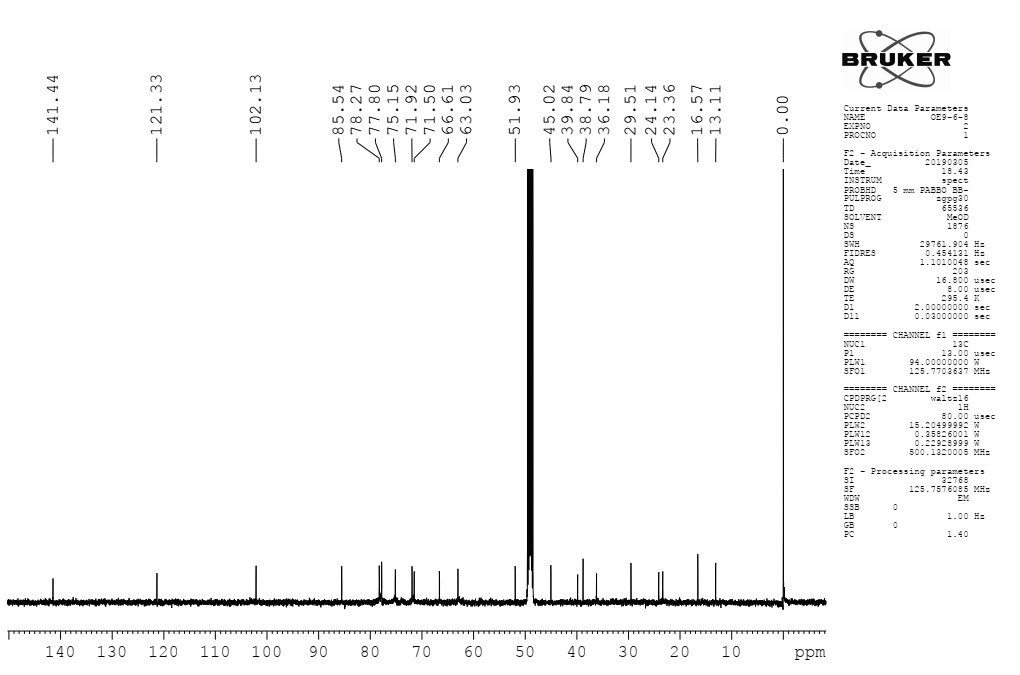


**Figure S27.** ^13^C NMR (125 MHz, CD_3_OD) spectrum of **4**


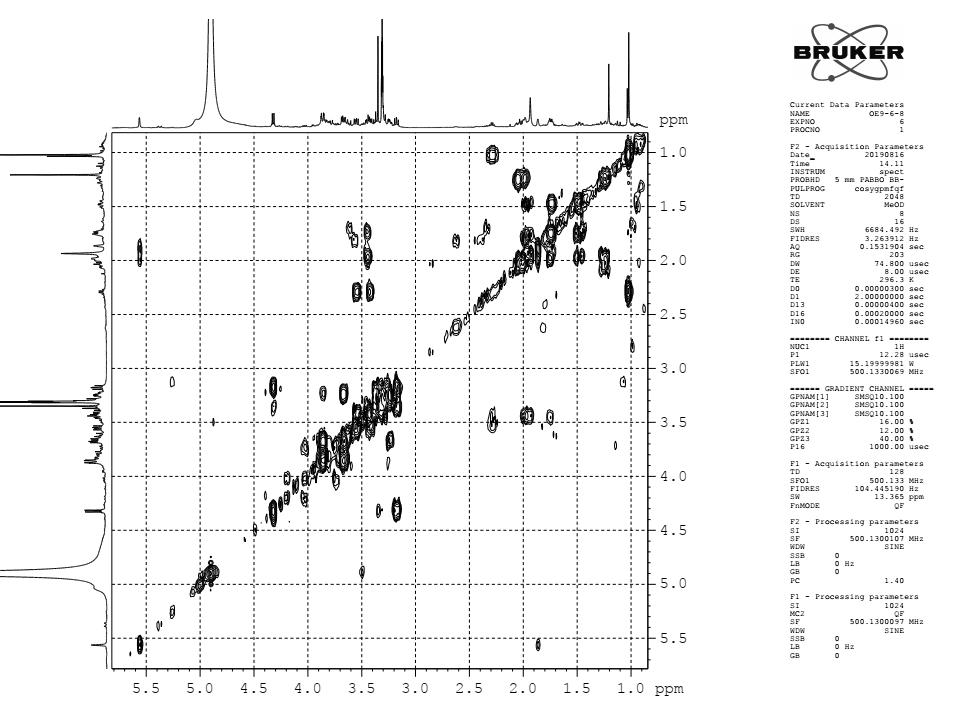


**Figure S28.** ^1^H ^1^H COSY (CD_3_OD) spectrum of **4**


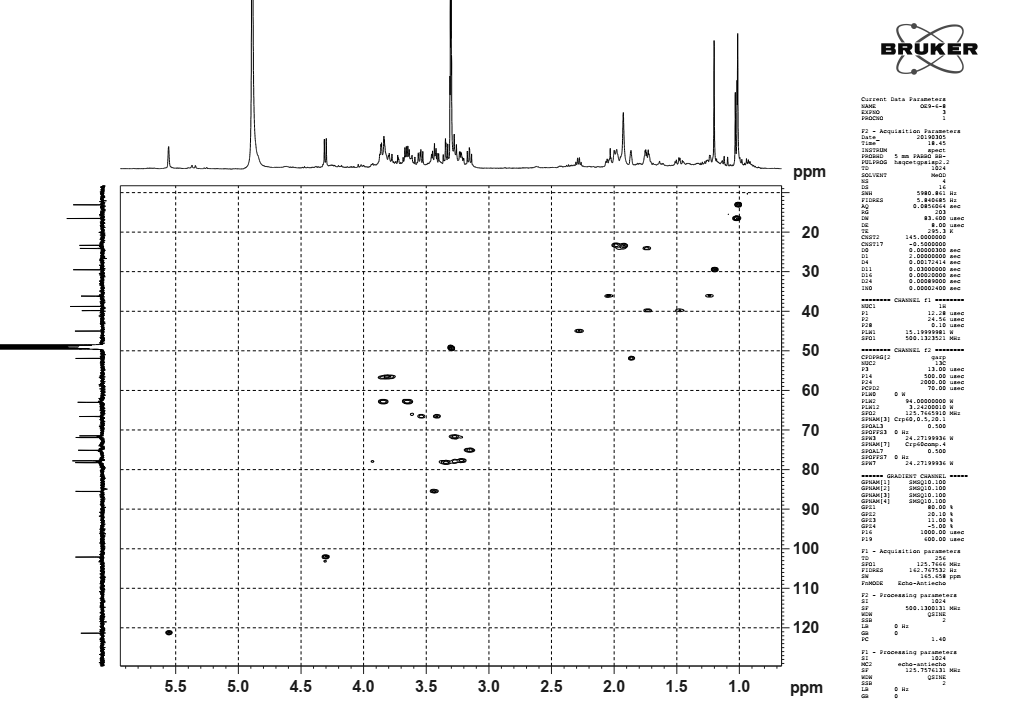


**Figure S29.** HSQC (CD_3_OD) spectrum of **4**


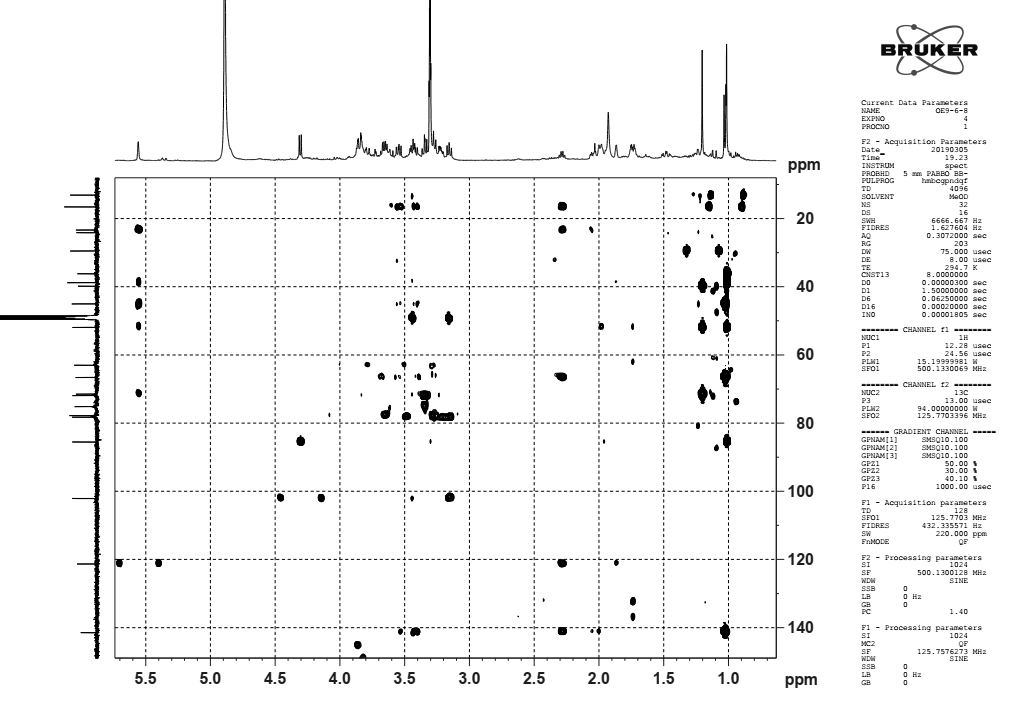


**Figure S30.** HMBC (CD_3_OD) spectrum of **4**


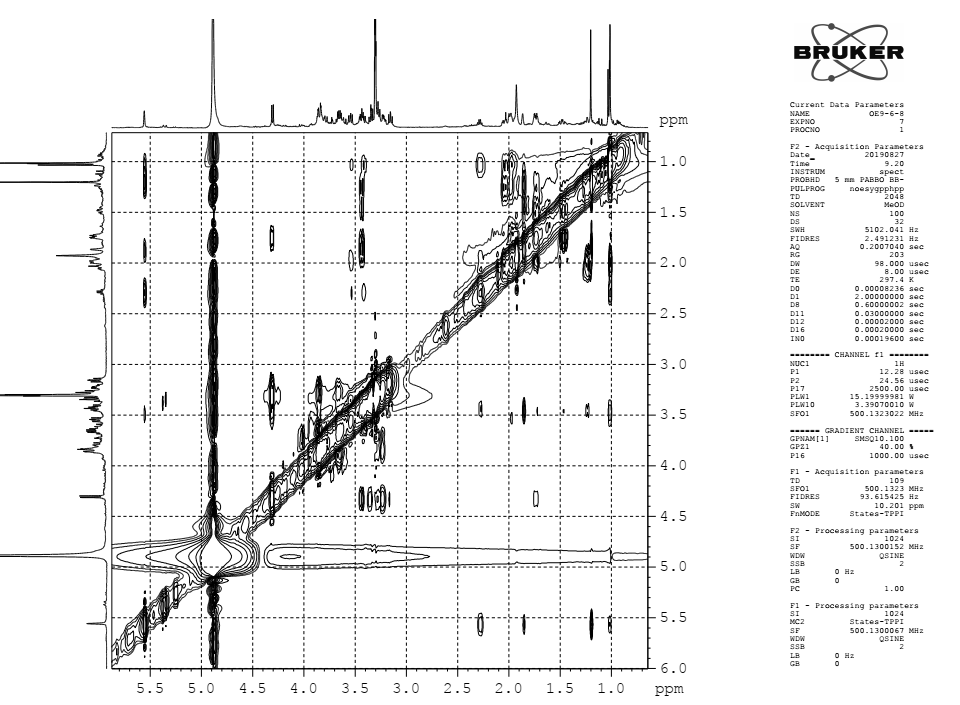


**Figure S31.** NOESY (CD_3_OD) spectrum of **4**


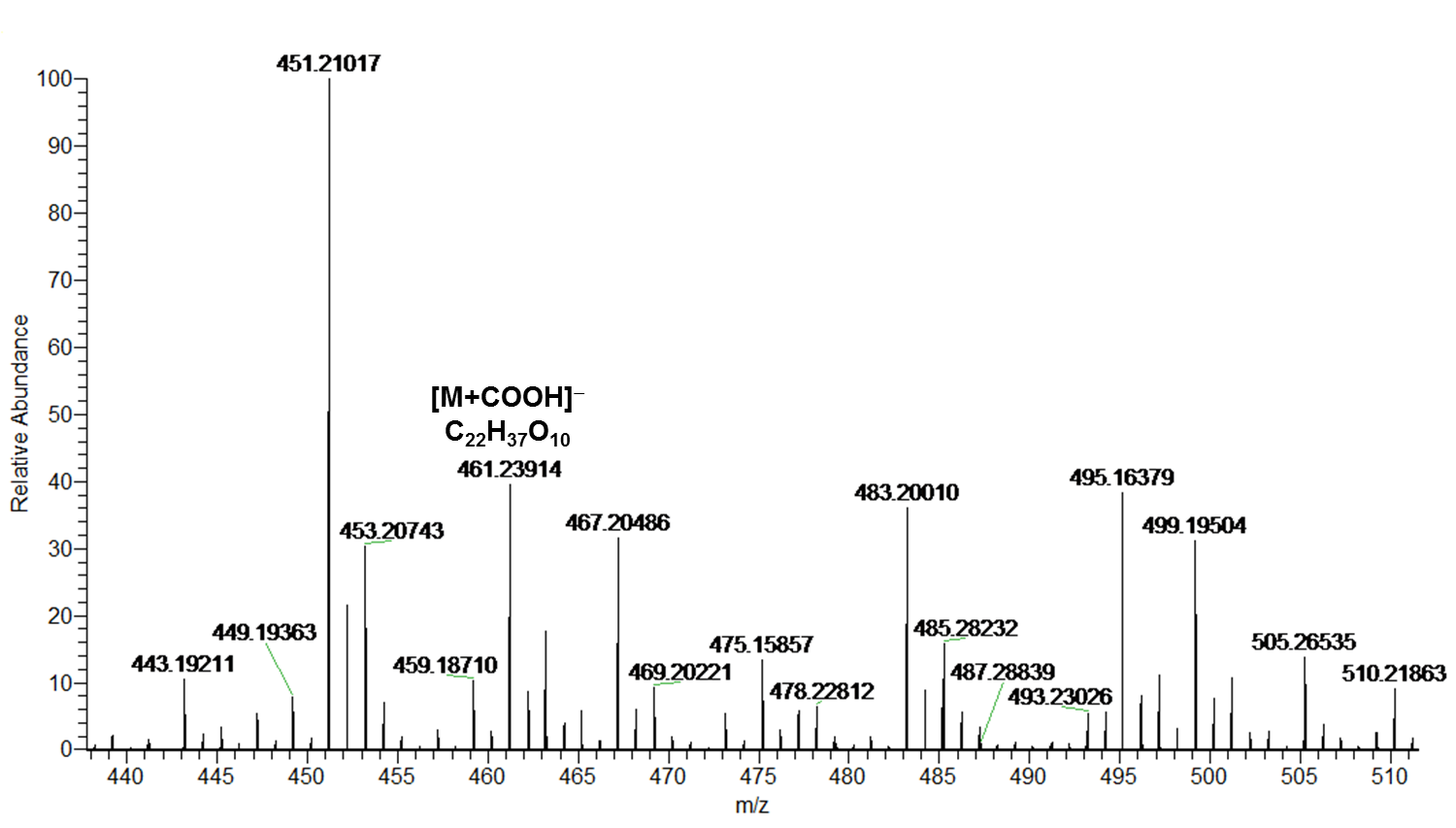


**Figure S32.** ESI-Q-Orbitrap-MS spectrum of **4**

**Figure S33.** IR spectrum of **4**


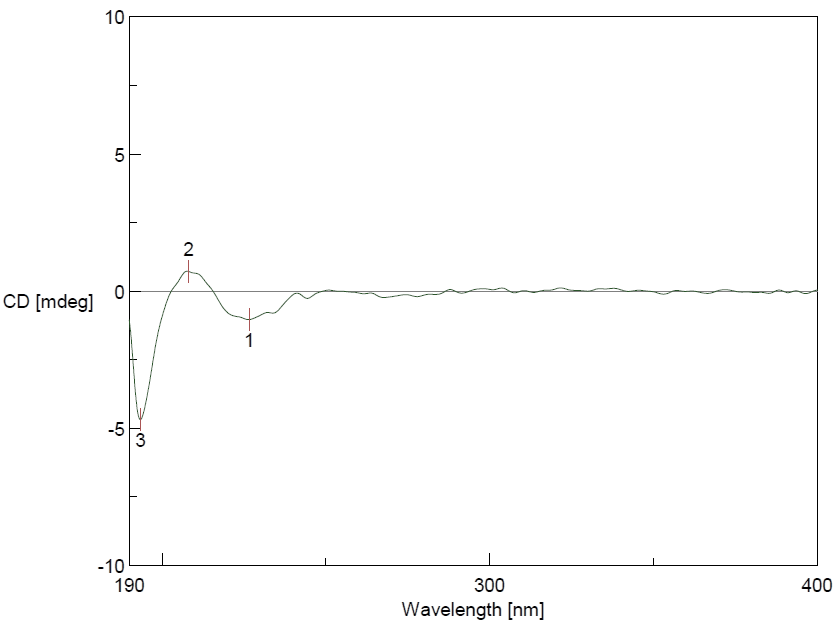


**Figure S34.** CD spectrum of **4**


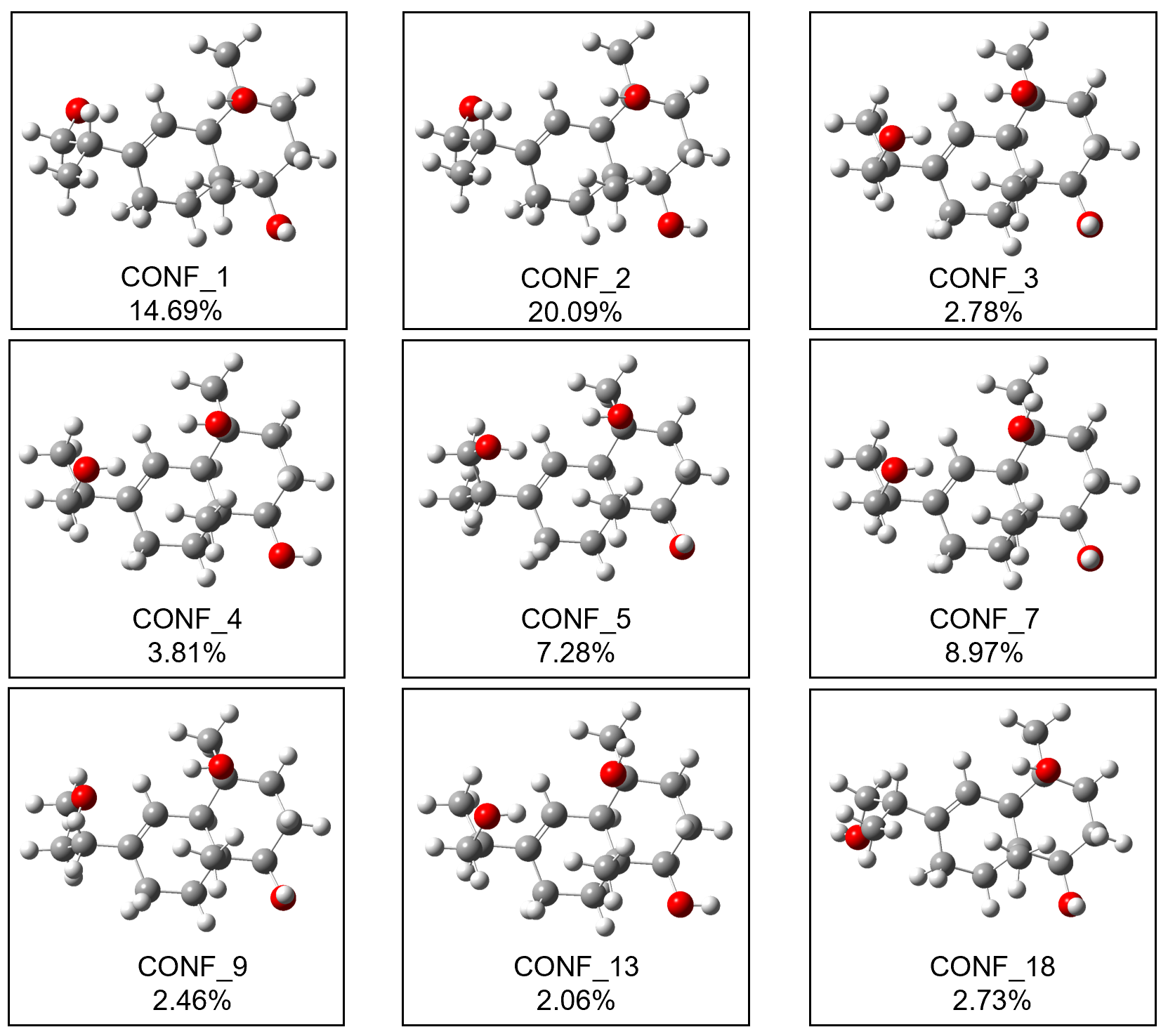


**Figure S35.** The conformational analysis and optimized geometries of predominant conformers of **4**


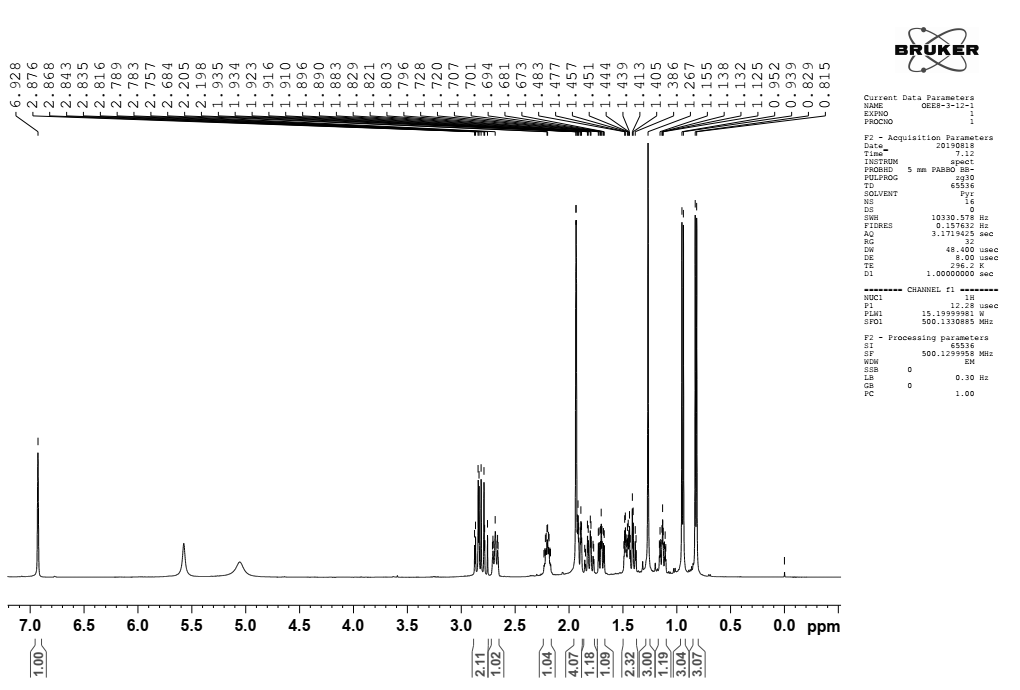


**Figure S36.** ^1^H NMR (500 MHz, C_5_D_5_N) spectrum of **5**


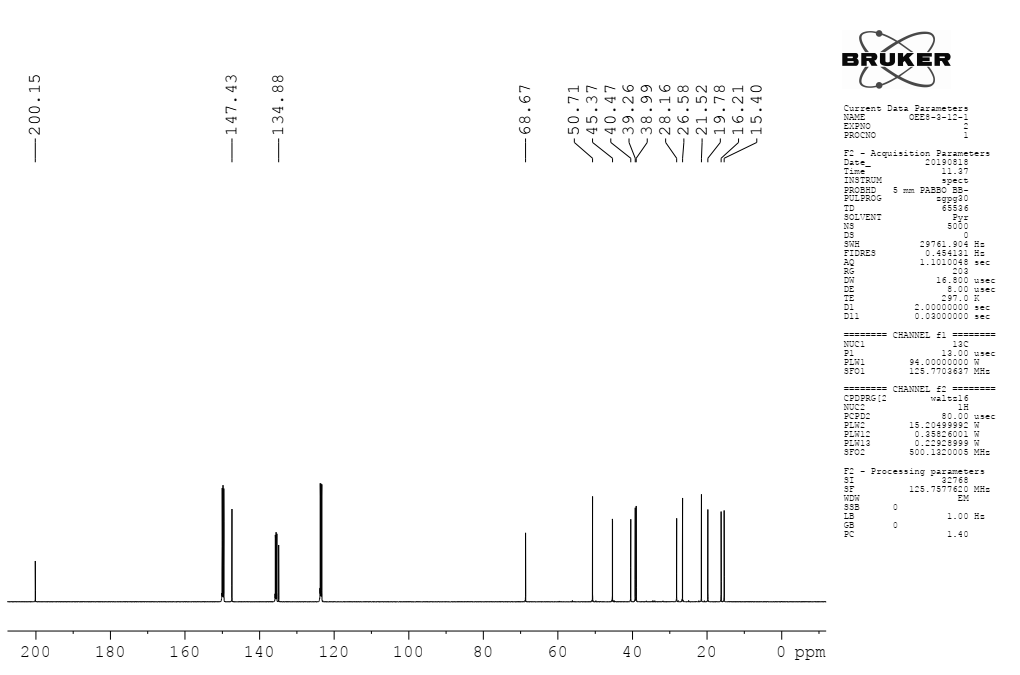


**Figure S37.** ^13^C NMR (125 MHz, C_5_D_5_N) spectrum of **5**


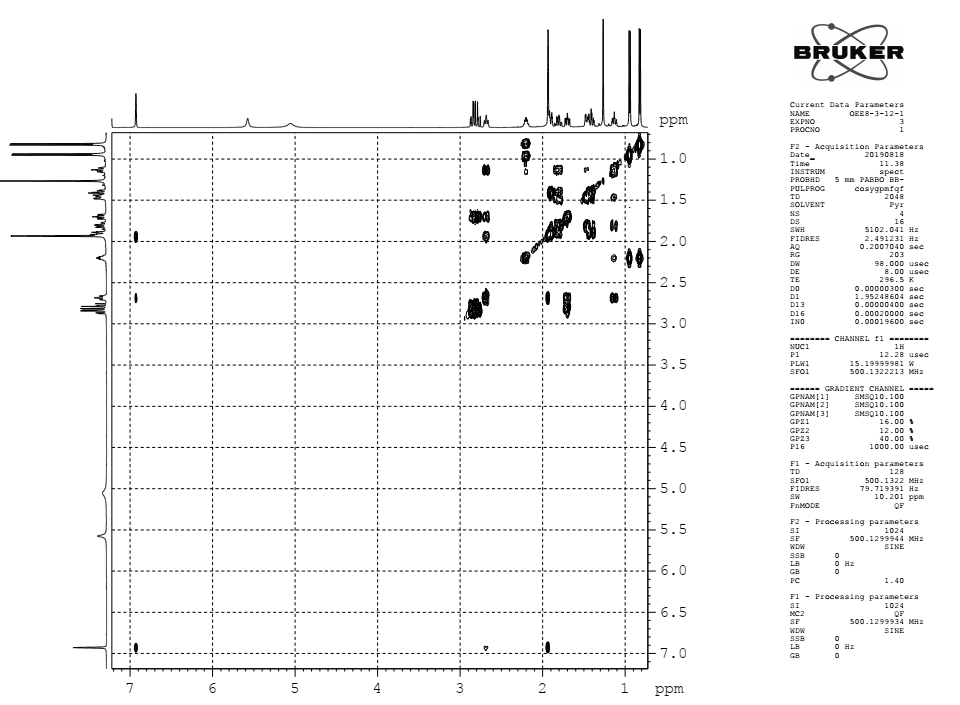


**Figure S38.** ^1^H ^1^H COSY (C_5_D_5_N) spectrum of **5**


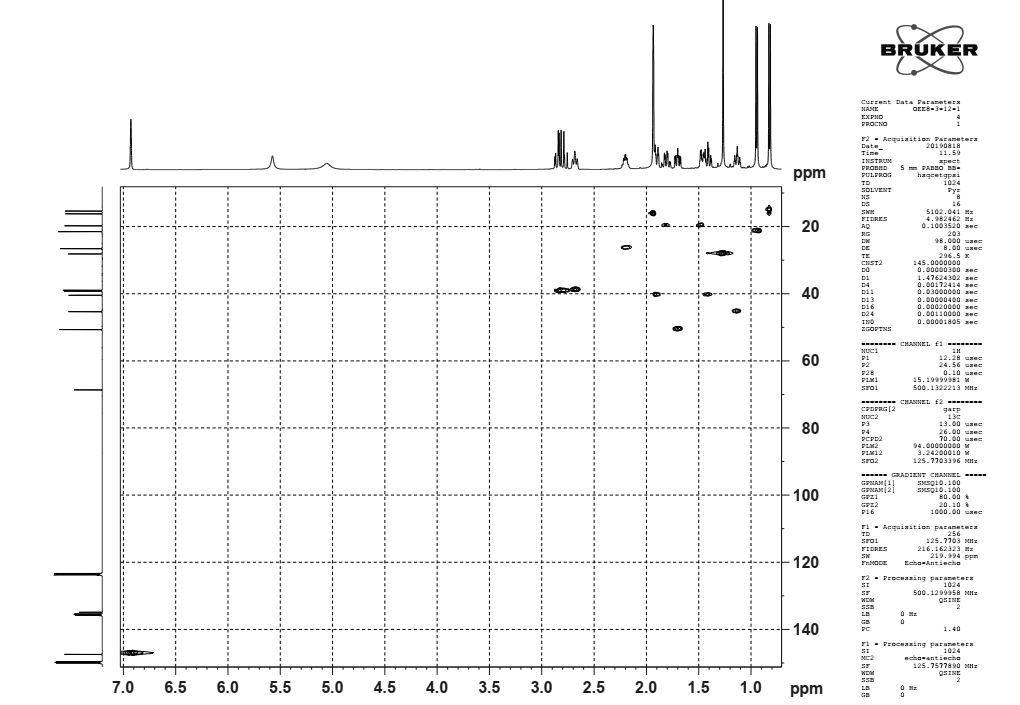


**Figure S39.** HSQC (C_5_D_5_N) spectrum of **5**


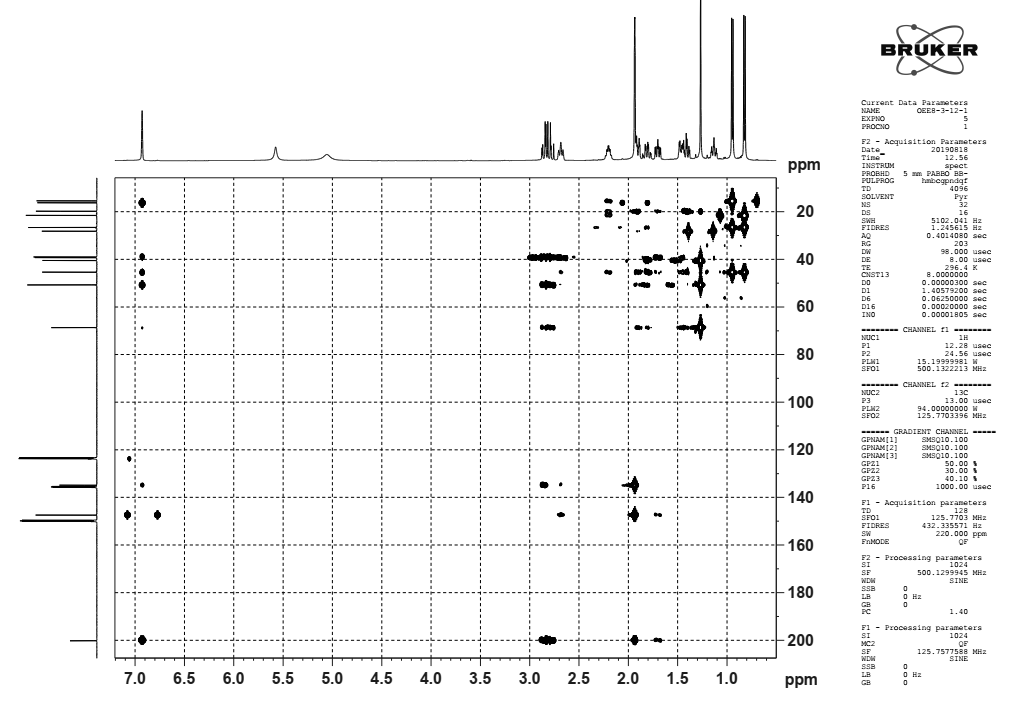


**Figure S40.** HMBC (C_5_D_5_N) spectrum of **5**


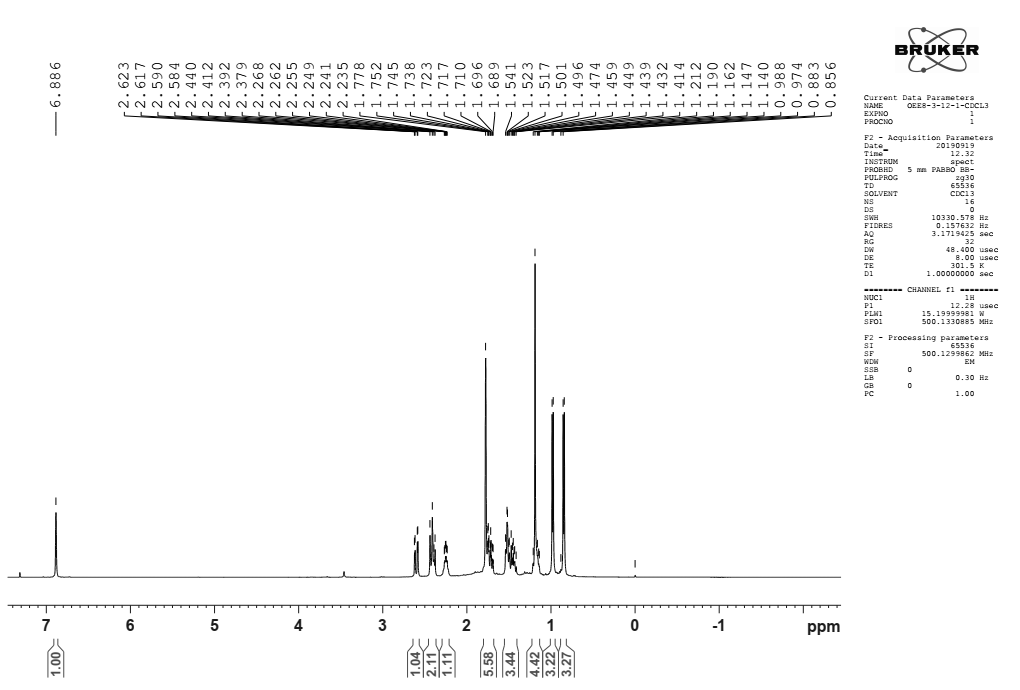


**Figure S41.** ^1^H NMR (500 MHz, CDCl_3_) spectrum of **5**


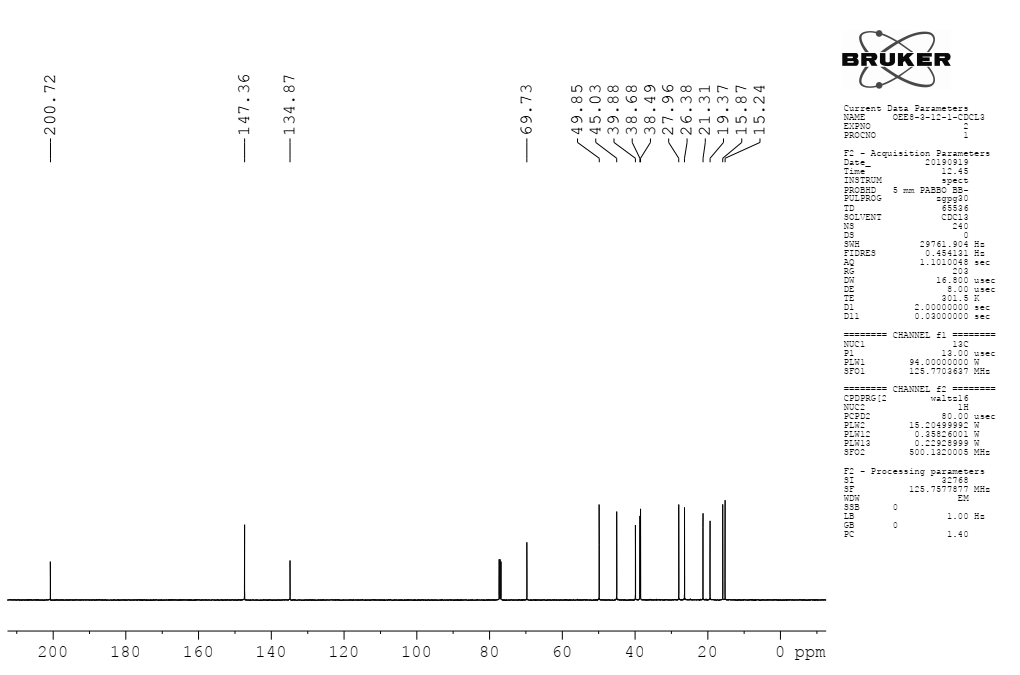


**Figure S42.** ^13^C NMR (125 MHz, CDCl_3_) spectrum of **5**


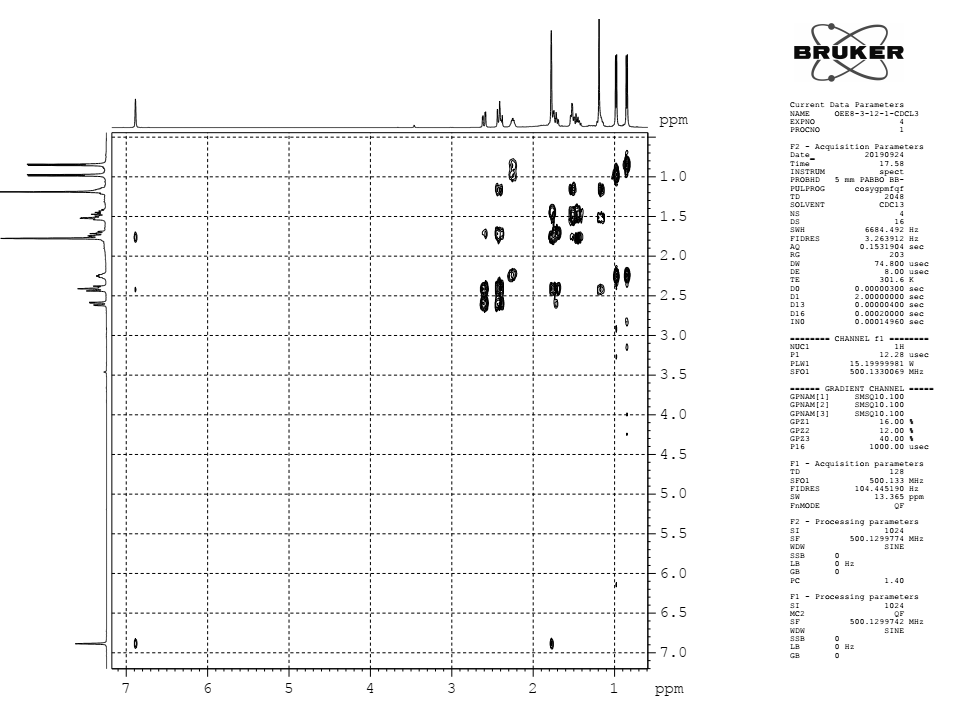


**Figure S43.** ^1^H ^1^H COSY (CDCl_3_) spectrum of **5**


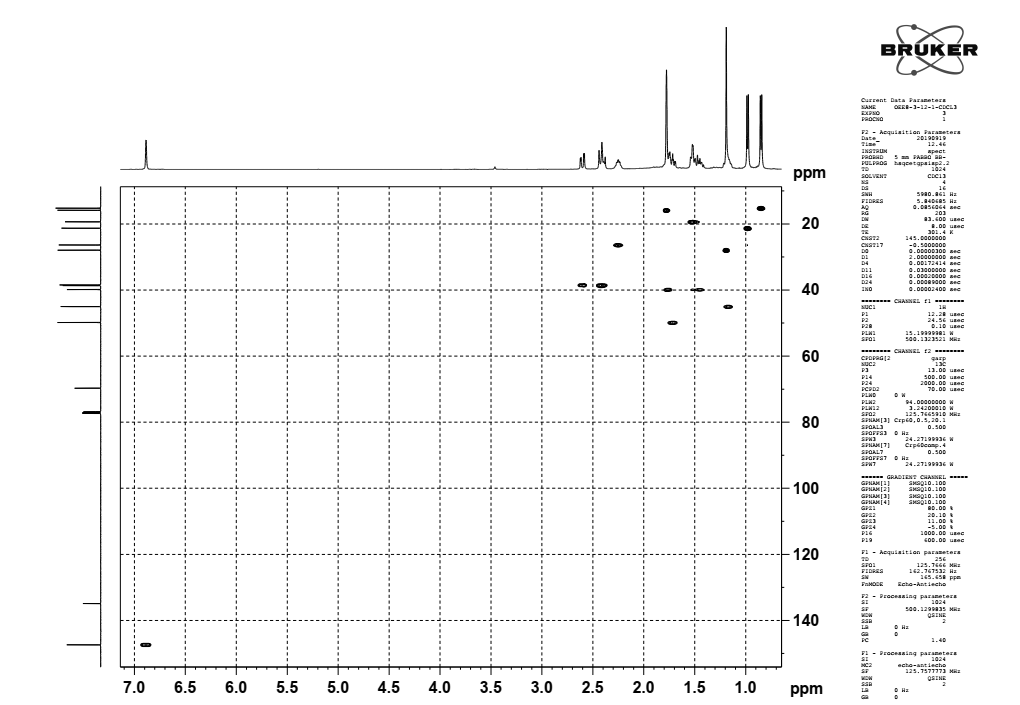


**Figure S44.** HSQC (CDCl_3_) spectrum of **5**


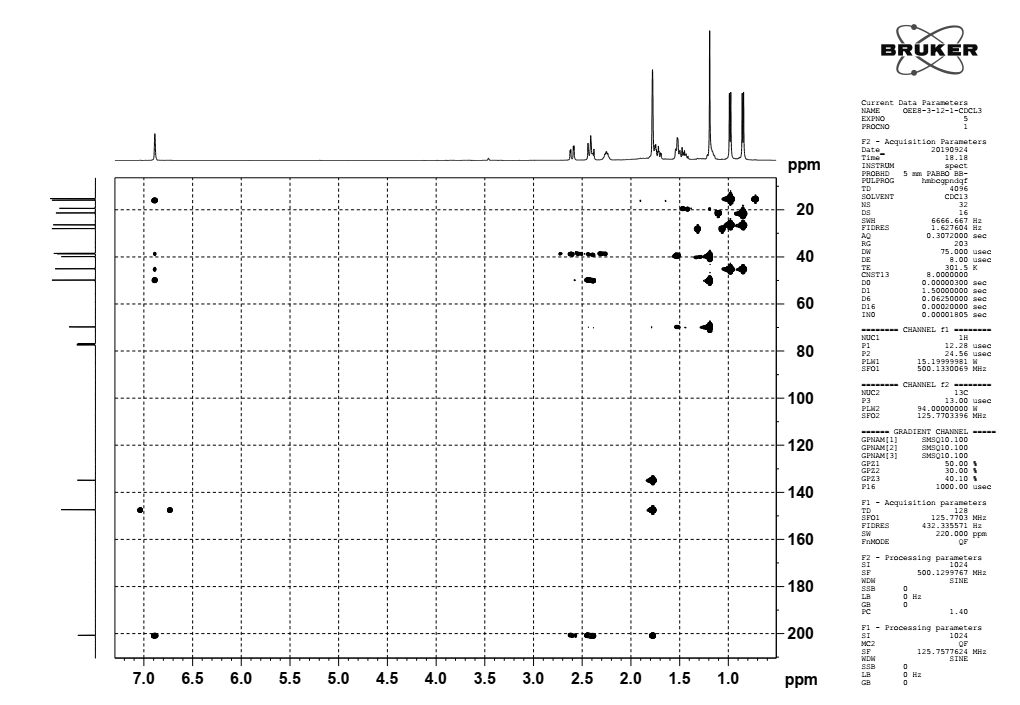


**Figure S45.** HMBC (CDCl_3_) spectrum of **5**


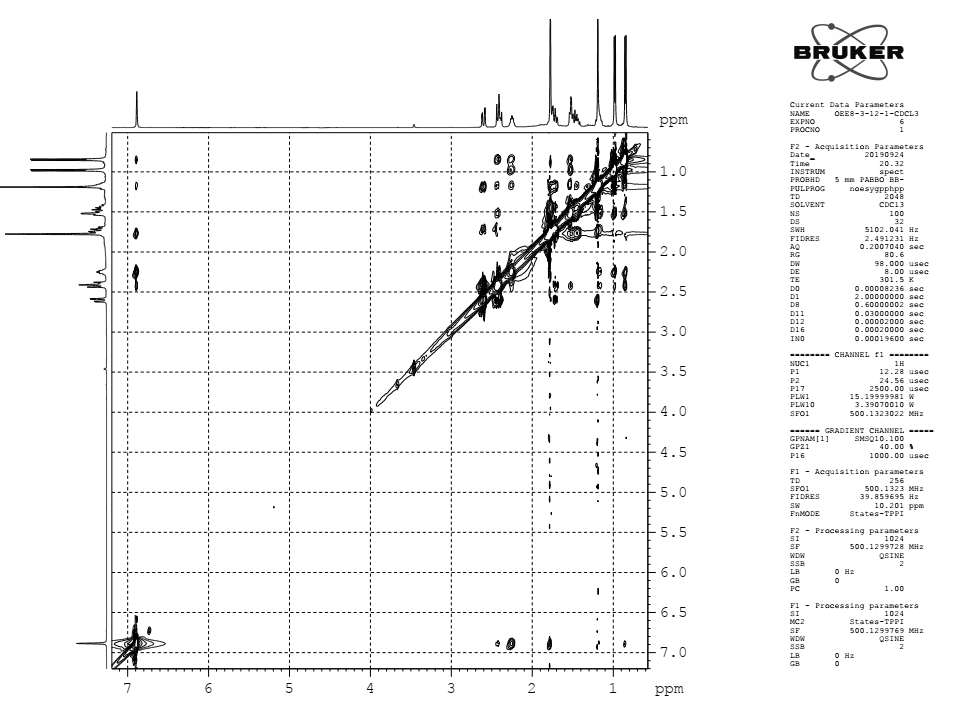


**Figure S46.** NOESY (CDCl_3_) spectrum of **5**


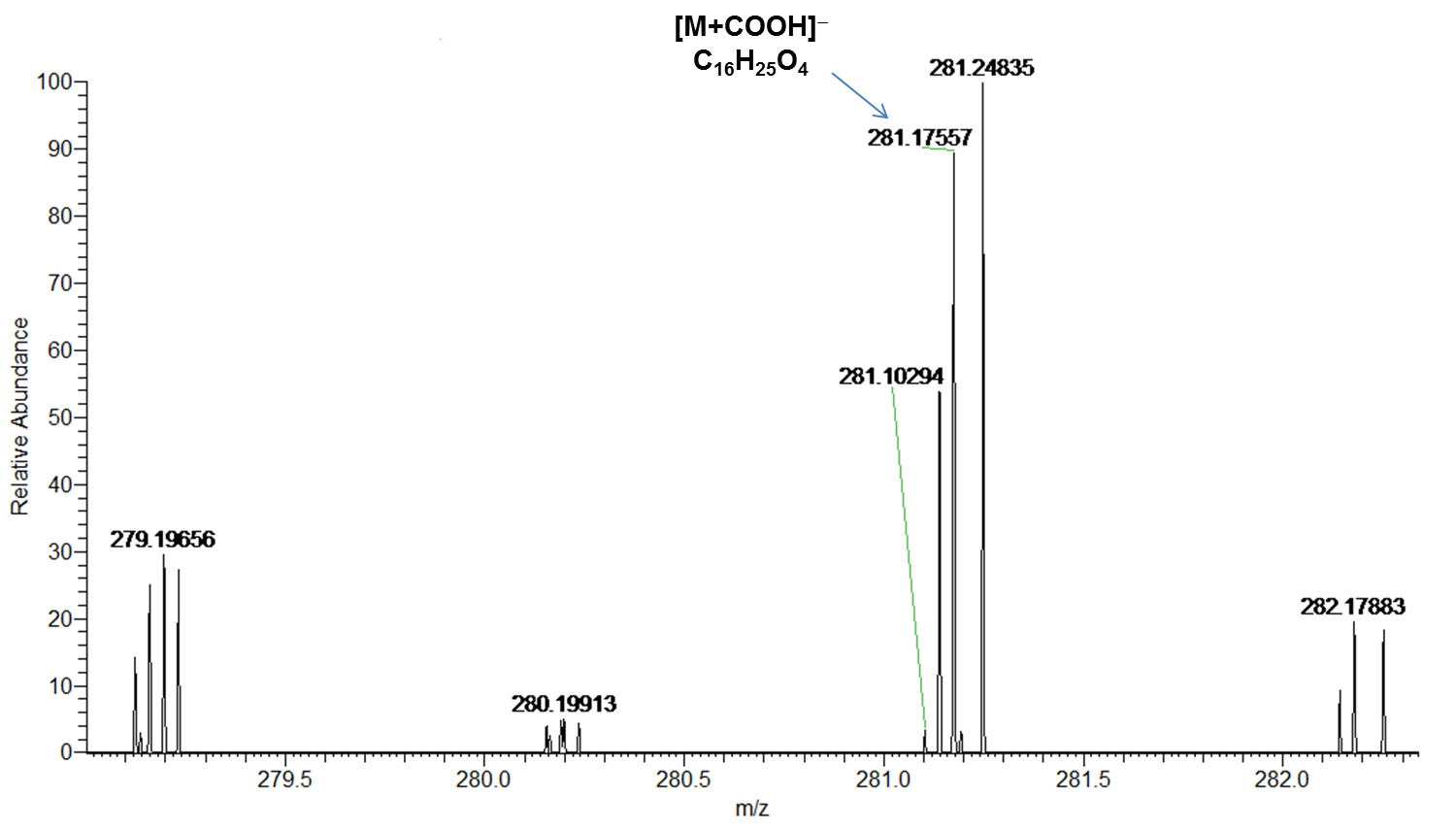


**Figure S47.** ESI-Q-Orbitrap-MS spectrum of **5**


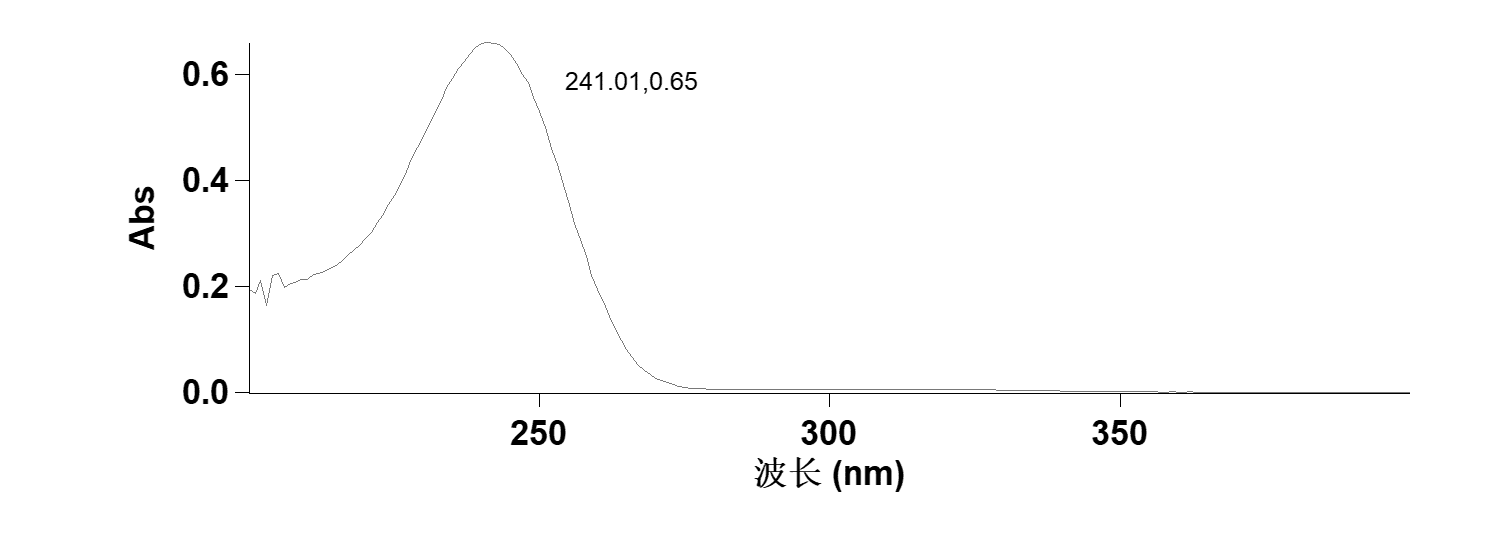


**Figure S48.** UV spectrum of **5**

**Figure S49.** IR spectrum of **5**


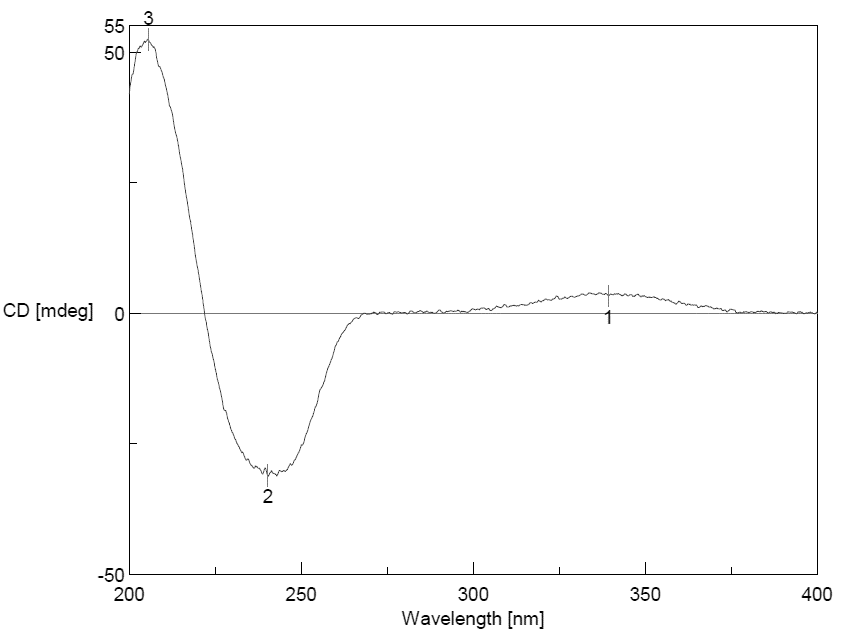


**Figure S50.** CD spectrum of **5**


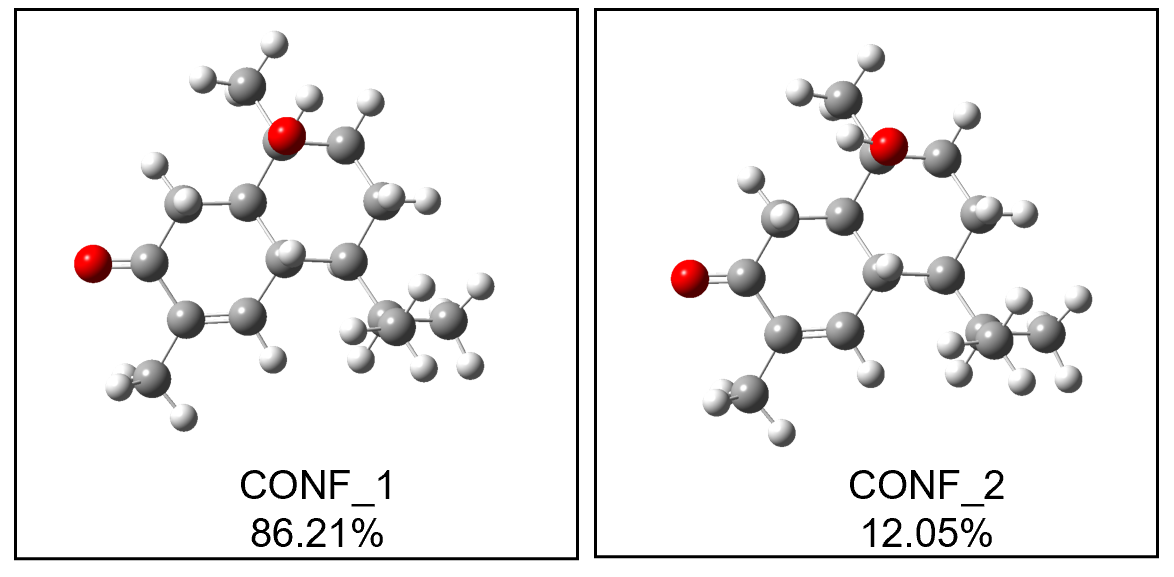


**Figure S51.** The conformational analysis and optimized geometries of predominant conformers of **5**


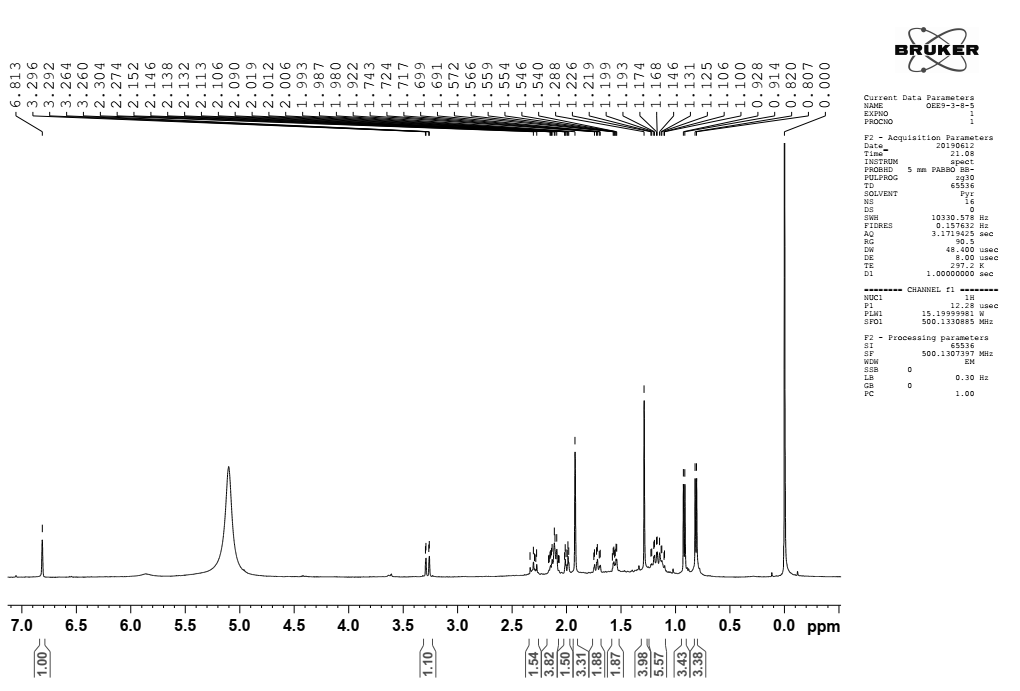


**Figure S52.** ^1^H NMR (500 MHz, C_5_D_5_N) spectrum of **6**


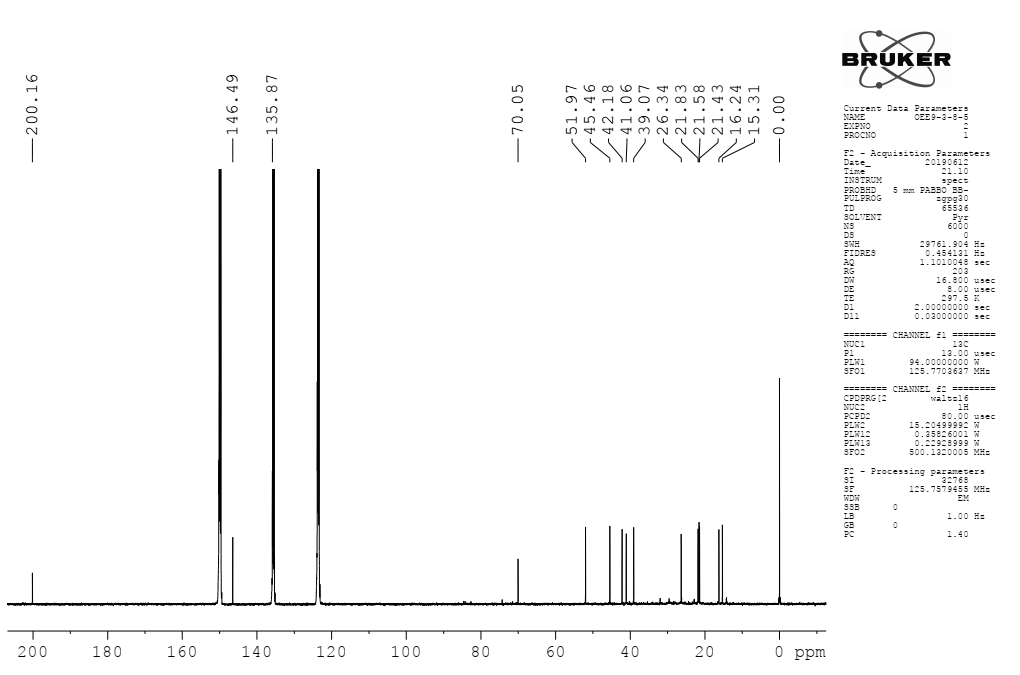


**Figure S53.** ^13^C NMR (125 MHz, C_5_D_5_N) spectrum of **6**


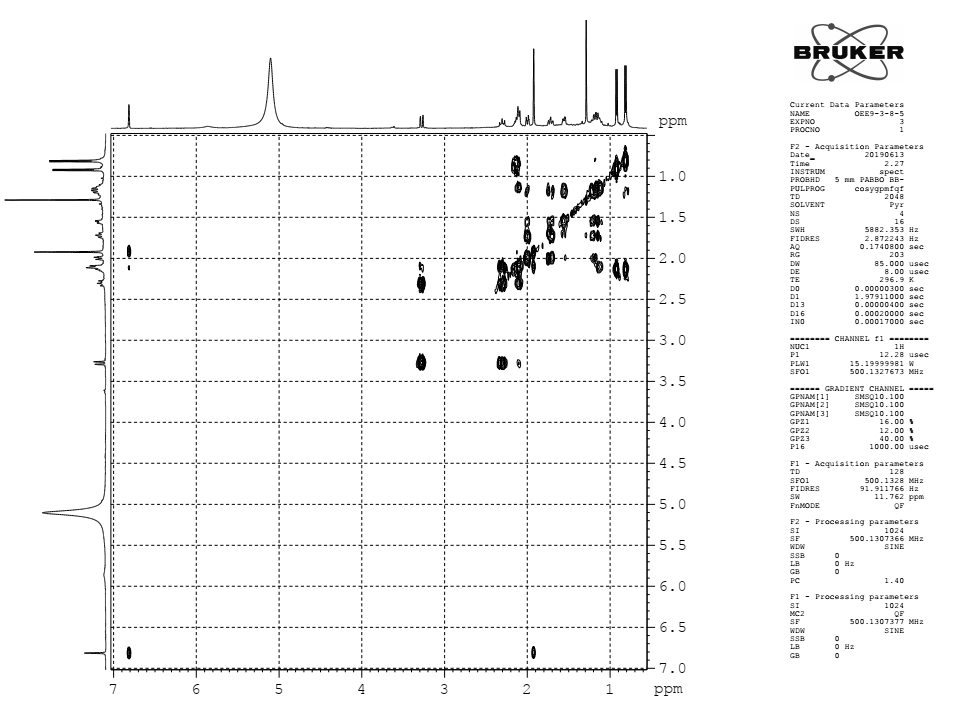


**Figure S54.** ^1^H ^1^H COSY (C_5_D_5_N) spectrum of **6**


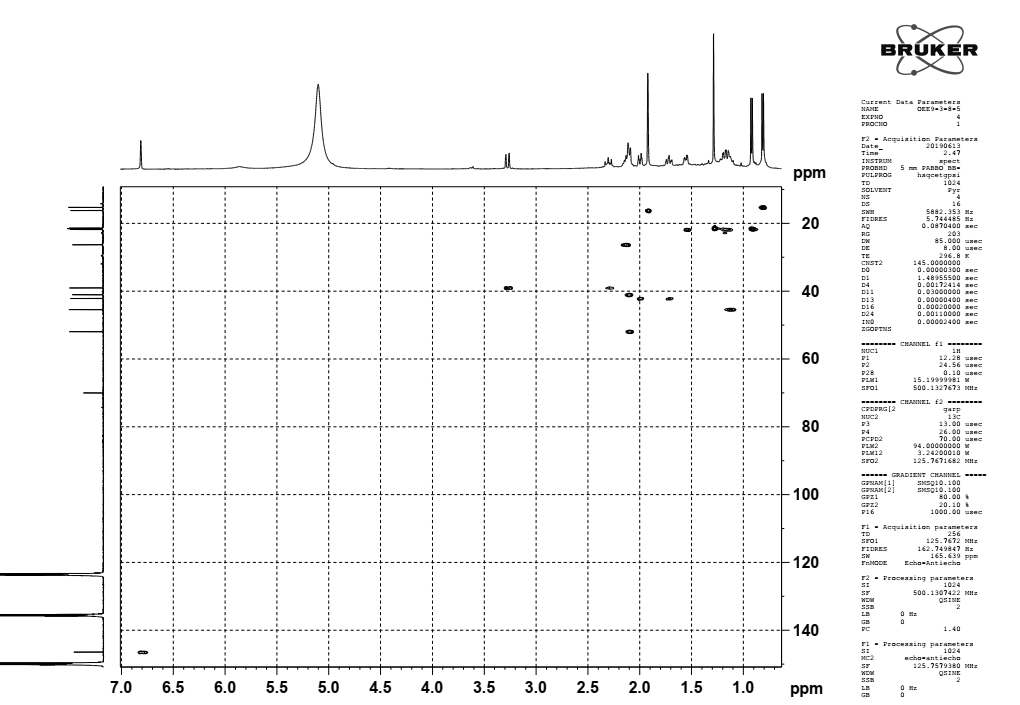


**Figure S55.** HSQC (C_5_D_5_N) spectrum of **6**


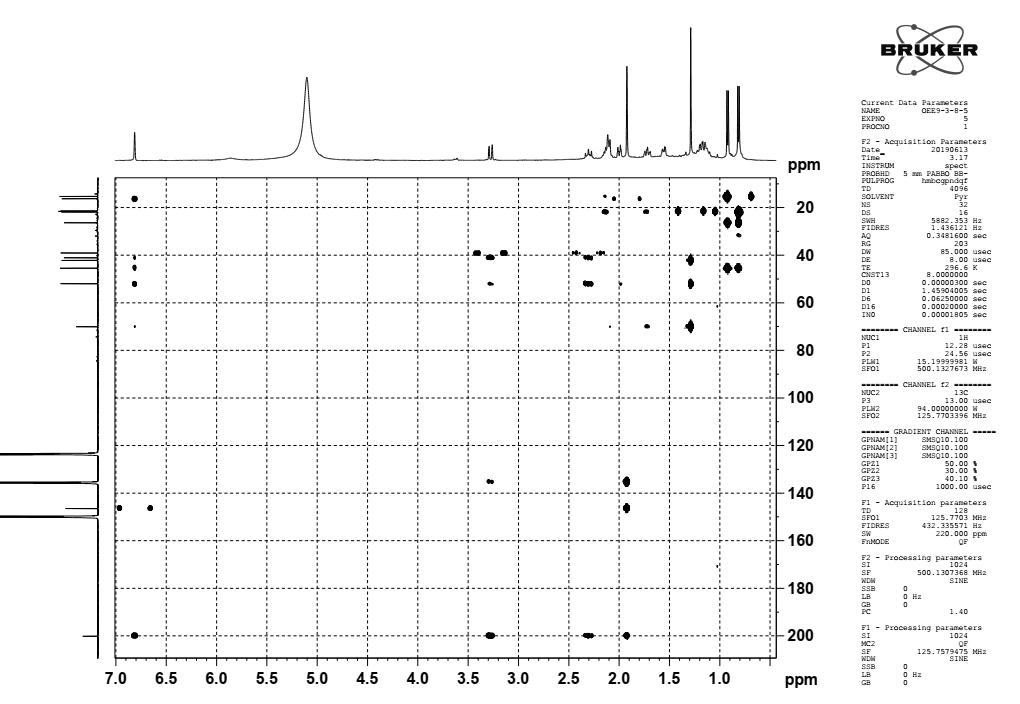


**Figure S56.** HMBC (C_5_D_5_N) spectrum of **6**


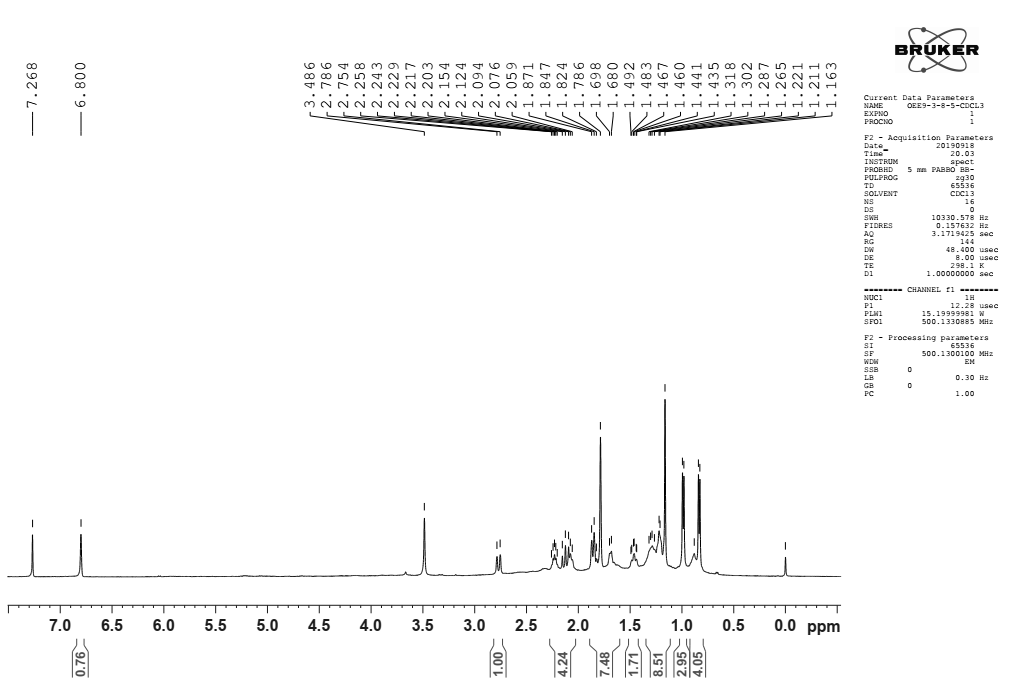


**Figure S57.** ^1^H NMR (500 MHz, CDCl_3_) spectrum of **6**


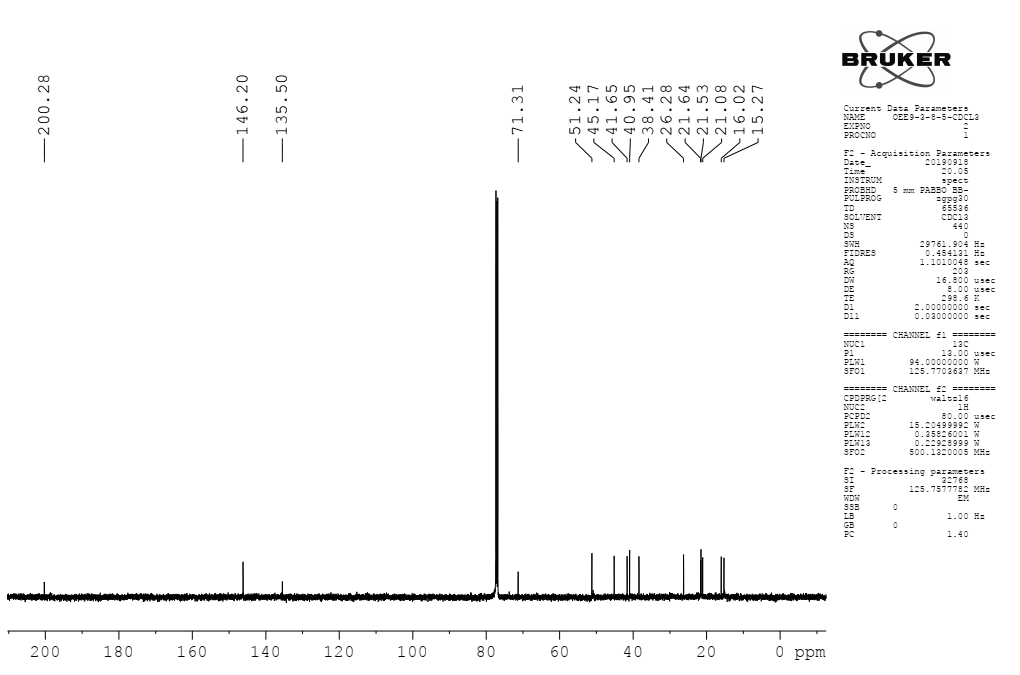


**Figure S58.** ^13^C NMR (125 MHz, CDCl_3_) spectrum of **6**


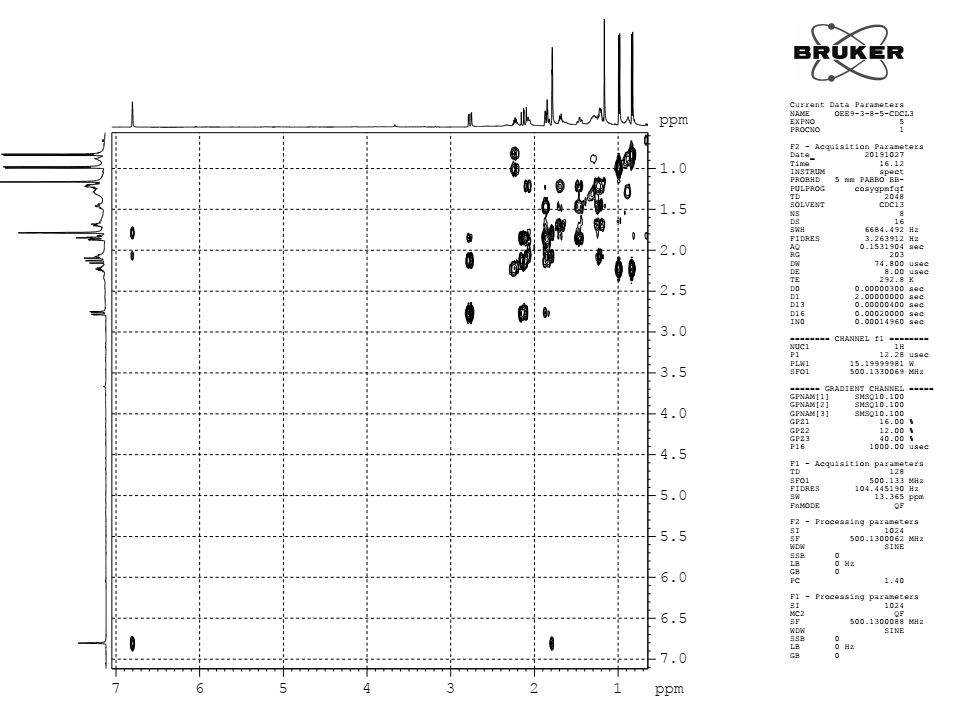


**Figure S59.** ^1^H ^1^H COSY (CDCl_3_) spectrum of **6**


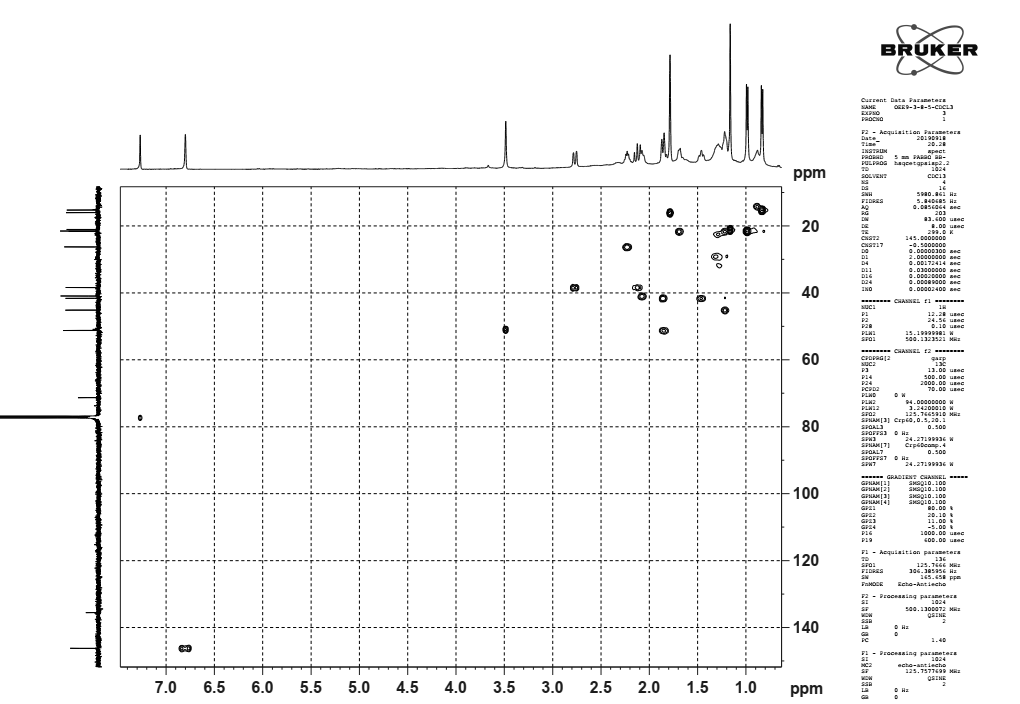


**Figure S60.** HSQC (CDCl_3_) spectrum of **6**


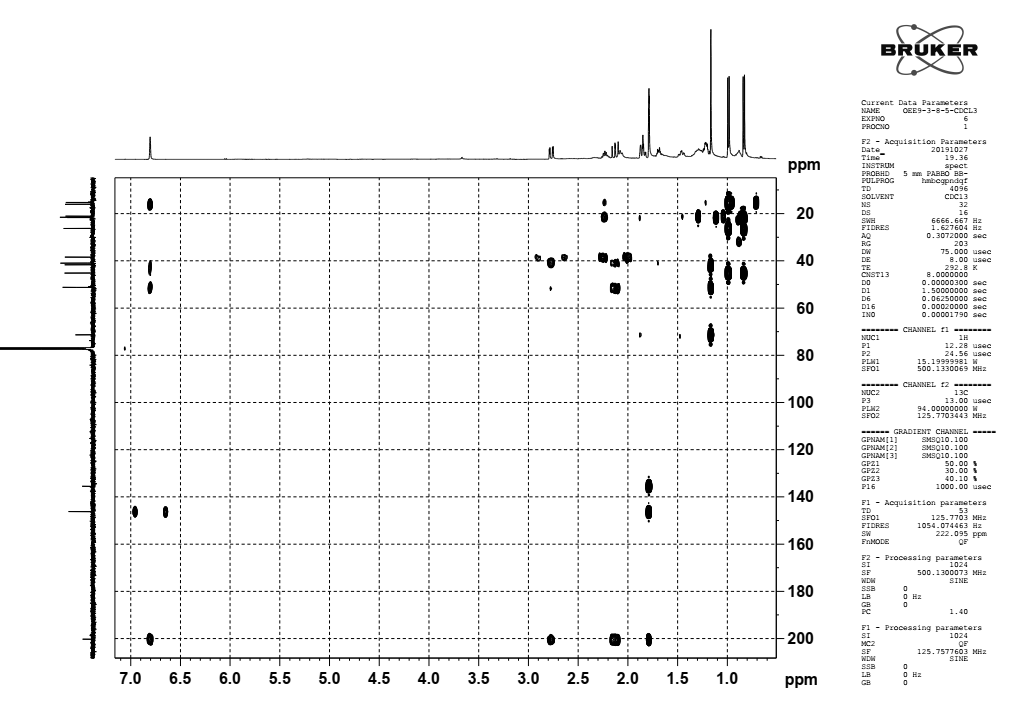


**Figure S61.** HMBC (CDCl_3_) spectrum of **6**


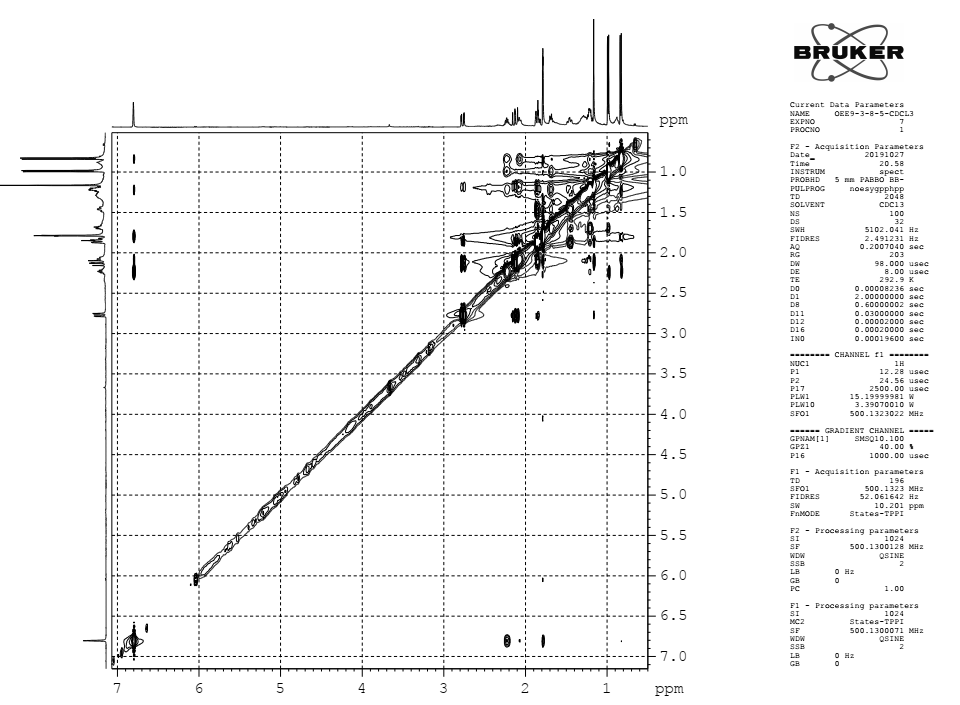


**Figure S62.** NOESY (CDCl_3_) spectrum of **6**


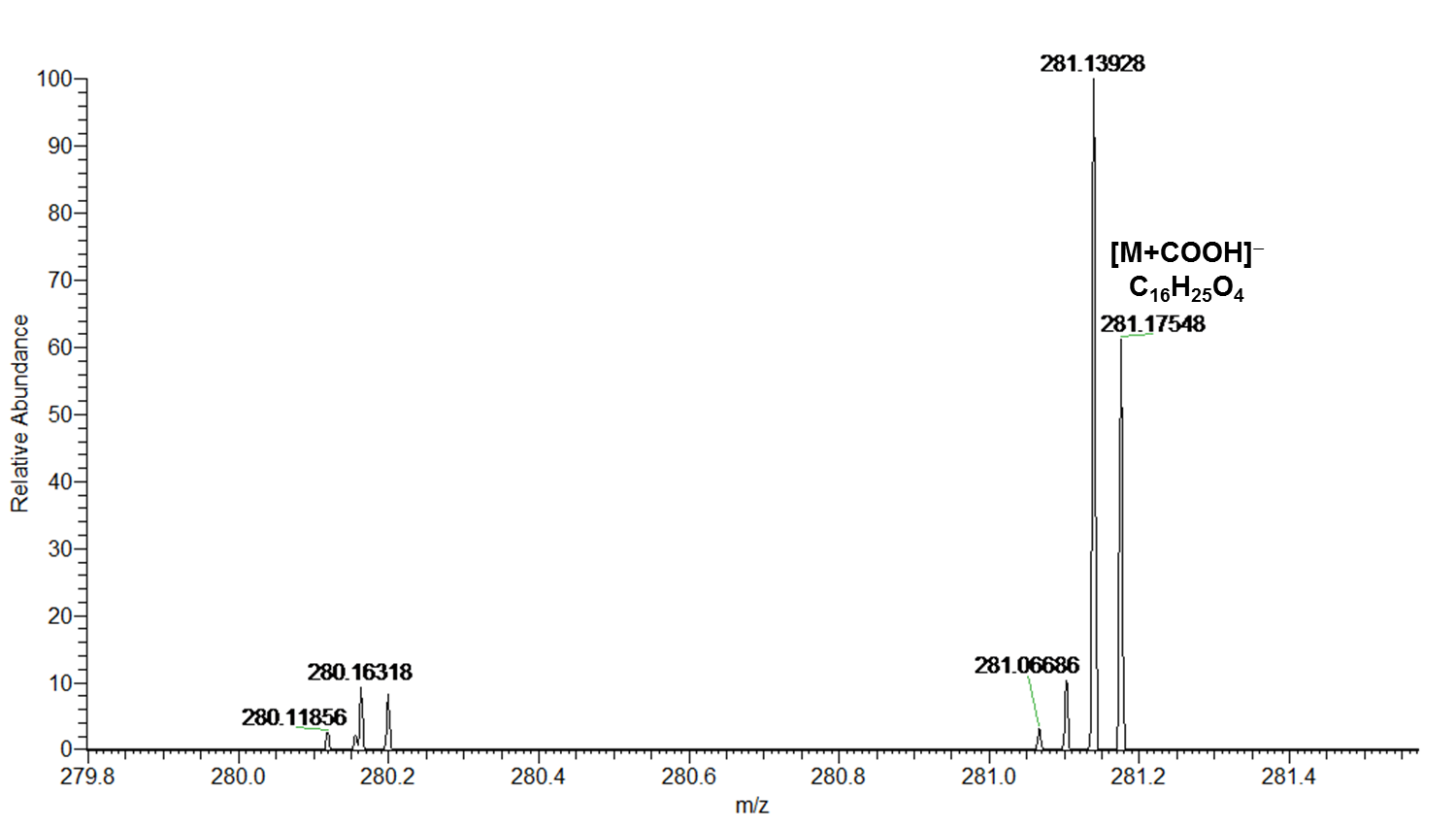


**Figure S63.** ESI-Q-Orbitrap-MS spectrum of **6**


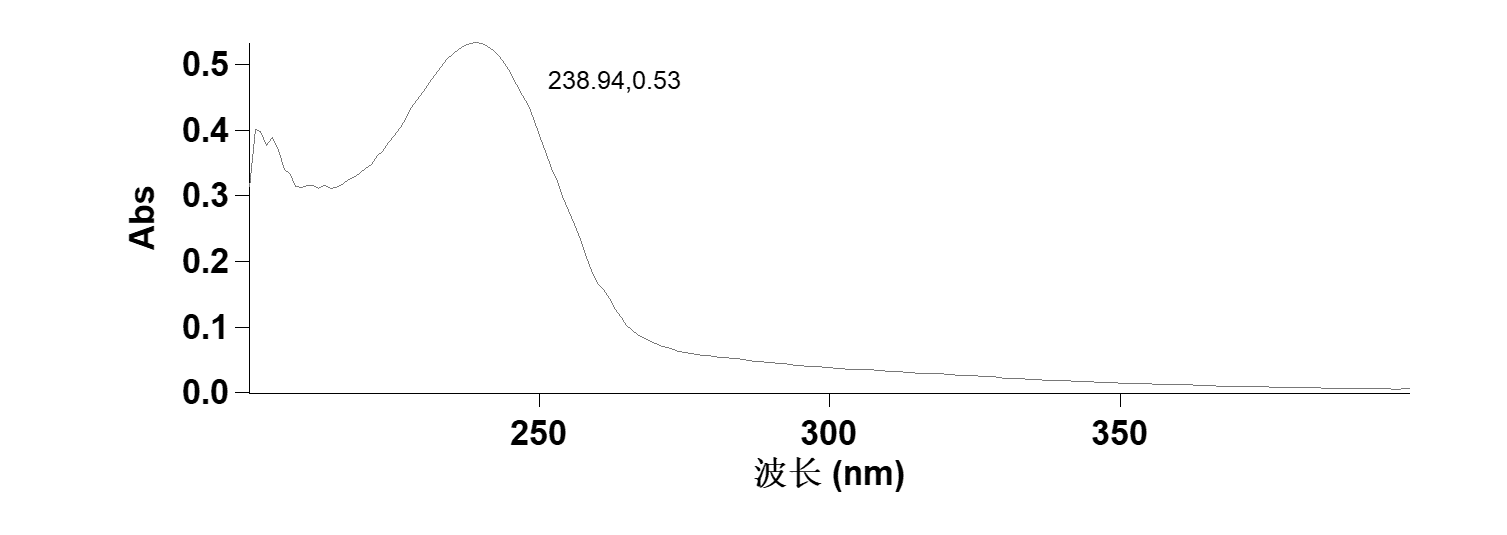


**Figure S64.** UV spectrum of **6**

**Figure S65.** IR spectrum of **6**


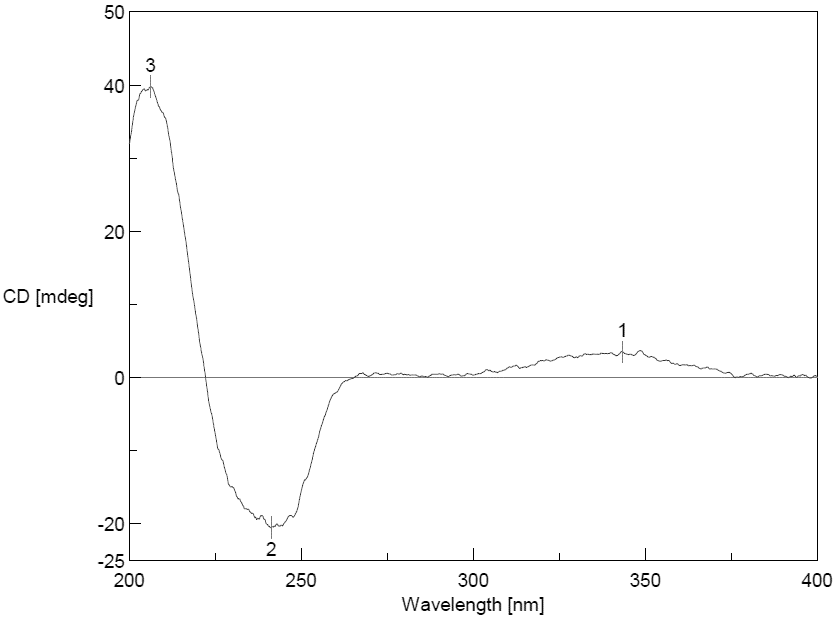


**Figure S66.** CD spectrum of **6**


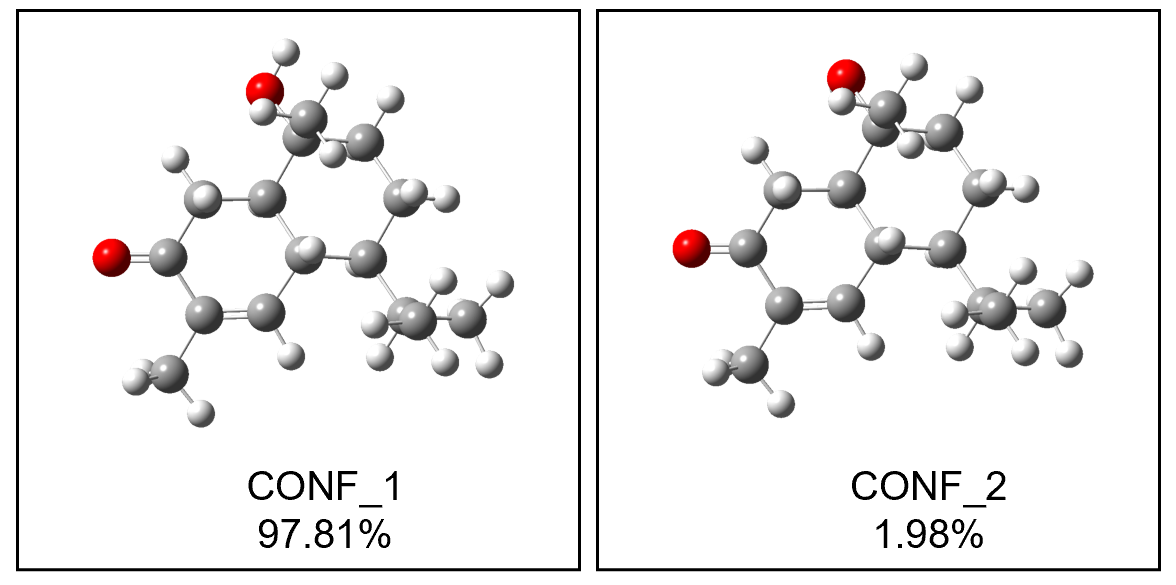


**Figure S67.** The conformational analysis and optimized geometries of predominant conformers of **6**


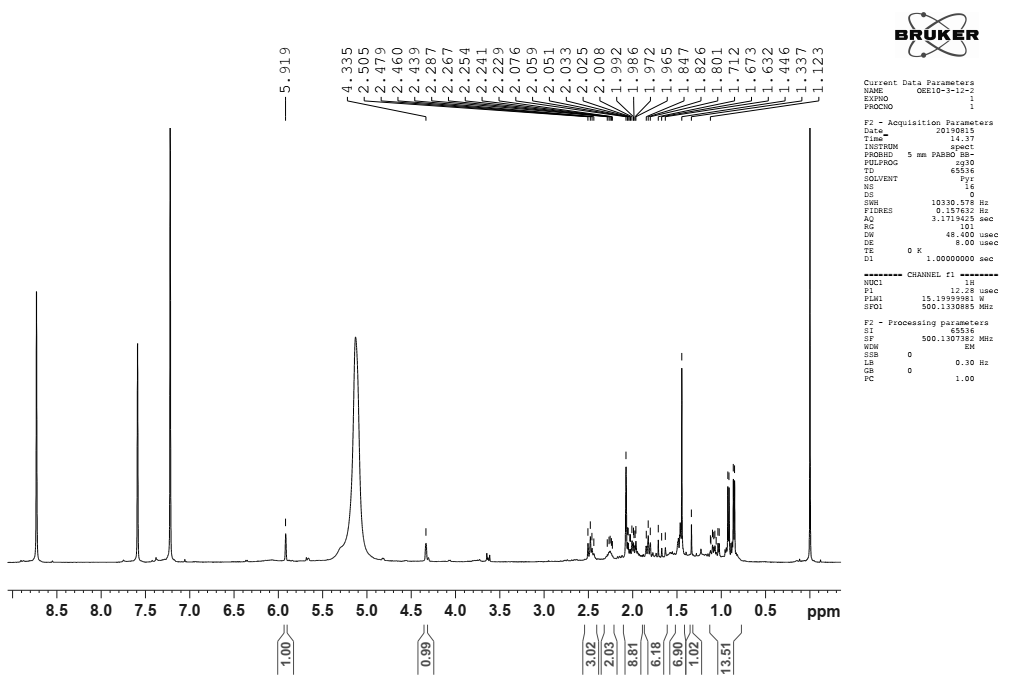


**Figure S68.** ^1^H NMR (500 MHz, C_5_D_5_N) spectrum of **7**


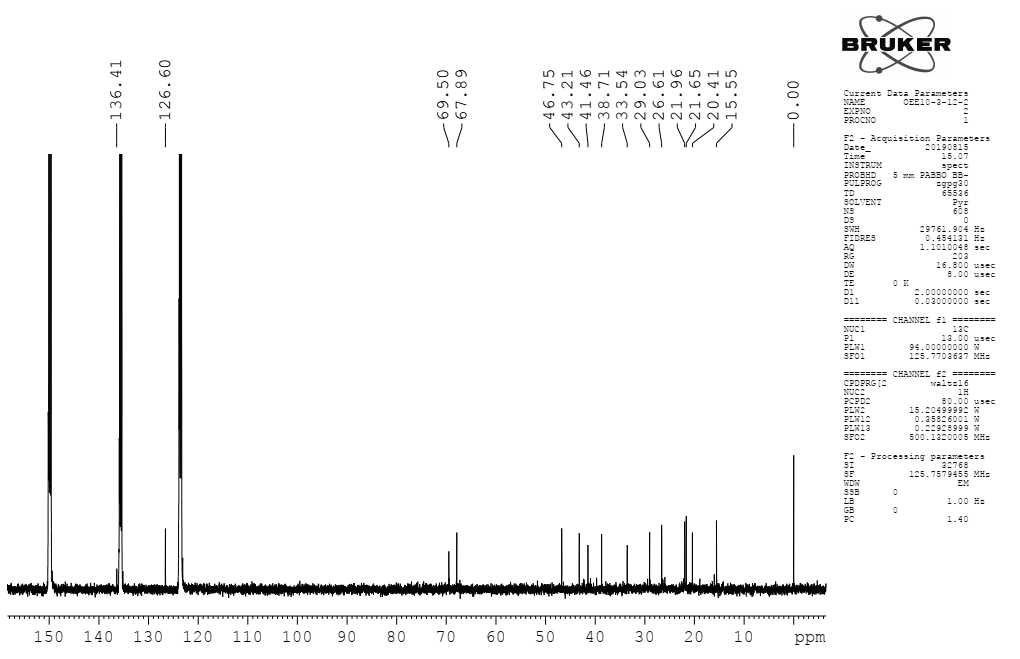


**Figure S69.** ^13^C NMR (125 MHz, C_5_D_5_N) spectrum of **7**


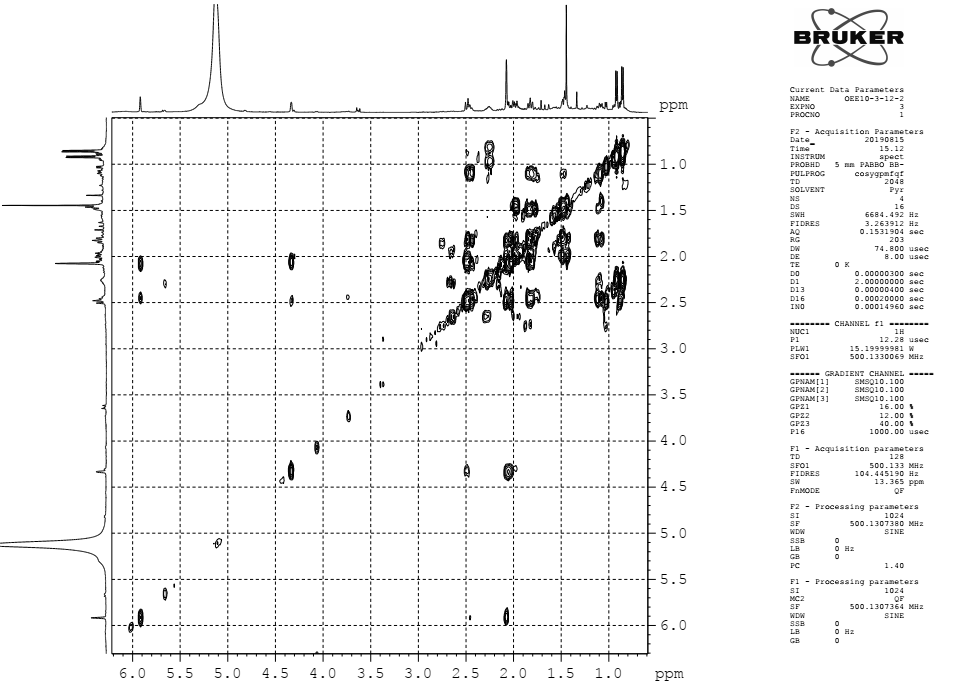


**Figure S70.** ^1^H ^1^H COSY (C_5_D_5_N) spectrum of **7**


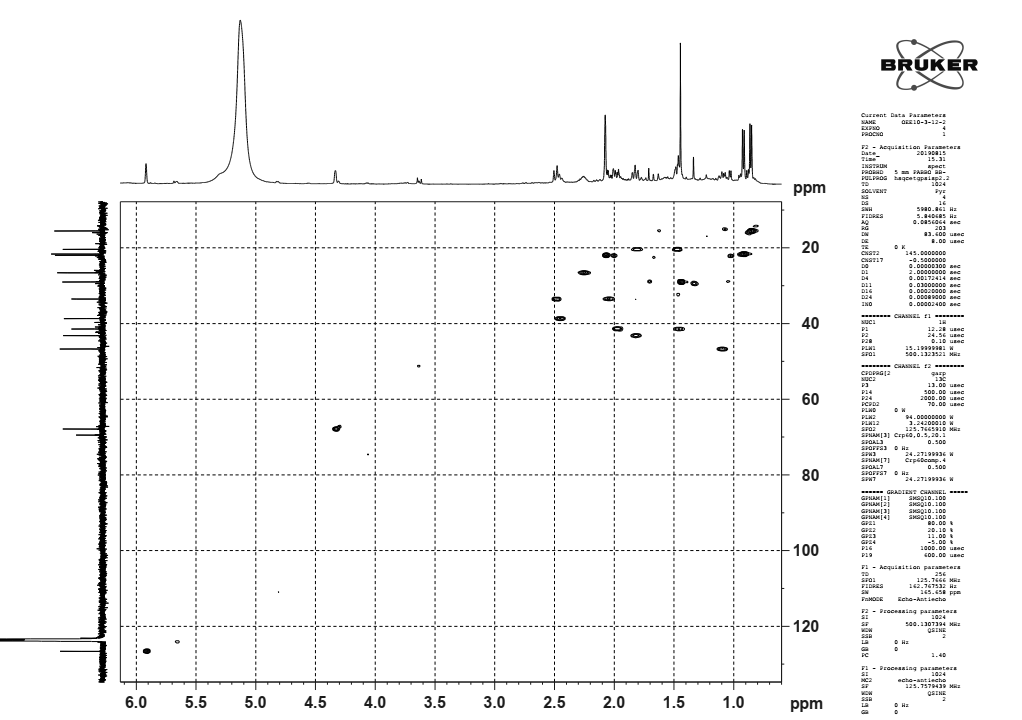


**Figure S71.** HSQC (C_5_D_5_N) spectrum of **7**


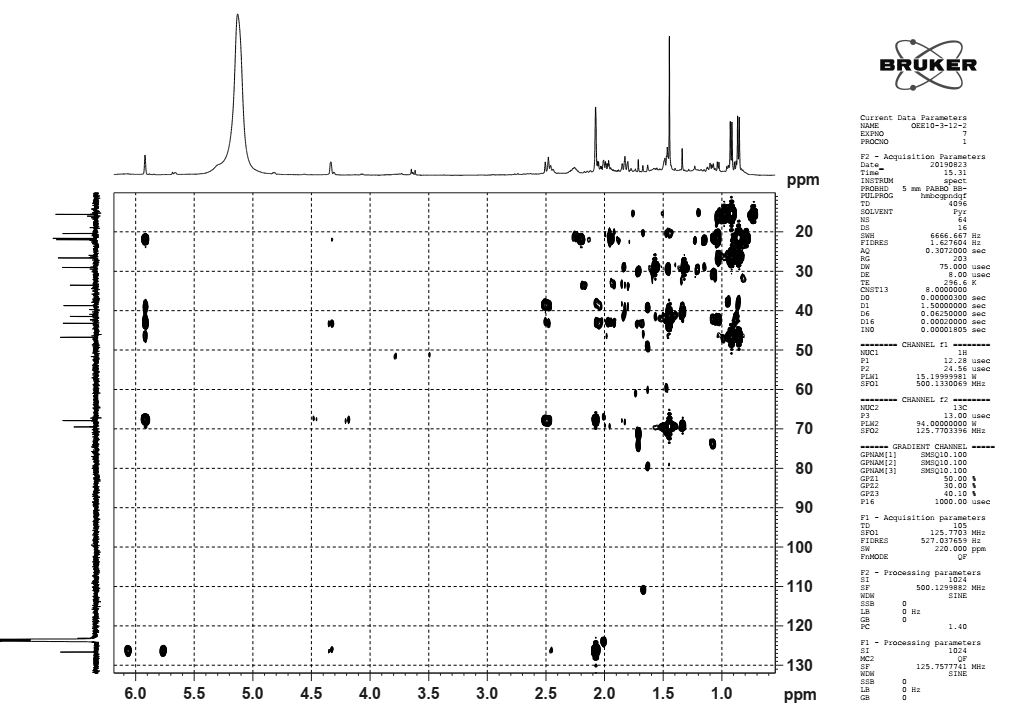


**Figure S72.** MBC (C_5_D_5_N) spectrum of **7**


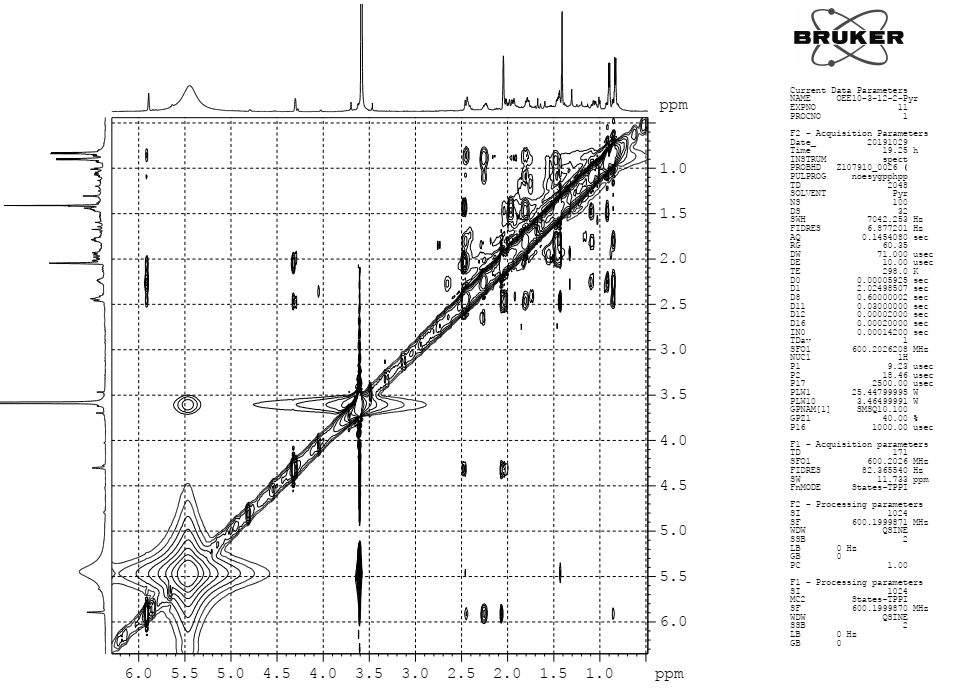


**Figure S73.** NOESY (C_5_D_5_N) spectrum of **7**


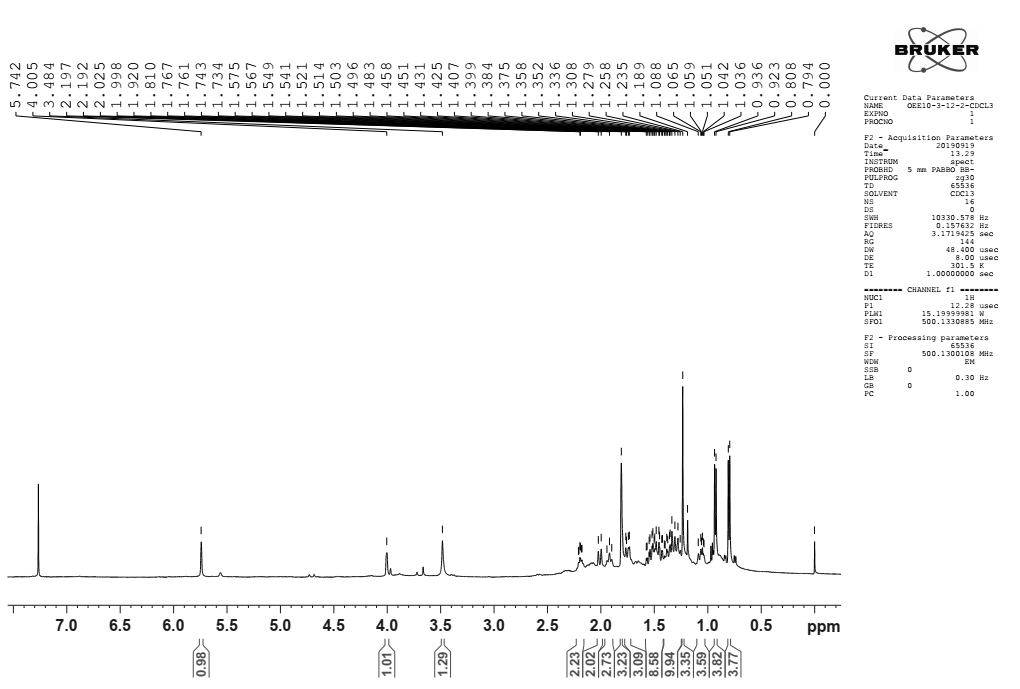


**Figure S74.** ^1^H NMR (500 MHz, CDCl_3_) spectrum of **7**


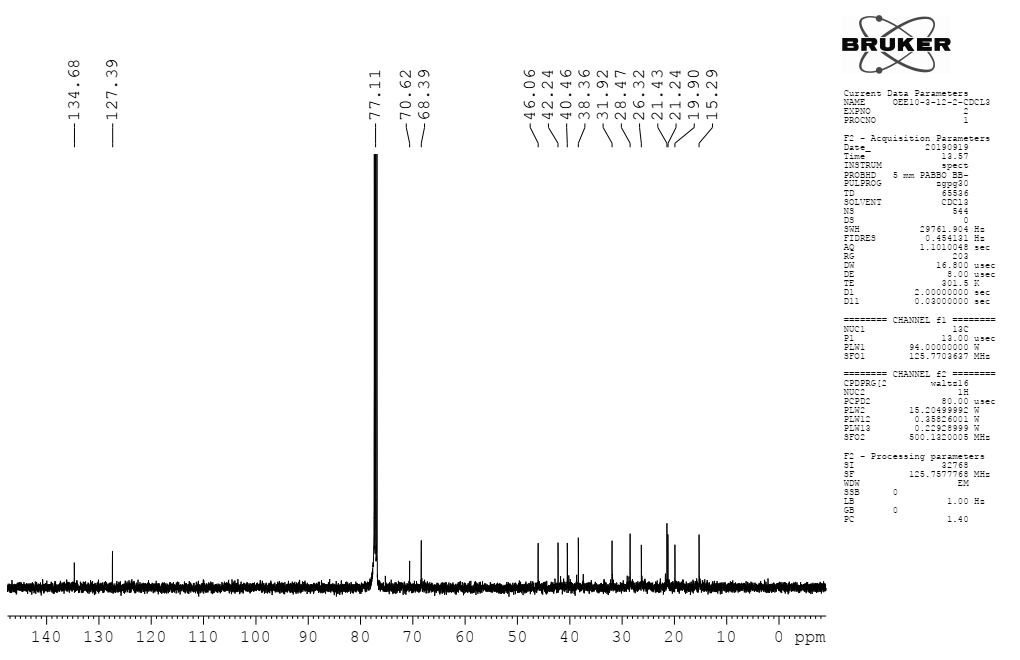


**Figure S75.** ^13^C NMR (125 MHz, CDCl_3_) spectrum of **7**


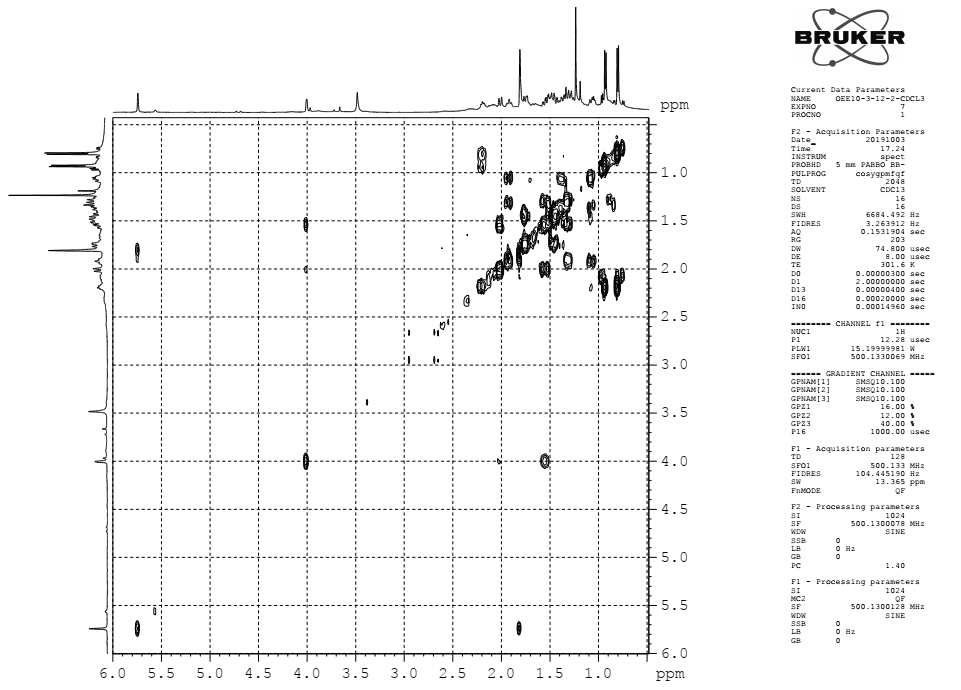


**Figure S76.** ^1^H ^1^H COSY (CDCl_3_) spectrum of **7**


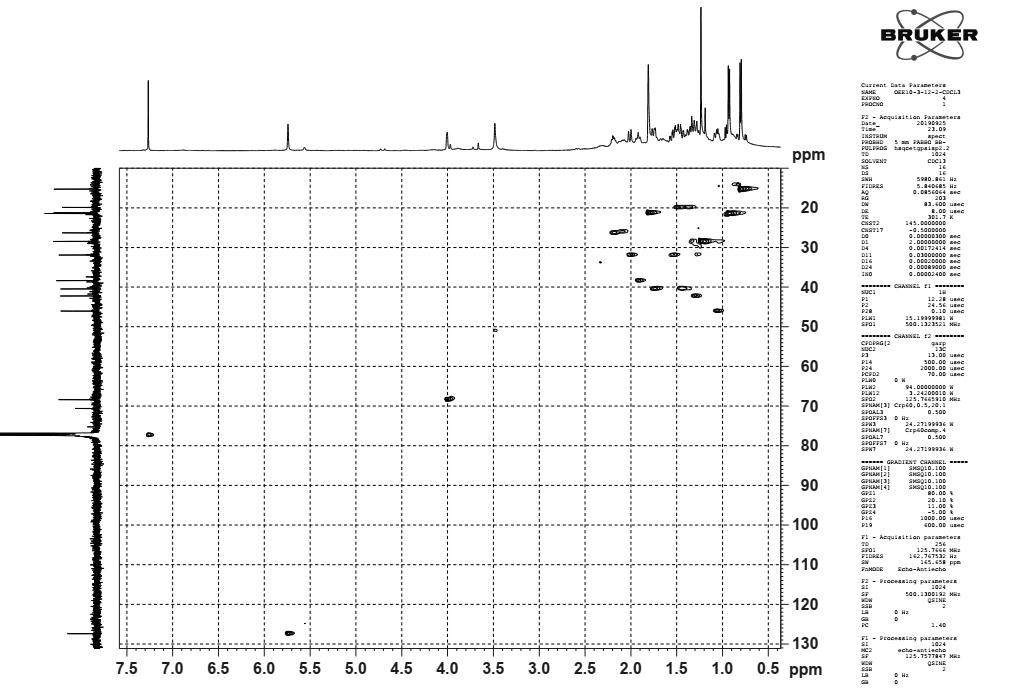


**Figure S77.** HSQC (CDCl_3_) spectrum of **7**


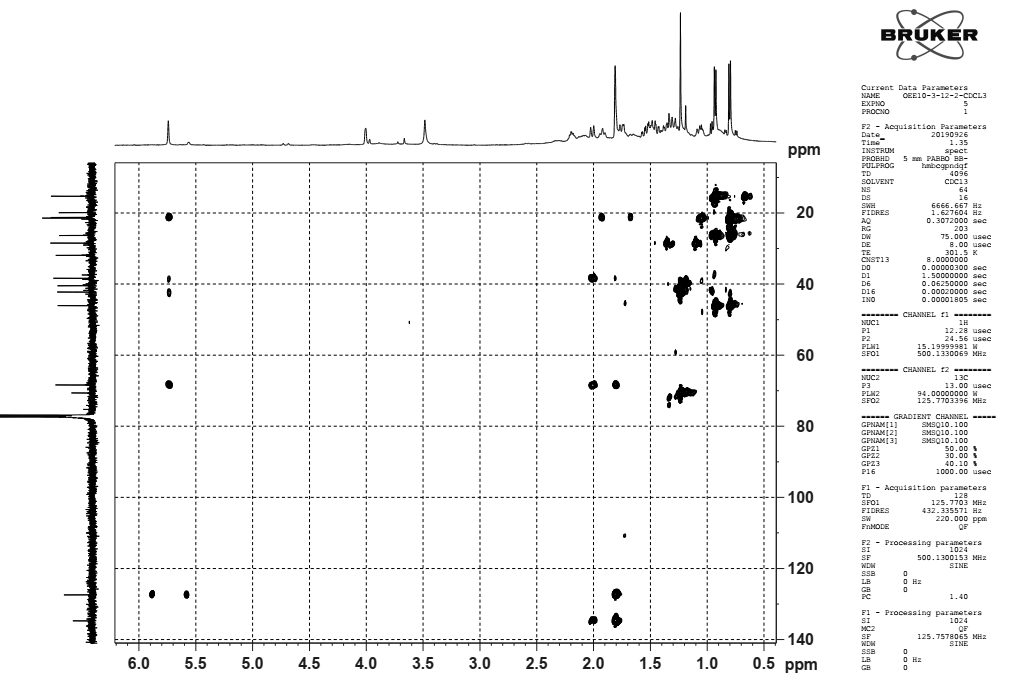


**Figure S78.** HMBC (CDCl_3_) spectrum of **7**


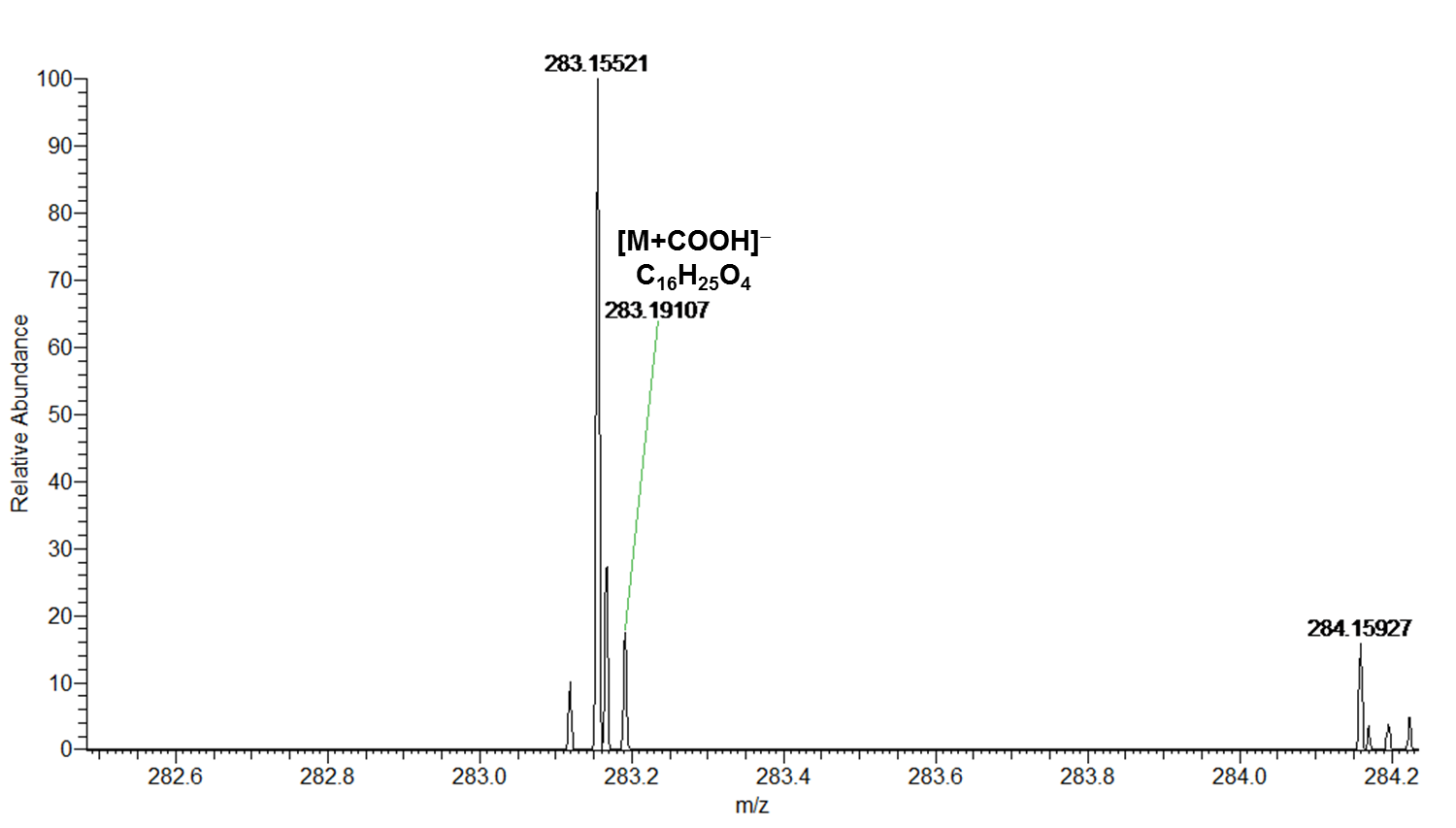


**Figure S79.** ESI-Q-Orbitrap-MS spectrum of **7**

**Figure S80.** IR spectrum of **7**


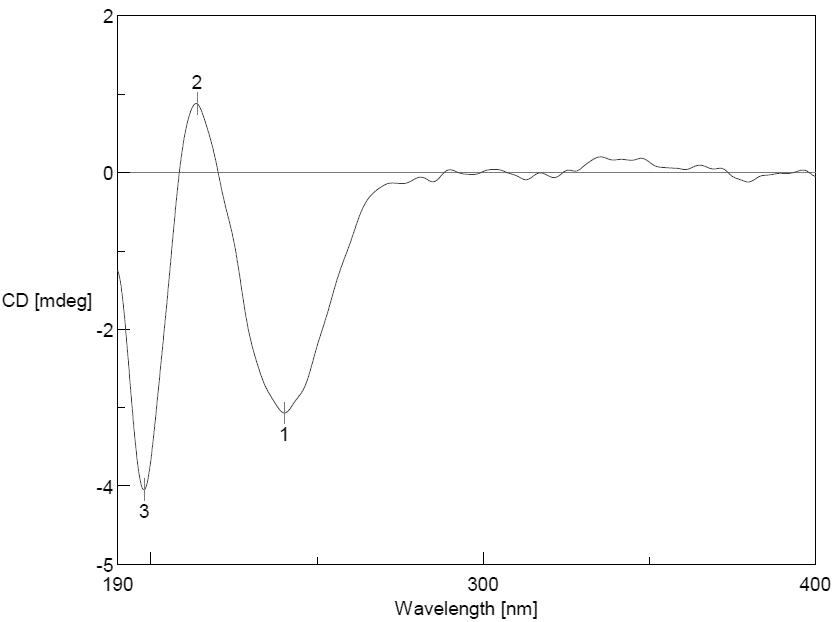


**Figure S81.** CD spectrum of **7**


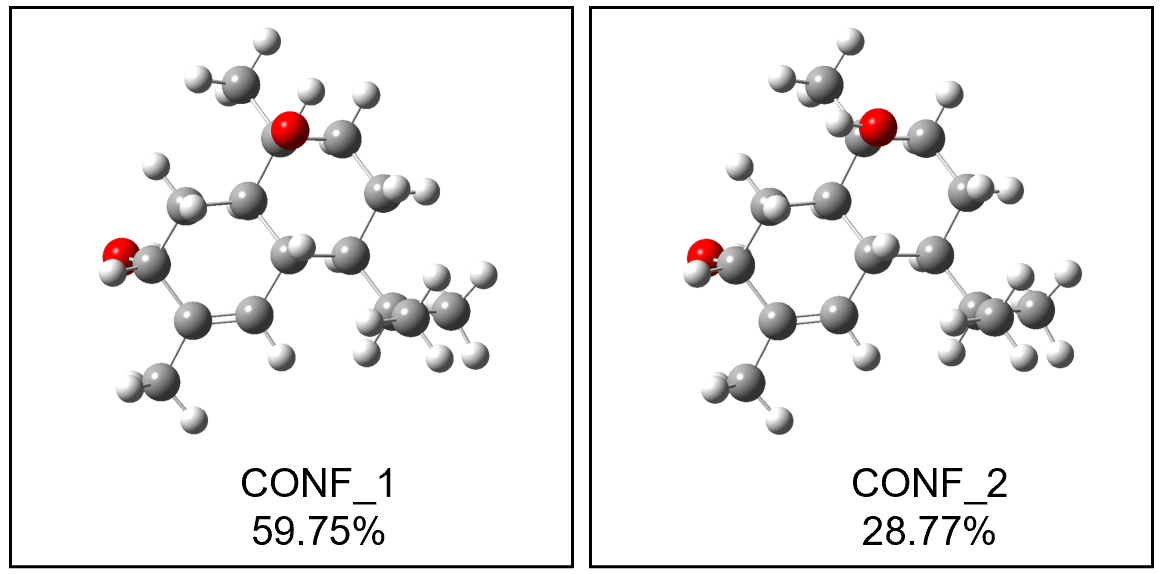


**Figure S82.** The conformational analysis and optimized geometries of predominant conformers of **7**


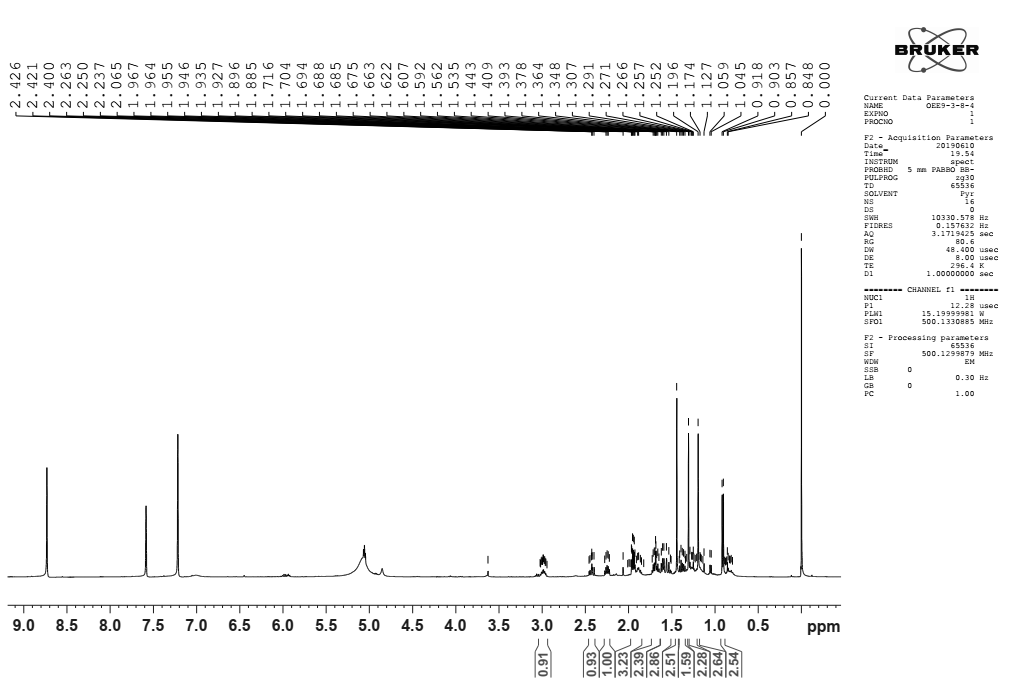


**Figure S83.** ^1^H NMR (500 MHz, C_5_D_5_N) spectrum of **8**


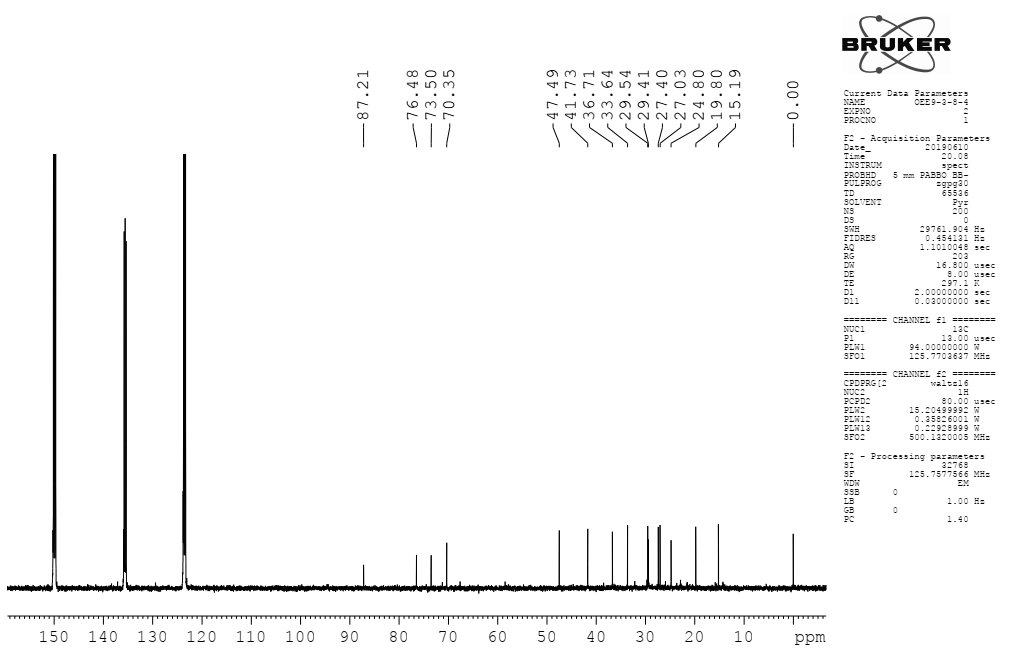


**Figure S84.** ^13^C NMR (125 MHz, C_5_D_5_N) spectrum of **8**


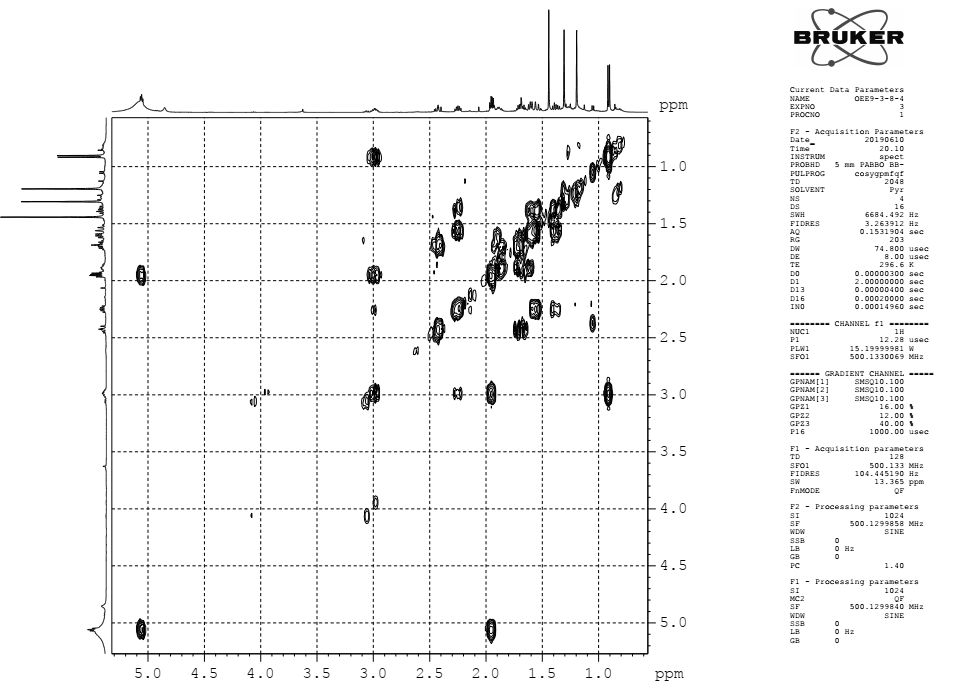


**Figure S85.** ^1^H ^1^H COSY (C_5_D_5_N) spectrum of **8**


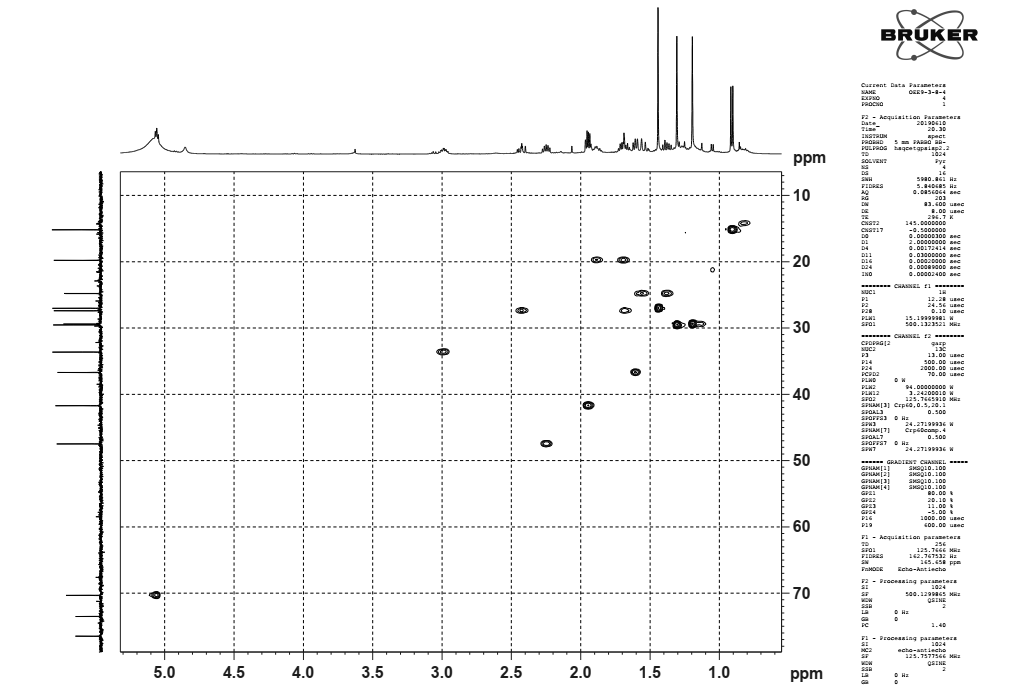


**Figure S86.** HSQC (C_5_D_5_N) spectrum of **8**


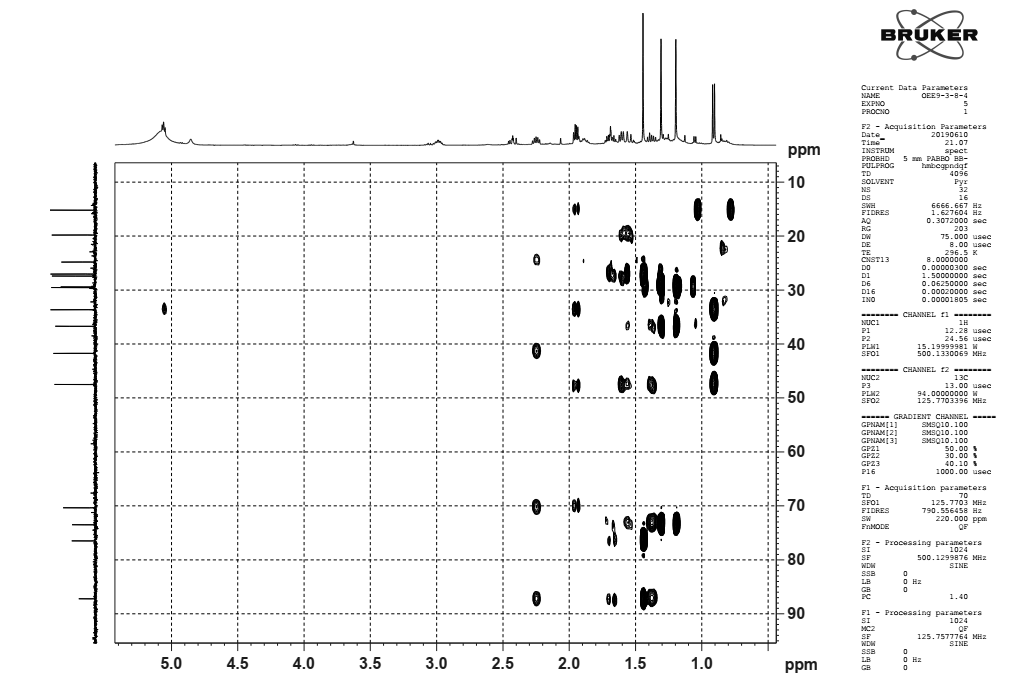


**Figure S87.** HMBC (C_5_D_5_N) spectrum of **8**


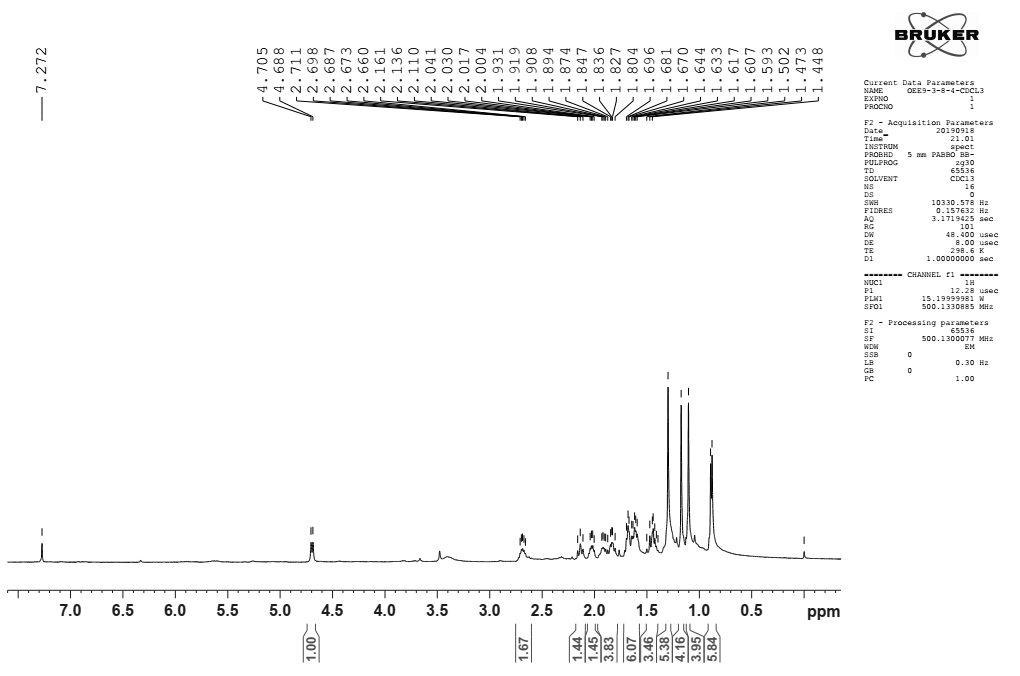


**Figure S88.** ^1^H NMR (500 MHz, CDCl_3_) spectrum of **8**


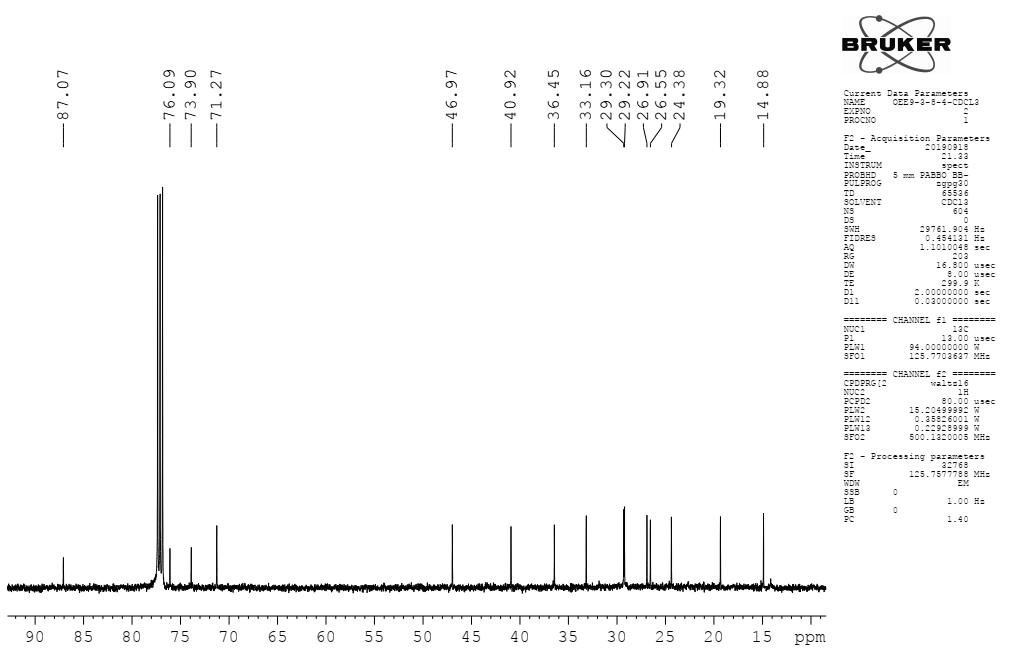


**Figure S89.** ^13^C NMR (125 MHz, CDCl_3_) spectrum of **8**


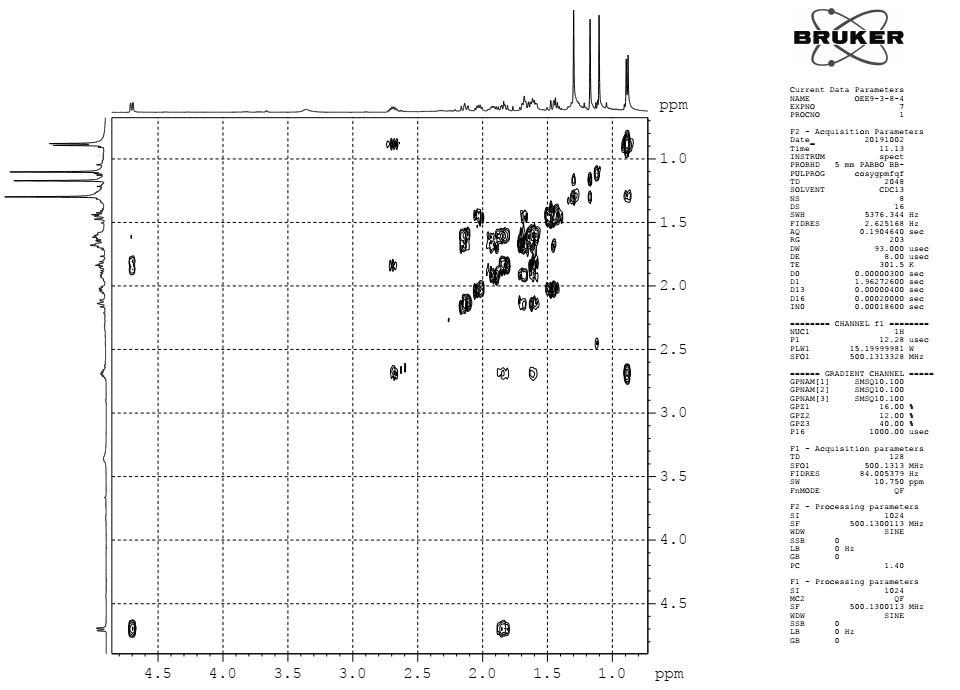


**Figure S90.** ^1^H ^1^H COSY (CDCl_3_) spectrum of **8**


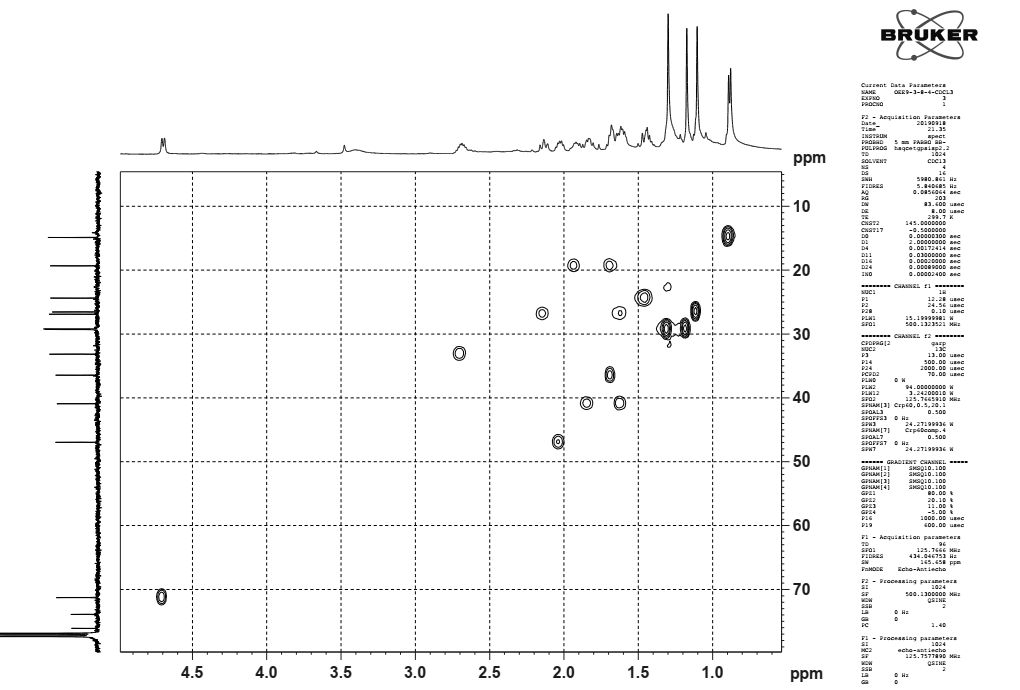


**Figure S91.** HSQC (CDCl_3_) spectrum of **8**


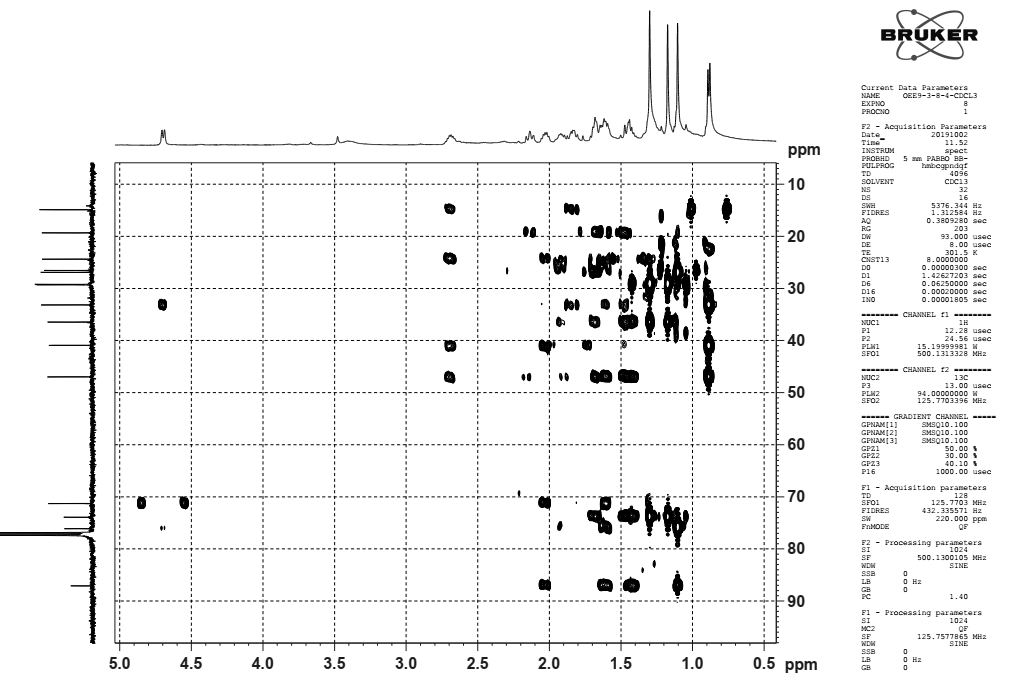


**Figure S92.** HMBC (CDCl_3_) spectrum of **8**


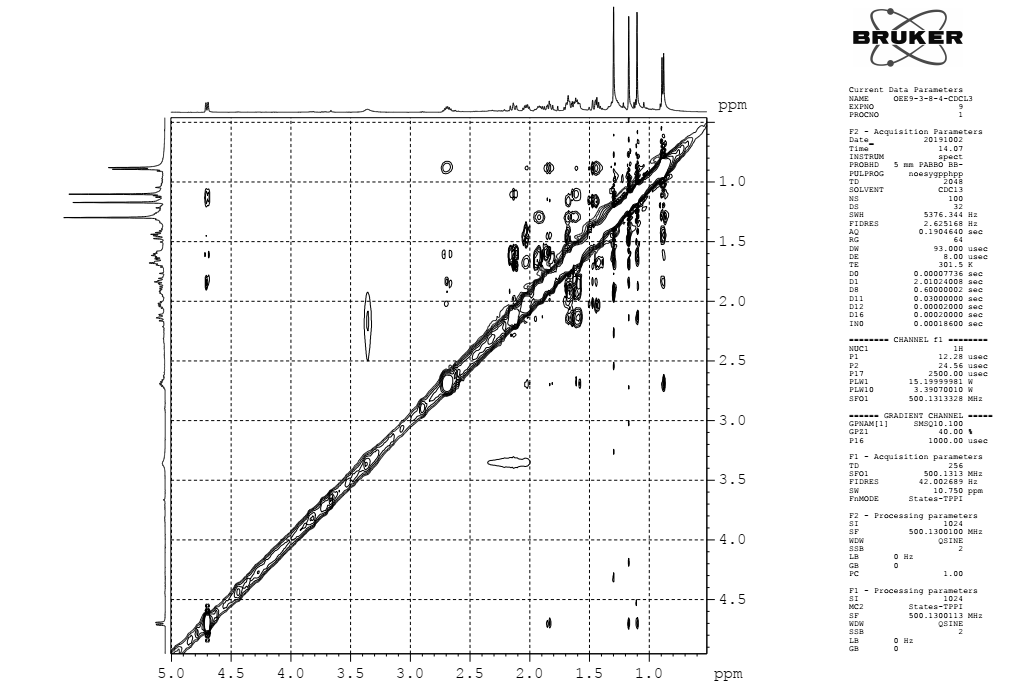


**Figure S91.** NOESY (CDCl_3_) spectrum of **8**


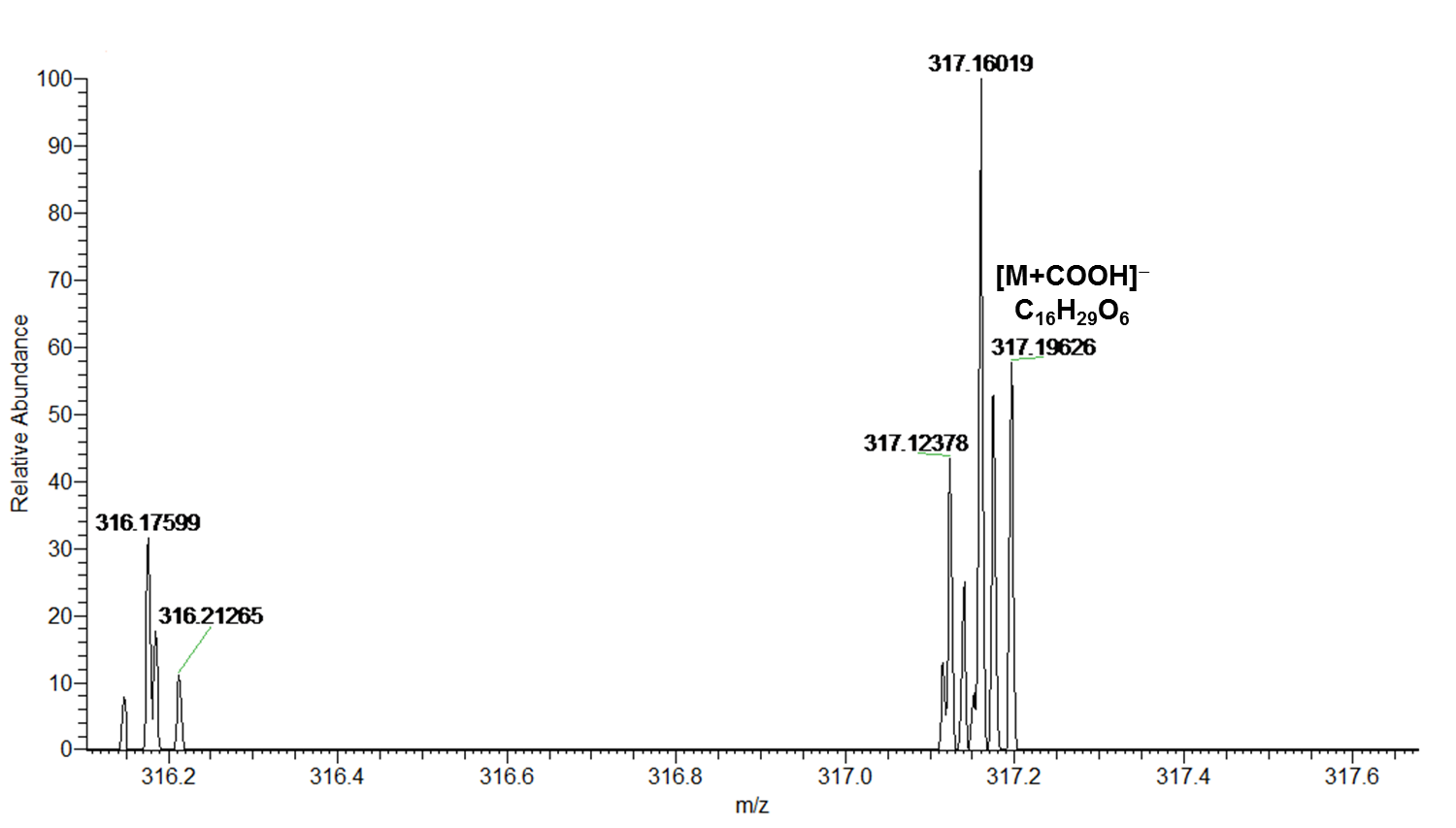


**Figure S94.** ESI-Q-Orbitrap-MS spectrum of **8**

**Figure S95.** IR spectrum of **8**


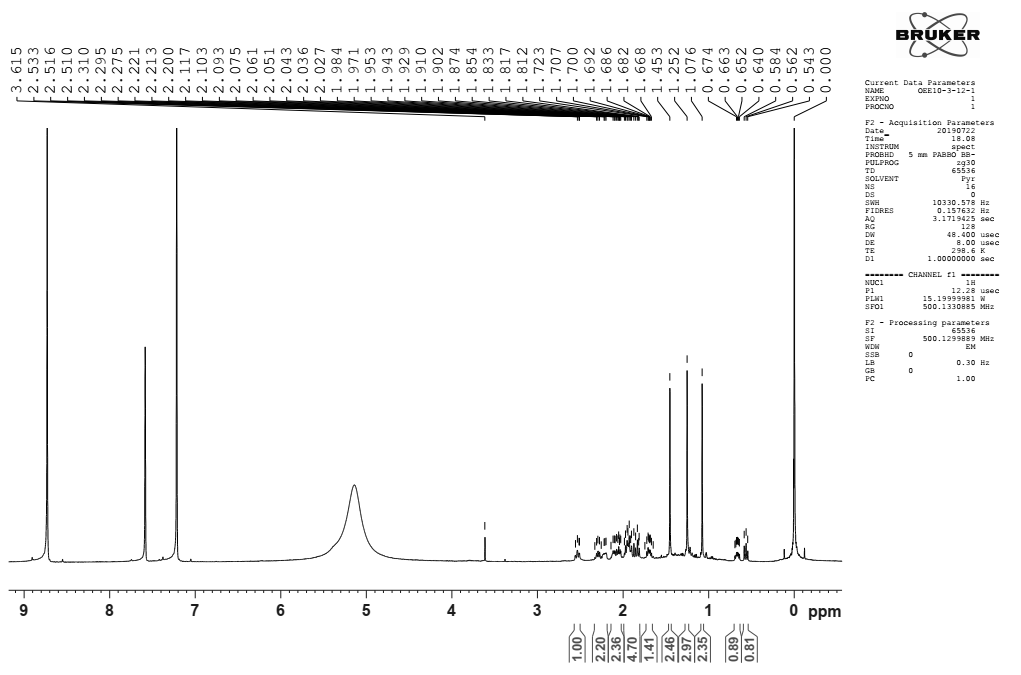


**Figure S96.** ^1^H NMR (500 MHz, C_5_D_5_N) spectrum of **9**


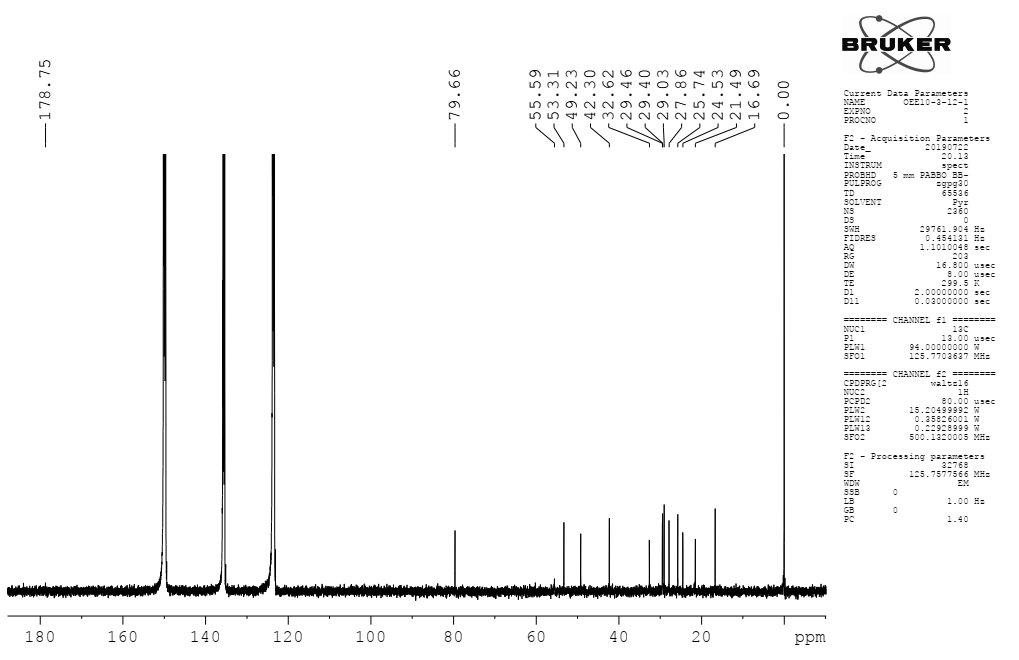


**Figure S97.** ^13^C NMR (125 MHz, C_5_D_5_N) spectrum of **9**


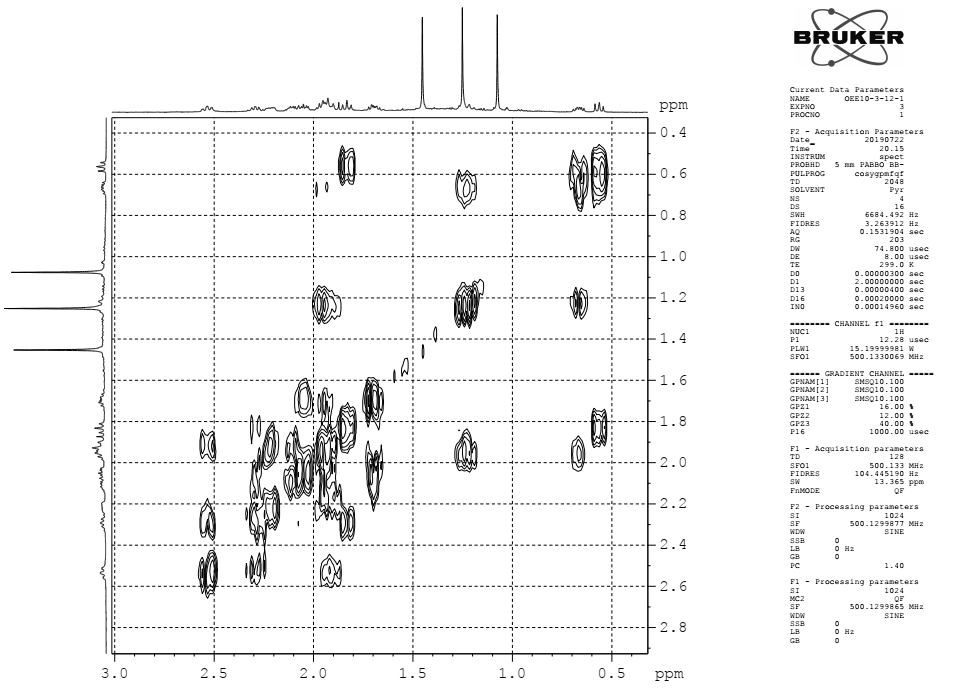


**Figure S98.** ^1^H ^1^H COSY (C_5_D_5_N) spectrum of **9**


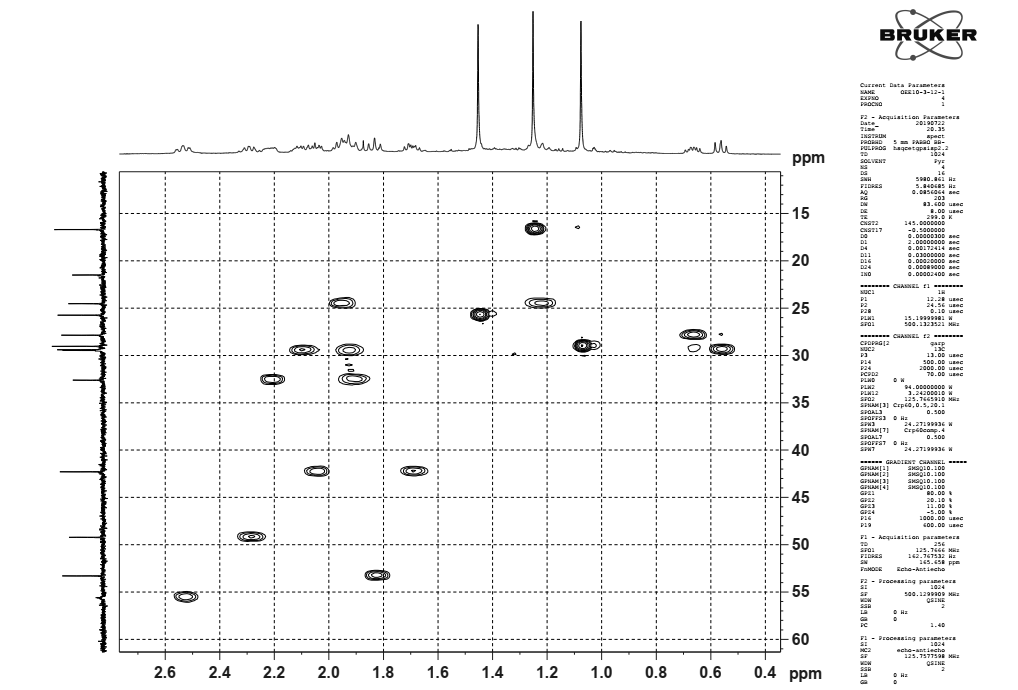


**Figure S99.** HSQC (C_5_D_5_N) spectrum of **9**


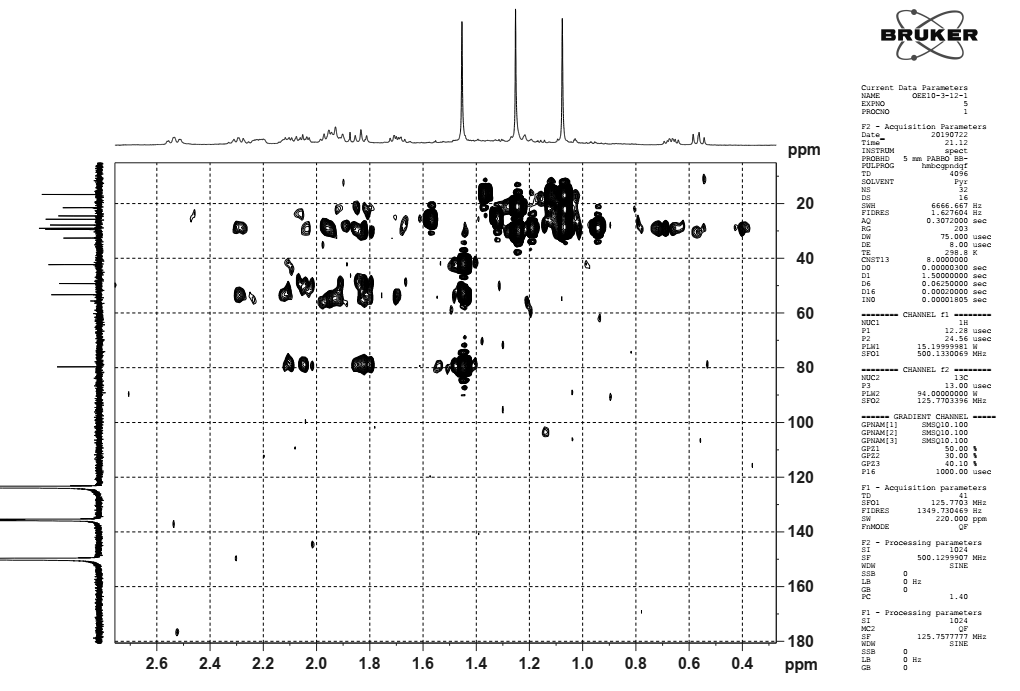


**Figure S100.** HMBC (C_5_D_5_N) spectrum of **9**


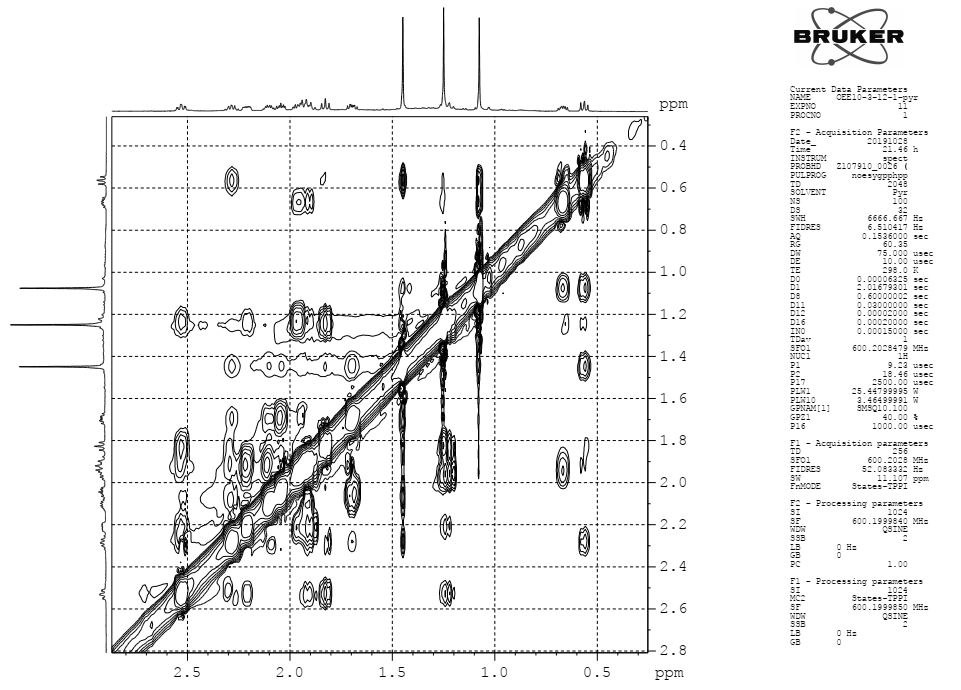


**Figure S101.** NOESY (C_5_D_5_N) spectrum of **9**


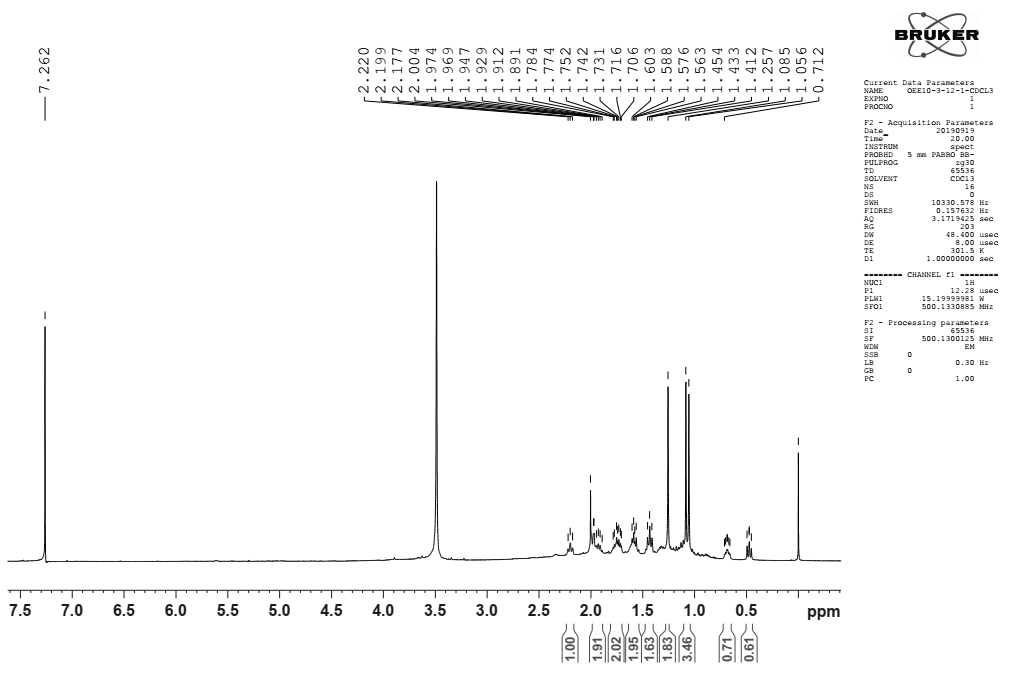


**Figure S102.** ^1^H NMR (500 MHz, CDCl_3_) spectrum of **9**


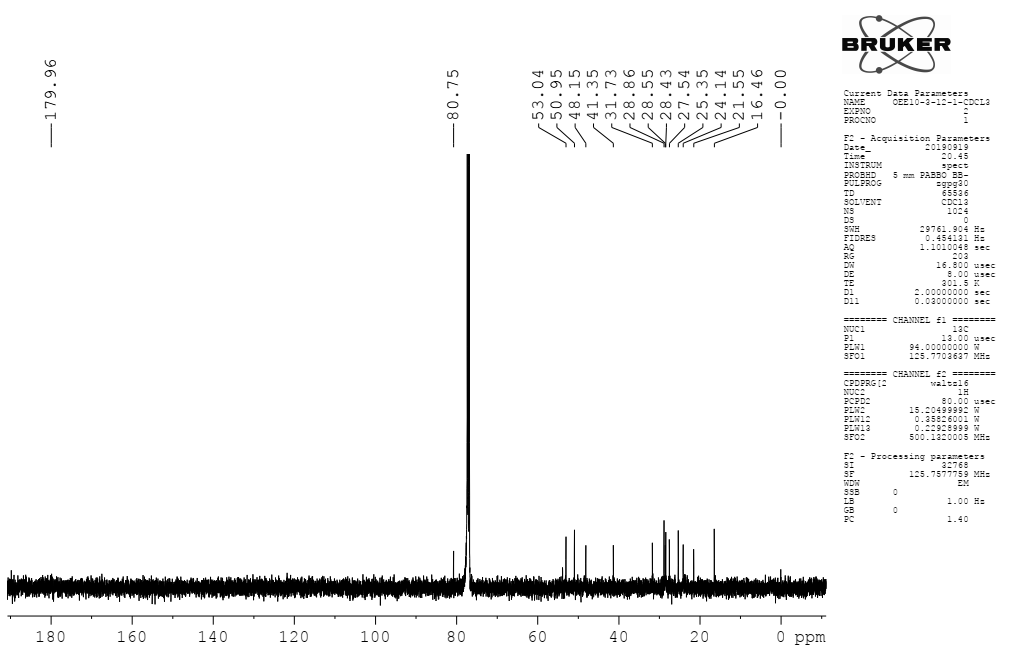


**Figure S103.** ^13^C NMR (125 MHz, CDCl_3_) spectrum of **9**


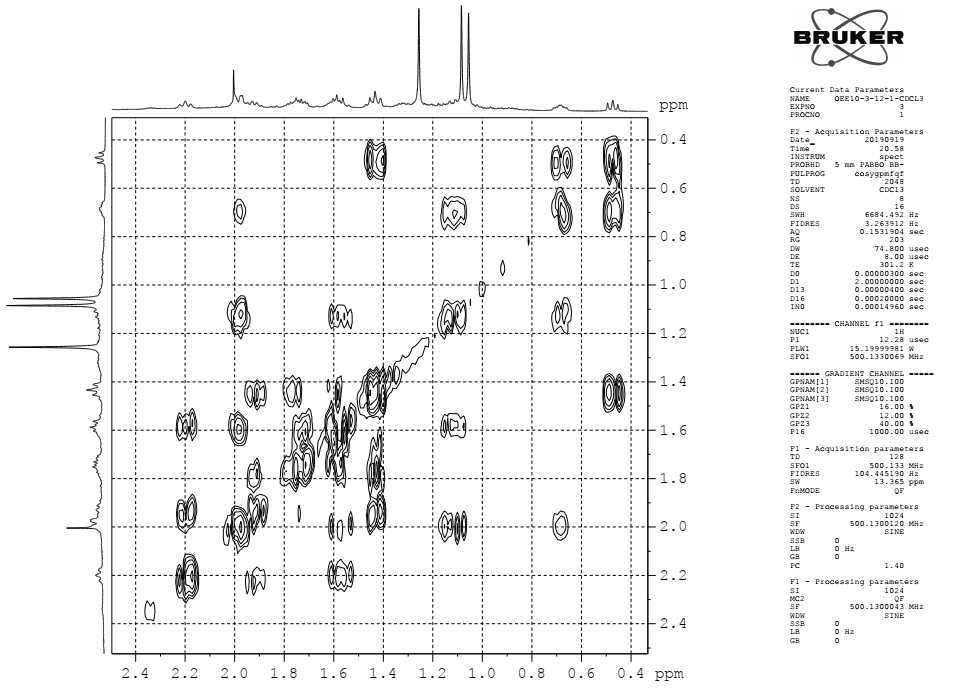


**Figure S104.** ^1^H ^1^H COSY (CDCl_3_) spectrum of **9**


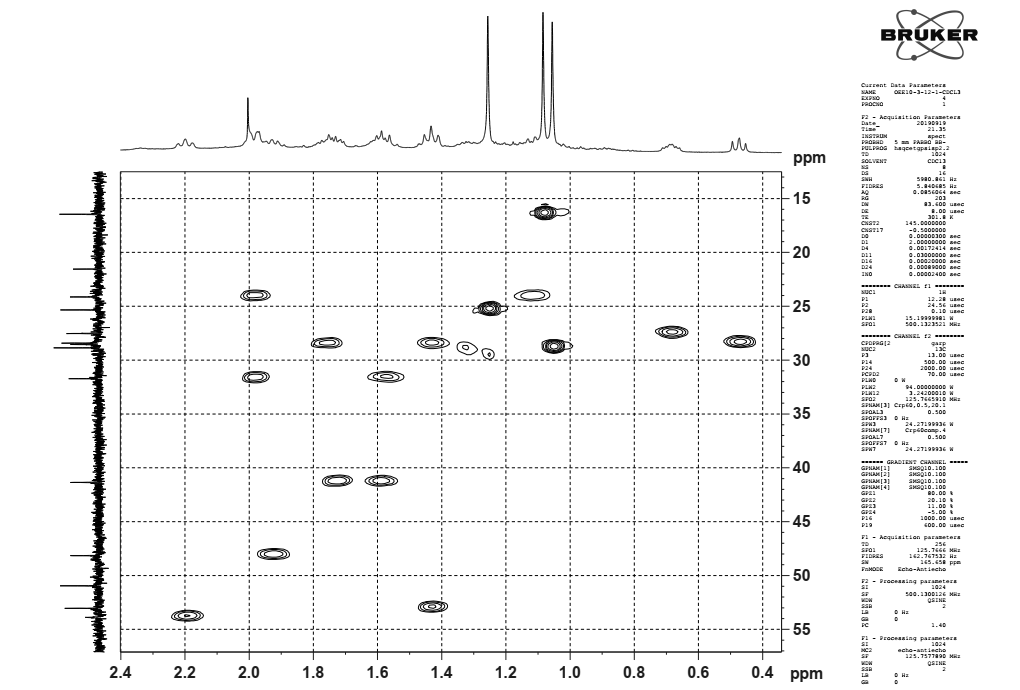


**Figure S105.** HSQC (CDCl_3_) spectrum of **9**


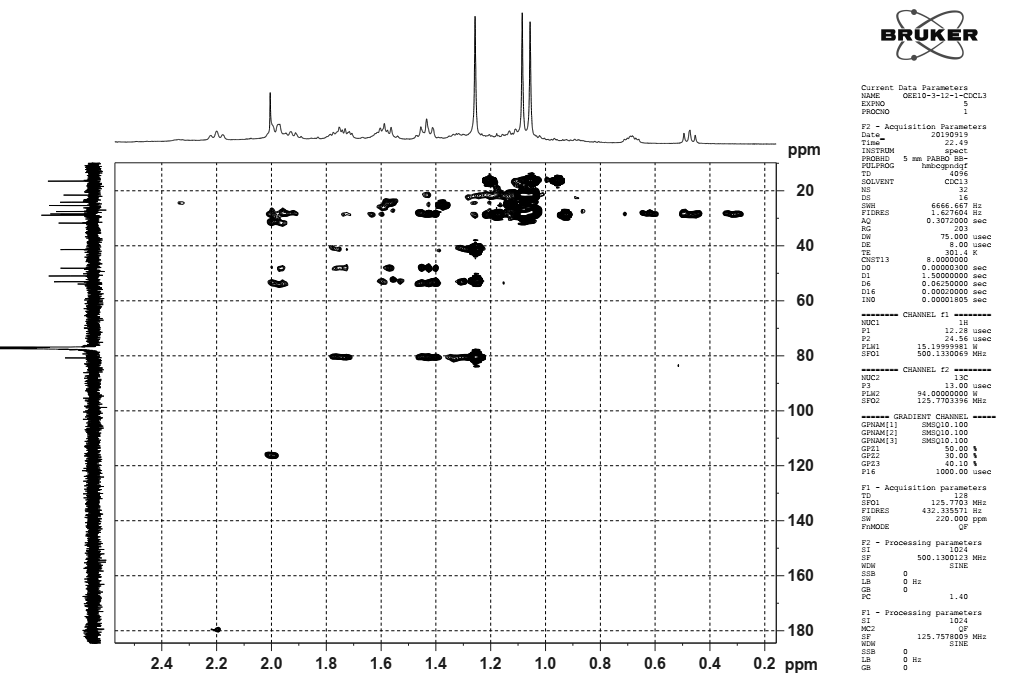


**Figure S106.** HMBC (CDCl_3_) spectrum of **9**


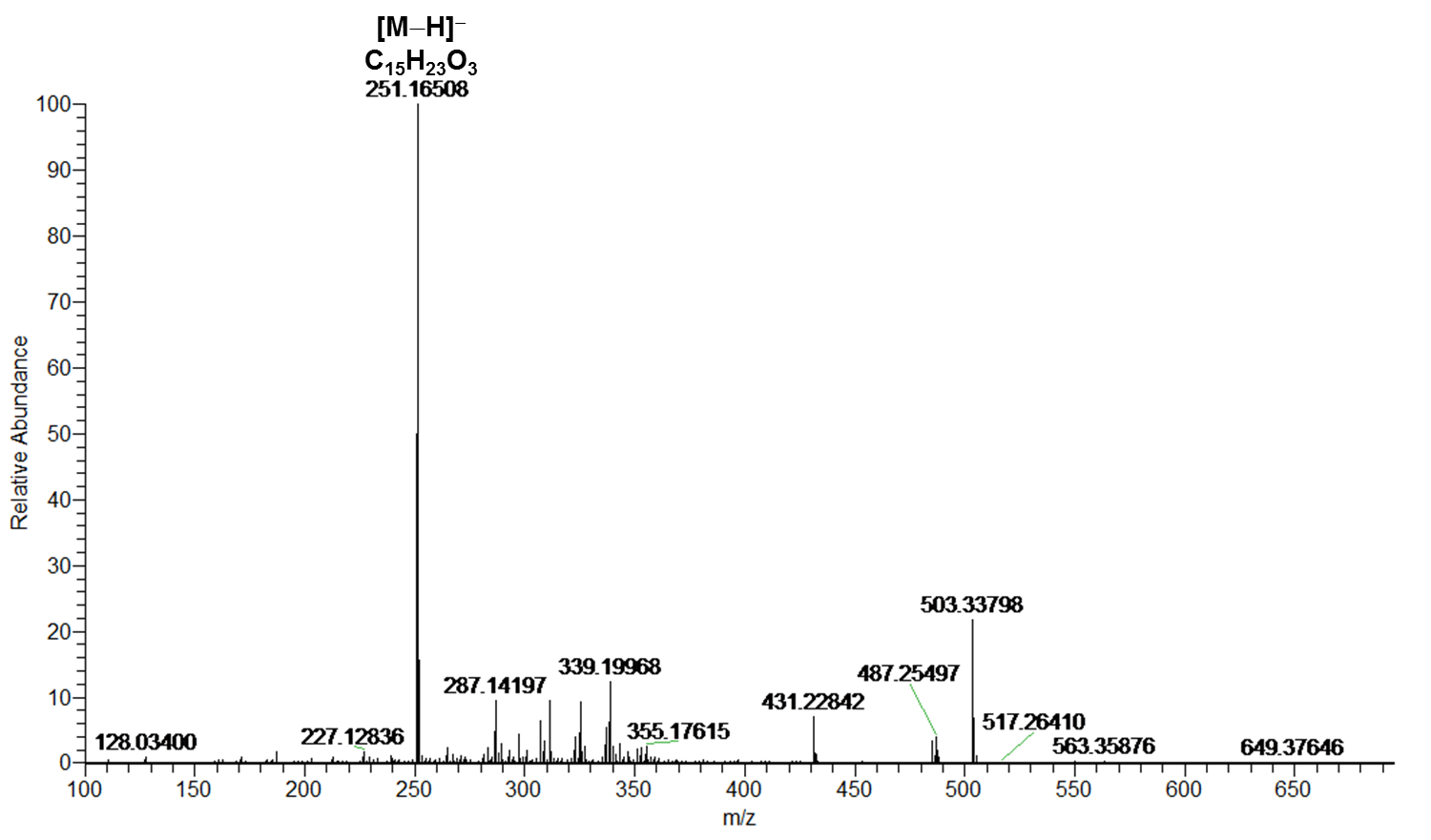


**Figure S107.** ESI-Q-Orbitrap-MS spectrum of **9**

**Figure S108.** ESI-Q-Orbitrap-MS spectrum of **9**


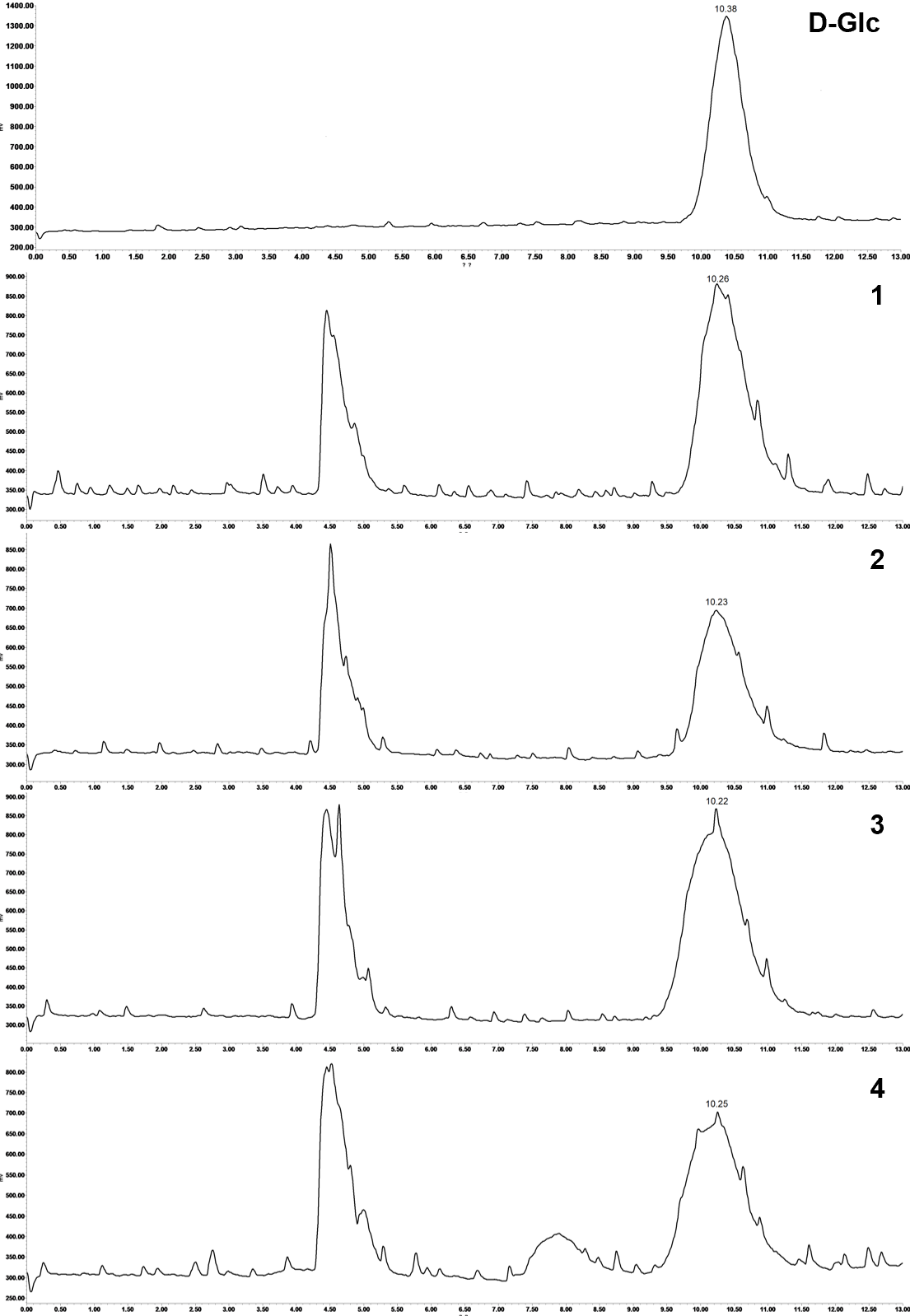


**Figure S109.** The HPLC spectra of the acid hydrolysates of **1**–**4**

Column: Kaseisorb LC NH_2_-60-5 (4.6 mm I.D. × 250 mm); mobile phase: CH_3_CN-H_2_O = 80:20 (v/v); column temperature: 30°C; flow rate: 0.8 mL/min; injection volume: 10*µ*L.

#

**Figure S110.** The toxicity ofcompounds **1**–**19** at the concentration of 50 μM on HaCaT cells

Cell viability: percentage of normal group (set as 100%). Values represent the mean ± SD of six determinations. ****P* < 0.001; **P* < 0.05 (Differences between compound-treated group and normal group) Final concentration was 50 μM for compounds**1**–**19.**

**Figure S111.** The raw data of Figure 9.

**Figure S112.** The raw data of Figure 10.

**Figure S113.** The raw data of Figure 11.
